# Full Genome Sequence Analysis of Two Isolates Reveals a Novel *Xanthomonas* Species Close to the Sugarcane Pathogen *Xanthomonas albilineans*

**DOI:** 10.3390/genes6030714

**Published:** 2015-07-23

**Authors:** Isabelle Pieretti, Stéphane Cociancich, Stéphanie Bolot, Sébastien Carrère, Alexandre Morisset, Philippe Rott, Monique Royer

**Affiliations:** 1 CIRAD UMR BGPI, TA A-54/K, Campus International de Baillarguet, F-34398 Montpellier Cedex 5, France; E-Mails: isabelle.pieretti@cirad.fr (I.P.); stephane.cociancich@cirad.fr (S.C.); alexandre.morisset@outlook.com (A.M.); philippe.rott@cirad.fr (P.R.); 2 INRA, Laboratoire des Interactions Plantes-Microorganismes (LIPM), UMR441, 24 Chemin de Borde Rouge—Auzeville CS52627, F-31326 Castanet Tolosan Cedex, France; E-Mails: stephaniebolot@yahoo.fr (S.B.); sebastien.carrere@toulouse.inra.fr (S.C.); 3 CNRS, Laboratoire des Interactions Plantes-Microorganismes (LIPM), UMR2594, 24 Chemin de Borde Rouge—Auzeville CS52627, F-31326 Castanet Tolosan Cedex, France

**Keywords:** Xanthomonas albilineans, novel “Xanthomonas pseudalbilineans” species, MLSA, ANI

## Abstract

*Xanthomonas albilineans* is the bacterium responsible for leaf scald, a lethal disease of sugarcane. Within the *Xanthomonas* genus, *X. albilineans* exhibits distinctive genomic characteristics including the presence of significant genome erosion, a non-ribosomal peptide synthesis (NRPS) locus involved in albicidin biosynthesis, and a type 3 secretion system (T3SS) of the *Salmonella* pathogenicity island-1 (SPI-1) family. We sequenced two *X. albilineans*-like strains isolated from unusual environments, *i.e.*, from dew droplets on sugarcane leaves and from the wild grass *Paspalum dilatatum*, and compared these genomes sequences with those of two strains of *X. albilineans* and three of *Xanthomonas sacchari*. Average nucleotide identity (ANI) and multi-locus sequence analysis (MLSA) showed that both *X. albilineans*-like strains belong to a new species close to *X. albilineans* that we have named “*Xanthomonas pseudalbilineans*”*. X. albilineans* and “*X. pseudalbilineans*” share many genomic features including (i) the lack of genes encoding a hypersensitive response and pathogenicity type 3 secretion system (Hrp-T3SS), and (ii) genome erosion that probably occurred in a common progenitor of both species. Our comparative analyses also revealed specific genomic features that may help *X. albilineans* interact with sugarcane, e.g., a PglA endoglucanase, three TonB-dependent transporters and a glycogen metabolism gene cluster. Other specific genomic features found in the “*X. pseudalbilineans*” genome may contribute to its fitness and specific ecological niche.

## 1. Introduction

Leaf scald disease of sugarcane (*Saccharum* spp.) is caused by *Xanthomonas albilineans* (Ashby) Dowson—a xylem-invading pathogen. This bacterial disease was first recorded in 1911 in Australia [1]. Today, leaf scald disease occurs in numerous locations worldwide where sugarcane is grown and where it can cause large yield losses in susceptible cultivars (*Saccharum* interspecific hybrids) [2,3]. Disease symptoms vary from a single, narrow, sharply defined white stripe to complete bleaching and necrosis of infected leaves resulting in desiccation, scalding and plant death [3]. First isolated in 1920 by Wilbrink, *X. albilineans* is a vascular systemic pathogen that can colonize the roots, stalks and leaves of sugarcane [4]. *X. albilineans* is a representative of the genus *Xanthomonas*, members of which are exclusively Gram-negative plant-associated bacteria that collectively cause dramatic damage to hundreds of plant species of ornamental or agronomical interest. A recent highly resolved phylogenetic tree of the *Xanthomonadales*, based on 25 concatenated conserved proteins, confirmed anterior studies showing that *X. albilineans* belongs to the same clade as *Xanthomonas translucens*, *Xanthomonas sacchari* and *Xanthomonas hyacinthi* [5].

Previous studies showed that *X. albilineans* has experienced a genome reduction and exhibits distinctive genomic features compared to other species of *Xanthomonas* [6,7,8,9]. For example, the genome of *X. albilineans* lacks two loci required for pathogenicity in other plant pathogenic species of *Xanthomonas*: the xanthan gum biosynthesis, and the hypersensitive response and pathogenicity type 3 secretion system (Hrp-T3SS) gene clusters. In contrast, although reduced in size, the genome of *X. albilineans* encodes specific genomic features including a T3SS of the *Salmonella* pathogenicity island-1 (SPI-1) family and six non-ribosomal peptide synthesis (NRPS) loci including one directing albicidin biosynthesis [8,9]. Albicidin is a DNA gyrase inhibitor acting as both the phytotoxin responsible for leaf scald symptoms and an antibiotic contributing to the competitiveness of *X. albilineans* with other bacteria spreading in sugarcane [10]. Albicidin has some unique structural features [11] and its mode of action differs from that of other DNA gyrase inhibitors [12].

*X. albilineans* exhibits large intra-species genetic variability, with the existence of numerous haplotypes having been described previously using different methodologies including pulsed-field gel electrophoresis (PFGE) and multi-locus sequence analysis (MLSA) [7,13]. Additionally, three serotypes associated with antigenic variations within *X. albilineans* were detected using three antisera (polyclonal antibodies) prepared against strains from three different geographical locations. Serotyping of 215 strains from 28 worldwide locations affected by sugarcane leaf scald disease distributed strains into three groups according to serotype: (i) serotype 1 represents the largest group, with strains from various geographic locations; (ii) serotype 2 consists of strains from tropical African countries; and (iii) serotype 3 contains strains from Caribbean islands (Guadeloupe, Martinique, Saint-Kitts) as well as from Asia (Sri Lanka) and Oceania (Fiji) [14,15]. This serological characterization of *X. albilineans* strains has been corroborated using a combination of monoclonal antibodies on 38 strains from different locations worldwide [16].

Although *X. albilineans* is transmitted mainly via symptomless infected setts and infested cutting implements [17], aerial transmissions were recorded in the 1990s in Guadeloupe and in Mauritius [4,18,19]. Thereafter, aerial transmission and an epiphytic phase were proposed as important steps in the epidemiological cycle of leaf scald disease [20]. To better understand aerial transmission and the epiphytic survival of this sugarcane pathogen, a study was conducted in Guadeloupe in 1997 in experimental plots set up with disease-free tissue cultured sugarcane in a banana-growing location, distant from any other sugarcane field [19]. Thirteen weeks after planting, and during a two-day weather tropical disturbance, a strain belonging to *X. albilineans* serotype 3 (XaS3) was isolated from dew droplets on sugarcane leaf surfaces [19] (this isolate was stored and recorded in CIRAD’s collection as strain GPE 39). Five weeks later, at least half of the experimental sugarcane field canopy was found to be invaded by strains XaS3. During the same time frame, the population density of strains XaS3 on the surfaces of leaves gradually increased. A subsequent decrease in the population density of strains XaS3 on the leaf surface was correlated with the appearance—once again shortly after a tropical storm—and expansion of a second aerially transmitted strain belonging to *X. albilineans* serotype 1 (XaS1) [19] (this isolate was stored and recorded in CIRAD’s collection as strain GPE 40). Unlike GPE 40, GPE 39 was unable to penetrate sugarcane leaves or to colonize sugarcane stalks. GPE 39 also failed to induce any leaf scald symptoms on leaves after artificial inoculation performed in greenhouse experiments, leading the authors to consider it as a non-aggressive epiphyte strain.

In 1940, during the hot and rainy season in an experimental station in Mauritius, a strain resembling *X. albilineans* was isolated from the wild grass *Paspalum dilatatum* Poir. [21]. The infected plants showed leaf symptoms similar to those of leaf scald disease of sugarcane. When inoculated on sugarcane, the *Paspalum* pathogen was able to cause leaf scald symptoms but was unable to spread systemically in the plant. According to its pathogenicity traits and its cultural and biochemical characteristics, the *Paspalum* pathogen was considered as a variety of *X. albilineans*, and was named *X. albilineans* var. *paspali* [21]. This strain isolated on *Paspalum* in Mauritius in 1940 is stored in CIRAD’s collection under the reference MUS 060.

At first glance, the cultural, biochemical, serotyping and/or phenotypic characteristics of the MUS 060 and GPE 39 isolates, as determined in previous studies, led researchers to consider both strains as belonging to the species *X. albilineans* [19,21,22]. In order to deepen and refine the taxonomic characterization of these isolates, we sequenced their genomes and compared the resulting draft sequences to sequence data from the genome of two *X. albilineans* strains representative of the two main clades (MLSA-1 and MLSA-2) of this species, namely strain Xa23R1, for which a draft genome sequence was obtained in the frame of this study, and strain GPE PC73, for which a finished genome sequence has already been published [6,7]. Our comparative analyses also included genome sequences of *Xanthomonas sacchari*—a well-characterized species previously identified as phylogenetically closest to *X. albilineans* [5,23,24]. More precisely, we used the published draft genome sequences of strain LMG 476 isolated from a sugarcane stem [25], of strain NCPPB4393 isolated from an insect collected on a banana [26,27], and of strain R1 isolated from a rice seed [28]. The comparative phylogenetic and genomic analyses described in the present study refines the taxonomic classification of strains MUS 060 and GPE 39 and provides additional data that shed light on the evolutionary history of the sugarcane pathogen *X. albilineans*.

## 2. Results and Discussion

### 2.1. Taxonomic Characterization of MUS 060 and GPE 39 Isolates

**Figure 1:**
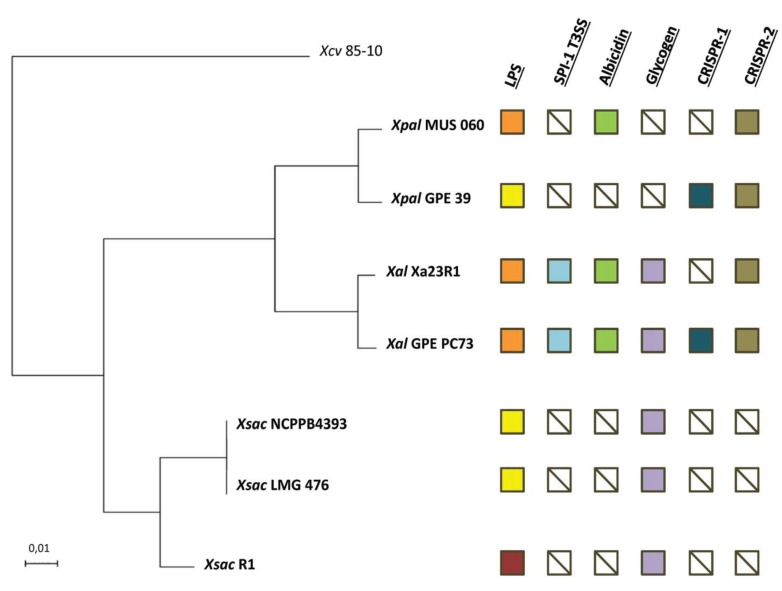
Phylogenetic relationship and overview of some variable genomic regions between strains of *Xanthomonas albilineans* (*Xal*), “*Xanthomonas pseudalbilineans*” (*Xpal*) and *Xanthomonas sacchari* (*Xsac*). Multi-locus sequence analysis (MLSA) tree based on seven concatenated housekeeping gene sequences generated using the maximum likelihood method. *Xanthomonas axonopodis* pv. *vesicatoria* strain 85-10 (*Xcv*) was used as outgroup. All branches were supported by bootstrap values of 100% after 500 replicates. The scale bar indicates the fraction of substitution per site. Orange and yellow squares indicate the presence of lipopolysaccharide (LPS) loci LPS1 or LPS3, respectively. The dark red square corresponds to the LPS locus of strain R1 of *X. sacchari*, which does not share any gene with loci LPS1 or LPS3. Other squares indicate the absence (crossed white) or presence of type 3 secretion system of the *Salmonella* pathogenicity island-1 family (SPI-1 T3SS; blue), albicidin (green), glycogen (violet) and clustered regularly interspaced short palindromic repeats (CRISPRs) (dark blue or brown) loci.

Comparison of complete 16S rRNA gene sequences revealed a nucleotide identity of over 99.7% between *X. albilineans* strains and MUS 060 or GPE 39 strains, and 98.6% when comparing *X. albilineans* strains to *X. sacchari* strains (Supplementary File S1). As previously reported for other xanthomonads [23], comparison of the 16S–23S ITS revealed somewhat more variability than with 16S rRNA, with nucleotide identities of 94.4% and 98.4% when comparing MUS 060 or GPE 39 isolates with *X. sacchari* and *X. albilineans* strains, respectively. Nucleotide identities between MUS 060 and GPE 39 reached 99.9% and 100% when considering 16S rDNA and 16S–23S ITS, respectively, suggesting that both strains belong to the same species (Supplementary File S2). Most taxonomists consider that a 16S rRNA gene nucleotide sequence identity below 97% means that the compared sequences belong to different species; however, the meaning of identity greater than 97% remains unclear, partly due to the variable evolutionary histories of bacterial species [29,30]. Nevertheless, assuming an ideal nucleotide identity threshold of 99.5% for species identification [31], the values for 16S rRNA gene sequence identity between MUS 060 or GPE 39 *versus* *X. albilineans* strains (~99.7%) suggest that MUS 060 and GPE 39 belong to the species *X. albilineans*. To further confirm or refute this taxonomic classification, we performed whole-genome-based comparisons using the ANI method [32], which provides a higher taxonomic resolution equivalent to that of DNA-DNA hybridization (DDH) methods [33]. ANI values between MUS 060 or GPE 39 and both *X.* *albilineans* strains reached 91% at most, whereas those between MUS 060 or GPE 39 and each of the *X. sacchari* strains were below 87% (Supplementary File S3), showing unambiguously that strains MUS 060 and GPE 39 do not belong to the *X. albilineans* species. However, since it is commonly held that an ANI threshold of 95%–96% is equivalent to a DDH threshold of 70% for species identification [32,34], the genomes of MUS 060 and GPE 39 are closer to those of *X. albilineans* than to *X. sacchari*. MUS 060 and GPE 39 sequences display a genomic identity of ~96% (ANI), suggesting that both strains belong to the same species, thus together belonging to a new species within the *Xanthomonas* genus. The ANI results were confirmed by our MLSA phylogenetic analysis and its resulting robust MLSA tree, which groups MUS 060 and GPE 39 in a clade distinct from that containing the *X. albilineans* strains (Figure 1). As a result, strains MUS 060 and GPE 39, first assigned by their discoverers to *X. albilineans*, belong *in fine* to a novel *Xanthomonas* species not described to date, leading us to reconsider *X. albilineans* var*.* *paspali* strain MUS 060 and *X. albilineans* strain GPE 39 as representatives of this novel species that we propose to name “*X. pseudalbilineans*” (not valid name).

### 2.2. Orthology Analysis

**Figure 2:**
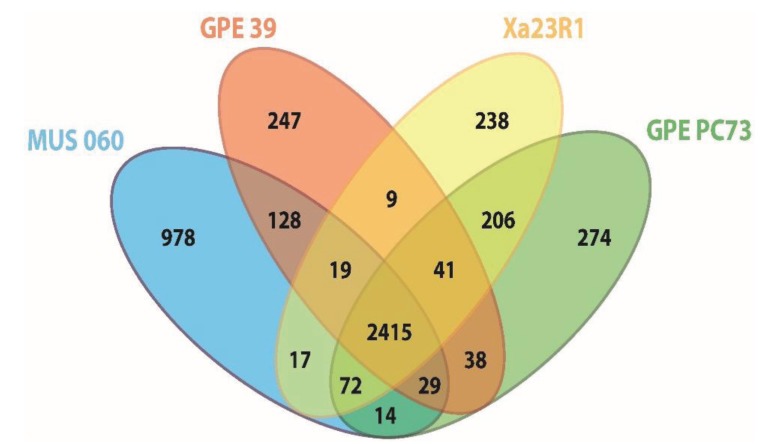
Venn diagram showing the number of orthologous proteins (OrthoMCL groups) shared by proteomes of strains of *X. albilineans* and “*X. pseudalbilineans*” species (numbers shown do not include paralogous protein sequences). The number of specific elements of each proteome is the sum of specific inparalog groups and of specific single copy proteins of this proteome. GPE PC73: *X. albilineans* strain GPE PC73, Xa23R1: *X. albilineans* strain Xa23R1, GPE 39: “*X. pseudalbilineans*” strain GPE 39 and MUS 060: “*X. pseudalbilineans*” strain MUS 060.

OrthoMCL analyses based on pairwise comparisons [35] were performed in order to identify orthologous groups shared between *X. albilineans* strains Xa23R1 and GPE PC73 and “*X. pseudalbilineans*” strains MUS 060 and GPE 39. The four strains share 2415 “core” orthologous protein sequences or coding DNA sequences (CDSs; Figure 2) and each strain possesses a similar number of unique CDSs, with the notable exception of strain MUS 060 for which a higher number of specific CDSs was identified. In this analysis, the core genome represents 66%–80% of each of the four genomes. This value is similar to the one estimated from comparison of *X. axonopodis* pv. *citrumelo* strain F1, *X. campestris* pv. *vesicatoria* strain 85–10 and *X. axonopodis* pv. *citri* strain 306, *i.e.*, about three-quarters of each total genome [36]. When performing such a comparative analysis with a larger number of xanthomonads, the core genome size represented on average 30% of any genome of the 13 strains analyzed, and this value increased to 44% when *X. albilineans* was excluded from the analysis [37].

Orthology comparisons identified 206 CDSs specific to *X. albilineans* strains GPE PC73 and Xa23R1, and 128 CDSs specific to “*X. pseudalbilineans*” strains MUS 060 and GPE 39. Upon excluding CDSs from the core genome and those specific to each of the four strains, only a very small number of CDSs remain that are shared by at least one strain of *X. albilineans* and one strain of “*X. pseudalbilineans*”, as illustrated for instance by the low number of CDSs shared by both *X. albilineans* strains with either “*X. pseudalbilineans*” strain MUS 060 or strain GPE 39 (72 and 41 CDSs, respectively) (Figure 2). Both species are indeed closely related, “*X. pseudalbilineans*” sharing the main *X. albilineans* genomic features compared to other xanthomonads, such as the lack of *gum*, Hrp T3SS and Type 6 secretion system gene clusters.

### 2.3. Serotypes and Variability of LPS Loci

**Figure 3:**
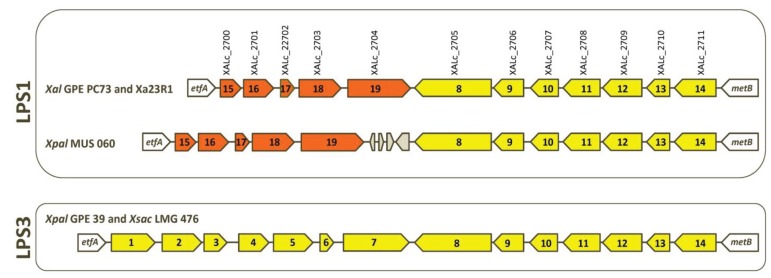
Schematic comparison of LPS loci of strains of *X. albilineans* (*Xal* GPE PC73 and *Xal* Xa23R1), “*X. pseudalbilineans*” (*Xpal* GPE 39 and *Xpal* MUS 060) and *X. sacchari* (*Xsac* LMG 476). An arrow with a same color and a same number represents an identical gene. Grey-colored arrows are transposase sequences.

Lipopolysaccharides (LPS) are involved in biofilm formation and pathogenicity of numerous Gram-negative pathogenic bacteria. LPS components on the cell surface of plant-pathogenic *Xanthomonas* species can be recognized as pathogen-associated molecular patterns (PAMPs) factors that activate the basal response of the infected plant (see, e.g., [38,39]). Within the LPS locus of most xanthomonads, numerous horizontal gene transfer (HGT) events have resulted in chimeric LPS loci and in a large diversification of this locus between strains (at both inter- and intra-species levels) [40], thus probably affecting recognition of these molecules as PAMPs by the host plant [41]. Analysis of the LPS loci of the two *X. albilineans* (GPE PC73 and Xa23R1) and the two “*X. pseudalbilineans*” (MUS 060 and GPE 39) strains revealed two distinct LPS loci (Figure 3). By analogy with descriptions of numerous other bacteria [42], antigenic variations in serotypes 1 and 3 are most probably associated with the existence of these two LPS loci. Strains belonging to serotype 1 (GPE PC73, Xa23R1 and MUS 060) share the same LPS locus: LPS1, whereas the strain belonging to serotype 3 (GPE 39) exhibits another LPS locus: LPS3 (nucleic acid sequence of genes specific to the LPS3 locus of strain GPE 39 is provided in Supplementary File S4). As with all xanthomonads sequenced to date, both LPS clusters are located between the highly conserved *etfA* and *metB* genes. The variable portion that differentiates LPS1 from LPS3 may contain genes involved in the mechanism of serotype specificity for these species. Locus LPS3 contains 14 genes, all of which are conserved in the LPS locus of *X. sacchari* excluding strain R1, which possesses a specific LPS locus (Figure 3). The LPS1 locus contains five specific genes and shares seven genes with LPS3 (Figure 3). Interestingly, these seven genes are also conserved in the LPS locus of *Xanthomonas* *campestris* pv. *vasculorum*—a phylogenetically distant species that is also pathogenic on sugarcane—and five of them are conserved in the LPS locus of *Xanthomonas campestris* pv. *campestris* B100 [7]. Locus LPS1 of MUS 060 contains specific insertion- and phage-related sequences located between orthologous genes XALc_2704 and XALc_2705 from strain GPE PC73. Most probably, these do not interfere with expression of other genes of the locus (Figure 3).

### 2.4. The Albicidin Locus and other NRPS Loci

NRPS is a pathway based on multimodular megasynthetases used by bacteria and fungi to produce peptides in a ribosome-independent manner [43]. The NRPS albicidin locus is present in the “*X. pseudalbilineans*” strain MUS 060 draft genome but not in the genome of the “*X. pseudalbilineans*” strain GPE 39, confirming the results of albicidin production bioassays (data not shown). The albicidin locus of strain MUS 060 contains all the albicidin biosynthesis genes described in *X. albilineans*, except *alb18* encoding an aminodeoxychorismate lyase, which is most likely complemented by a gene present elsewhere in the genome encoding the same enzyme (ortholog of gene XALc_0280 in strain GPE PC73). Interestingly, *alb18* is also partially frameshifted in *X. albilineans* strains GPE PC73 and Xa23R1, supporting the hypothesis of complementation with the ortholog of XALc_0280. HPLC analysis of an albicidin extract from strain MUS 060 showed a chromatographic profile very similar to those usually observed for extracts of *X. albilineans* strains [11]; peaks containing albicidin antibiotic activity eluted at similar retention times for both species. The absence of the albicidin locus in strain GPE 39 is in accordance with the inability of this isolate to produce albicidin. The gene encoding the phosphopantetheinyl-transferase required for albicidin production and for post-translational activation of NRPS megasynthetases, which is present outside the albicidin locus, is conserved in strains MUS 060, GPE 39, Xa23R1 and GPE PC73 and is located at the same position in these genomes (between orthologs of XALc_1735 and XALc_1737).

Previous genome mining data has led to the identification of a new NRPS locus, named META-B, present in the genome of five strains: *X. albilineans* strain GPE PC73, *Xanthomonas oryzae* pv. *oryzae* strains BAI3 and X11-5A, *Xanthomonas translucens* strain DAR61454 and “*X. pseudalbilineans*” strain GPE 39 [9]. In addition to NRPS megasynthetases, META-B encodes a transcription regulator of the AraC family, a cyclic peptide transporter, a MbtH-like protein and enzymes involved in biosynthesis of the non-proteinogenic amino acids 2,4-diaminobutyric acid and 3,5-dihydroxyphenylglycine. The function of the small molecule encoded by this locus remains unknown. Despite the similar organization of the META-B locus in all five strains, *in silico* prediction of peptide sequences assembled by NRPS megasynthetases indicates that each strain produces a different lipopeptide. The META-B locus is partially deleted in strain MUS 060; genes encoding the transcription regulator of the AraC family and enzymes involved in the biosynthesis of 3,5-dihydroxyphenylglycine are missing in this strain.

Two short NRPS loci have also been identified on the chromosome of *X. albilineans* strain GPE PC73: they both encode only one NRPS module. Interestingly, there is an overlap between both these genes and a gene encoding a glycosyltransferase. It has been hypothesized that these genes encode glycosylated amino acids, but no precise function for these has yet been attributed [9]. Strains MUS 060 and GPE 39 each possess only one of these two short NRPS loci (orthologs of XALc_0364 and XALc_0365, respectively). The two short NRPS loci are absent in the three strains of *X. sacchari*.

### 2.5. Genes of X. albilineans Linked to a Xylem-Invading Lifestyle

Five cell-wall-degrading enzymes (CWDEs) of *X. albilineans* strain GPE PC73 exhibit specific features compared to their respective orthologs in *X. sacchari* and other sequenced species of *Xanthomonas* [7]. They harbor a cellulose-binding domain (CBD) and a long linker region both predicted to be adapted to the utilization of cell-wall breakdown products as carbon source and to the ability to spread in sugarcane xylem vessels [7]. These specific features are probably important for the ability of *X. albilineans* to spread in xylem and for its pathogenicity. The long linker is rich in proline, threonine, serine, and glycine. Similar CBDs and long linkers have been found in the draft genomes of strains Xa23R1, MUS 060 and GPE 39. However, read misalignments in draft genomes due to the repeated sequence within linkers have led to sequences encoding CBDs and CWDEs being found in separate contigs and it has not been possible to clearly identify the corresponding CBD for each CWDE. *X. albilineans* strains produce an endopolygalacturonase encoded by *pgl*A (XALc_0811), which is missing in both “*X. pseudalbilineans*” strains. This CWDE, which lacks any CBD and long linker, may represent a component of the offensive arsenal of *X. albilineans* allowing it to penetrate and colonize sugarcane xylem vessels.

TonB-dependent transporters (TBDTs) are known to be involved in the uptake of a large variety of substrates, such as cobalamin, iron-siderophore complexes, carbohydrates and organic acids (see e.g., [44,45]). TBDTs may be used by *X. albilineans* to transport cell-wall-degrading products resulting from the activity of CWDEs, and thus facilitate spread of the organism in the nutrient-poor conditions prevailing in sugarcane xylem. In the genome of *X. albilineans* strain GPE PC73, 35 TBDT genes have been identified, including one specific to this species (XALc_2962) and two others (XALc_0643 and XALc_0723) that were functionally associated with pathogenicity of the bacterium [7,46]. These three TBDT genes are conserved in both MUS 060 and GPE 39 strains; however, the notable absence in both MUS 060 and GPE 39 strains of three TBDT genes present in both GPE PC73 and Xa23R1 strains (XALc_2778, XALc_3025 and XALc_1949) is striking. On the other hand, in strains MUS 060 and GPE 39, we identified a TBDT that is absent in both *X. albilineans* strains but similar to those found in *X. sacchari* strain LMG 476 (nucleic acid sequence of this gene is provided in Supplementary File S4).

### 2.6. Variability of the Locus Encoding the Type IV Pilus

Bacterial pili are filamentous flexible cell-surface appendages involved in numerous bacterial virulence processes, including attachment and invasion, biofilm formation, twitching motility, cellular invasion, and transport of protein and DNA across membranes [47,48]. Biogenesis of a type IV pilus involves a large number of proteins, with PilA representing the major pilin subunit and thus the main structural protein of the type IV pilus. The sequence variability in the *pilA* gene observed among sequenced *Xanthomonas* could be correlated with host specificity [49]. In *Xanthomonas fuscans* subsp. *fuscans*, PilA is involved in adhesion and transmission to seeds, and mutation of *pilA* results in reduced pathogenicity on bean [50]. It has also been reported that this variability could be due to the selective pressure exerted by phages, leading to optimization of plant interactions and bacterial infection by phages [51,52,53]. All genes within the locus *pil*DCABRS exhibit higher amino acid similarity between *X. albilineans* strains GPE PC73 and Xa23R1 than with both MUS 060 and GPE 39 isolates, except for gene *pil*A, for which an even stronger polymorphism is observed between *X. albilineans* strains. PilA protein of strains GPE PC73 and GPE 39 share 77% similarity, whereas PilA from Xa23R1 is highly divergent from that of strains GPE PC73, GPE 39 and MUS 060 at both nucleic acid and amino acid sequence levels. Furthermore, PilA identified in the MUS 060 genome exhibits no significant similarity with PilA encoded by GPE PC73, Xa23R1 and GPE 39. The complete *pil*VWXYE gene cluster exhibits higher amino acid similarity between *X. albilineans* strains GPE PC73 and Xa23R1 than with either of the MUS 060 and GPE 39 isolates, whereas this locus in MUS 060 exhibits higher amino acid similarity with the two *X. albilineans* strains than with GPE 39.

### 2.7. CRISPR and Restriction-Modification System Loci

Clustered regularly interspaced short palindromic repeats (CRISPRs) are repetitive structures in bacteria and Archaea composed of exact 24- to 48-bp repeated sequences (“repeats”) separated by unique sequences of similar length (“spacers”). Additionally, CRISPRs are characterized by CRISPR-associated (*cas*) genes and a leader sequence located near the repeats (see e.g., [54,55]). CRISPRs systems are involved in phage and plasmid defense, thus limiting HGT. Sequencing of the genome of *X. albilineans* strain GPE PC73 revealed the presence of two different CRISPR/*cas* systems named CRISPR-1 and CRISPR-2, respectively [7]. The CRISPR-2 locus is associated with six *cas* genes (*cas*1, *cas*3, *csy*1, *csy*2, *csy*3 and *csy*4) and contains 28-bp repeats. CRISPR-2 is shared by the four strains of this study (*X. albilineans* strains GPE PC73 and Xa23R1 and “*X. pseudalbilineans*” strains GPE 39 and MUS 060), as well as by *Xanthomonas campestris* pv. *raphani* [7,56]. Interestingly, regarding CRISPR-2, the nucleic acid sequences of the repeats are 100% identical among the four strains, thus confirming the common origin of this locus. In contrast, no common CRISPR-2 spacers are shared by the four strains, underlying distinct phage exposure and strain adaptation according to their respective environment. Furthermore, spacers of MUS 060 and GPE 39 isolates show no identity to phages described in GenBank to date. None of the CRISPR-2 spacers of strain MUS 060 exhibit identity with other nucleic acid sequences of its own genome or with genomes of strains GPE PC73, Xa23R1 and GPE 39. Also, none of the CRISPR-2 spacers of strain GPE 39 share identity with other nucleic acid sequences of its own genome, but spacer-2 is 96% identical to spacer-13 of the CRISPR-1 system of GPE PC73, and spacer-54 is 100% identical to a gene sequence located in one of the three plasmids of strain GPE PC73 (XALr_3262). The CRISPR-1 locus, present only in strains GPE PC73 and GPE 39, is associated with seven *cas* genes (*cas*3, *cas*5d, *csd*1, *csd*2, *cas*4, *cas*1 and *cas*2) and contains 31-bp repeats. The nucleic acid sequences of CRISPR-1 repeats are 100% identical in both strains and all CRISPR-1 spacers from strains GPE PC73 and GPE 39 are specific to each strain. The CRISPR-1 locus is also conserved in other strains belonging to *X. oryzae* pv. *oryzae*, *X. axonopodis* pv. *citri*, *X. campestris* pv. *vasculorum* or *X. campestris* pv. *musacearum* [7].

The genomes of strains GPE PC73 and GPE 39 both contain distinct restriction-modification systems that also could contribute to resistance against phages. Strain GPE PC73 possesses a restriction enzyme (XALc_2635) associated with a *Xam*I N6-adenine methyltransferase (XALc_2634). Strain GPE 39 possesses two other type II restriction endonucleases: (i) *Pae*R71 associated with N4/N6 methyltransferase *Pae*R71, which is 100% identical to those found in a *X. axonopodis* strain (GenBank Accession No. WP_017173568 and WP_017173569, respectively) and (ii) another type II restriction endonuclease associated with a C-5 cytosine methyltransferase (nucleic acid sequence of these genes is provided in Supplementary File S4). The presence of such restriction-modification systems as well as of CRISPR-1 in both GPE PC73 and GPE 39, but not in Xa23R1 and MUS 060, suggests that strains GPE PC73 and GPE 39 are better adapted to both phage-containing environments and epiphytic survival.

### 2.8. Other Genes or Loci Specific to X. albilineans

Among the 206 genes that are conserved in the two strains of *X. albilineans* and absent in “*X. pseudalbilineans*” strains (*i.e.*, genes specific to the *X. albilineans* species) are genes encoding the SPI-1 T3SS. This particular secretion system, usually found in mammals and insect bacterial pathogens or symbionts, exhibits high similarity to one described in *Burkholderia pseudomallei* [8,57]. Although the SPI-1 T3SS is not associated directly with the pathogenicity of *X. albilineans* based on our knockout mutant analysis [8], it may nevertheless be required for bacterium-plant interaction, in particular to establish plant colonization, as reported for *Salmonella* *typhimurium* in *Arabidopsis thaliana* [58].

The MUS 060 and GPE 39 genomes also lack a locus encoding glycogen biosynthesis. In strain GPE PC73, this locus comprises six genes: *glg*A (XALc_2591), *glg*B (XALc_2592), a gene encoding a glucanotransferase (XALc_2593), *glg*Y (XALc_2594), a short gene with unknown function (XALc_2595), and *glg*X (XALc_2596). Compared to the glycogen metabolism locus usually found in other xanthomonads, *glg*C and *glg*P genes are missing in *X. albilineans* genomes [59]. The *glg* locus is missing in both “*X. pseudalbilineans*” strains, as is also the case for *Xanthomonadales* *Xylella fastidiosa* strain 9a5c and *Stenotrophomonas maltophilia* strain R551-3 [59]. HGT events have been suggested to play an important role in the evolutionary history of *glg* genes and these latter genes may confer some advantages to the bacteria for better survival under varying environmental conditions [59]. Although glycogen is not an essential component for bacterial growth or survival, the loss of *glg* genes from the glycogen pathway may reduce the survival time of bacteria when placed in an environment lacking an exogenous carbon source. It has been reported previously that the lack of bacterial glycogen metabolism is a trait associated with parasitic or symbiotic behavior of bacteria [60]. The gene encoding a helicase found in *X. albilineans* strains (XALc_3107 in GPE PC73) and in other xanthomonads including *X. sacchari* LMG 476, is missing in both “*X. pseudalbilineans*” strains. An additional 155 CDSs that encode hypothetical proteins or proteins with diverse functions are also missing in strains MUS 060 and GPE 39.

### 2.9. Other Genes or Loci Specific to “X. pseudalbilineans”

Strains MUS 060 and GPE 39 share 128 CDSs encoding hypothetical proteins or proteins with diverse functions, among which are three specific methyltransferases not associated with a cognate restriction endonuclease. However, these orphan methyltransferases may play a role in regulatory processes of isolates MUS 060 and GPE 39 in functions such as replication, transcription, DNA repair and population evolution [61].

Among the 977 genes specific to strain MUS 060, the following are of particular interest (nucleic sequence of these genes is provided in Supplementary File S4): (i) seven clustered genes involved in surface polysaccharide biosynthesis. These genes are identical to those identified in *X. sacchari* LMG 476 genome, exhibiting 80%–89% nucleic acid sequence identity. According to their protein domains, the encoded proteins are predicted to correspond to a glycosyl transferase, a polysaccharide biosynthesis protein, a pyridoxal phosphate-dependent transferase, an acyl-CoA *N*-acyltransferase, a glycosyl hydrolase, a sugar *O*-acyltransferase and a dTDP-6-deoxy-3,4-keto-hexulose isomerase. (ii) a large CDS (1450 amino acids) that shows no significant amino acid similarity to other bacteria found in GenBank, but which consists of a *C*-terminal autotransporter domain associated with an *N*-terminal passenger domain as well as four autotransporter-associated beta-stranded repeat domains and a left-handed parallel beta-helix (LbH) domain. The presence of such domains, usually found in autotransporter proteins of the type V secretion system of Gram-negative bacteria, suggests a possible adhesion function associated with this CDS; however, the involvement of this protein in bacterial virulence and attachment to leaves remains to be demonstrated.

### 2.10. Genome Erosion

A previous OrthoMCL study identified 592 ancestral genes present in strain 85-10 of *X*. *axonopodis* pv. *vesicatoria* that are absent in strain GPE PC73 and considered as genes lost by *X. albilineans* during its speciation [6]. BLAST analysis showed that 359 of these 592 genes are present in *X. sacchari*. A new OrthoMCL analysis, including strain 85-10 of *X.* *axonopodis* pv. *vesicatoria*, indicated that 295 of these 359 CDSs are absent in both MUS 060 and GPE 39 strains. Considering the MLSA tree presented in Figure 1, the 295 CDSs present in *X. sacchari* strain LMG 476 and absent in *X. albilineans* and “*X. pseudalbilineans*” strains most probably correspond to ancestral genes lost by a common ancestor of these two species. The chromosome size of strain GPE PC73 is 3.8 Mb and the genome sizes of strains Xa23R1, MUS 060 and GPE 39, estimated on the basis of draft genome sequences, are 3.5 Mb, 3.9 Mb and 3.5 Mb, respectively. The G + C content of *X. albilineans* and “*X. pseudalbilineans*” genomes is 63%, while the G + C content of *X. sacchari* strain LMG 476 genome is 69% [25]. Genome erosion is associated with elevated frequencies of adenine and thymine (A + T) in obligate host-associated bacteria (see e.g., [62,63]). A lower G + C content for *X. albilineans* and “*X. pseudalbilineans*” species supports the hypothesis that genome erosion occurred in a common ancestor of these two species. Acquisition of the albicidin locus was postulated to be one of the triggers for genome reduction in the ancestor of *X. albilineans*; Pieretti *et al.* [6] proposed that albicidin production in the *X. albilineans* ancestor induced the SOS response to bypass DNA damage occurring from gyrase inhibition and thus caused genome rearrangements and mutation. Genome erosion eventually stopped when the gene encoding the bacterial DNA gyrase mutated, conferring resistance to the toxin [6]. The presence of the albicidin locus in strain MUS 060 suggests that this locus was present in the common ancestor of *X. albilineans* and “*X. pseudalbilineans*” and supports the hypothesis of a link between acquisition of the albicidin locus and genome erosion. Further experimental studies will be required to study the susceptibility to albicidin of the DNA gyrase of *X. sacchari* in order to explore this hypothesis.

## 3. Experimental Section

### 3.1. Bacterial Strains and DNA Preparation

Characteristics of the bacterial strains used in this study are summarized in Table 1. Pure cultures of bacterial strains GPE 39, MUS 060 (NCPPB 208) and Xa23R1 stored at CIRAD’s collection (Montpellier, France) were grown for 48 h at 28 °C in Wilbrink’s liquid medium [64] for isolation of total genomic DNA using a Qiagen DNeasy Tissue extraction kit (Qiagen SA, Courtaboeuf, France) according to the manufacturer’s recommendations.

**Table 1:** Description of *Xanthomonas* strains analyzed.

Organism	Geographical Origin	Date of Isolation	Biological Origin	GenBank Accession n°	Reference
*X. albilineans* GPE PC73	Caribbean/Guadeloupe	2006	Sugarcane (*Saccharum* spp.)	GCA_000087965.1	[6]
*X. albilineans* Xa23R1	USA/Florida	1993	Sugarcane (*Saccharum* spp.)	JZIK00000000	[65]
“*X. pseudalbilineans*” MUS 060	Indian ocean/Mauritius	1940	Dallis grass (*Paspalum dilatatum* Poir.)	JZIM00000000	[21]
“*X. pseudalbilineans*” GPE 39	Caribbean/Guadeloupe	1997	Water droplets collected on leaves of sugarcane	JZHZ00000000	[19]
*X. sacchari* LMG 476	Caribbean/Guadeloupe	1980	Sugarcane (*Saccharum* spp.)	JXQE00000000	[25]
*X. sacchari* NCPPB4393	Africa/Tanzania	2007	Insect collected on a banana plant (*Musa* spp.)	AGDB00000000	[26]
*X. sacchari* R1	China/Heilongjiang	2011	Asymptomatic seed of rice (*Oriza sativa*)	GCA_000815185.1	[28]

### 3.2. DNA Sequencing and Assembly

Shotgun sequencing of strains Xa23R1 and GPE 39 was performed on a Solexa GAIIx (Illumina) sequencer (Genoscope, Évry, France), yielding 35,491,178 and 32,445,653 76-bp paired-end reads, respectively, with an insert size of 200 bp. Whole genome shotgun (WGS) BioProject sequences of strains Xa23R1 and GPE 39 have been deposited with the BioProject/GenBank databases under the references PRJNA270034 and PRJNA270166, respectively.

Shotgun sequencing of strain MUS 060 was performed on a MiSeq 2000 sequencer (Fasteris, Geneva, Switzerland), yielding 3,139,946 125-bp paired-end reads. WGS BioProject of strain MUS 060 has been deposited with the BioProject/GenBank database under the reference PRJNA270312.

The draft assemblies of strains Xa23R1, GPE 39 and MUS 060 are available from the DDBJ/EMBL/GenBank databases under accession numbers JZIK00000000, JZHZ00000000 and JZIM00000000, respectively.

We used a combination of Velvet [66], SOAPdenovo and SOAPGapCloser [67] to assemble the three strains Xa23R1, GPE 39 and MUS 060. This protocol yielded 4, 8 and 126 scaffolds, respectively, larger than 500 bp, and a largest scaffold of 3,529,636 bp, 3,511,121 bp, and 670,305 bp (using *X. albilineans* GPE PC73 as a reference for guided scaffolding final step for Xa23R1 and GPE 39) for a total assembly size of 3,538,317 bp, 3,528,360 bp and 3,857,307 bp (excluding scaffolding gaps).

### 3.3. Genotyping with 16S–23S rRNA ITS and 16S rRNA Gene Sequence

16S ribosomal RNA (rRNA) gene sequences and the 16S–23S rRNA internal transcribed spacer region (ITS) of strains MUS 060, GPE 39, *X. sacchari* LMG 476, *X. sacchari* NCPPB4393, *X. sacchari* R1 and *X. albilineans* Xa23R1 were searched on their respective draft genome sequences (see corresponding accessions in Table 1) using blastn (http://blast.ncbi.nlm.nih.gov/blast/Blast.cgi?CMD=Web&PAGE_TYPE=BlastHome [68]) with the query being the 16S ribosomal DNA sequence provided by the reference strain GPE PC73. The seven resulting sequences were compared using the MAFFT alignment program (http://mafft.cbrc.jp/alignment/software/ [69] (see sequences and alignments in Supplementary Files S1 and S2, respectively).

### 3.4. Phylogenetic Core Genome Analysis: MLSA

MLSA based on seven complete housekeeping genes (*atp*D, *dna*K, *efp*, *gln*A, *gro*L, *gyr*B and *rec*A) was performed on MUS 060 and GPE 39 isolates, on *X. sacchari* strains (strains NCPPB4393, R1 and LMG 476), on *X. albilineans* strains (strains GPE PC73 and Xa23R1) as well as on *Xanthomonas axonopodis* pv. *vesicatoria* strain 85–10 (see Table 1 for references and GenBank accessions of available genome sequences). Alignment of concatenated nucleotide gene sequences performed with MAFFT alignment program using default parameters [69] resulted in a 10,490-bp long sequence (including gaps) (see alignment in Supplementary File S5). The phylogenetic core genome MLSA tree was generated using the maximum likelihood method implemented in the PhyML program version 3 [70] with GTR as substitution model and four gamma-distributed rate categories to account for rate heterogeneity across sites. Five hundred bootstrap replicates were performed and *X. axonopodis* pv. *vesicatoria* strain 85-10 was used to root the consensus tree.

### 3.5. Whole-Genome Sequences Analyses: ANI Calculation

Genomic relatedness between strain MUS 060, strain GPE 39, *X. albilineans* strains GPE PC73 and Xa23R1, and *X. sacchari* strains NCPPB4393, R1 and LMG 476 was estimated using whole-genome sequencing data of each strain. Average nucleotide identity (ANI) was calculated with ANIb and ANIm software, respectively, based on BLASTN and MUMmer algorithms using the tool JSpecies and default parameters (http://www.imedea.uib.es/jspecies/ [32]) (see ANI scores in Supplementary File S3).

### 3.6. OrthoMCL Analyses

Identification of orthologous groups between Xa23R1, GPE 39, MUS 060 and GPE PC73 genomes was achieved by orthoMCL analyses [35]. OrthoMCL clustering analyses were performed using the following parameters: *p*-value Cut-off = 1 × 10^−5^; Percent Identity Cut-off = 0; Percent Match Cut-off = 80; MCL Inflation = 1.5; Maximum Weight = 316. We modified OrthoMCL analysis by inactivating the filter query sequence during the BLASTP pre-process. From results are defined unique CDSs, corresponding to CDSs present only in one copy in one genome, and groups of orthologs that correspond to CDSs present in one copy in at least two genomes. The main part of comparative analyses of genomes and figures are deduced from their distribution. Groups of homologs refer to groups of orthologs having or not having paralogs.

## 4. Conclusions

In this study, we analyzed draft genome sequences of two previously collected isolates belonging to the *Xanthomonas* genus: strain GPE 39 isolated from water droplets on *S. officinarum* leaves in Guadeloupe, and strain MUS 060 collected from *P. dilatatum* in Mauritius. Our comparative study also included genome sequencing data of two strains of *X. albilineans* and three strains belonging to *X. sacchari*. Comparative analyses, including ANI and MLSA analyses, revealed that GPE 39 and MUS 060 belong to a new species. Because GPE 39 and MUS 060 are close to the *X. albilineans* species, with which they form a phylogenetic sister clade, we hereby propose the name “*X. pseudalbilineans*” for this novel species. Compared to *X. sacchari*, *X. albilineans* and “*X. pseudalbilineans*” species have experienced genome erosion that probably occurred predominantly in the common progenitor of both species. Genes encoding the injectisome and associated effectors of the Hrp-T3SS are missing in the genome of *X. sacchari*, *X. albilineans* and “*X. pseudalbilineans*” species. Unlike the sugarcane pathogen *X. albilineans*, which is able to colonize the xylem vessels of sugarcane, previous experimental studies showed that strains GPE 39 and MUS 060 failed to colonize sugarcane stalks systemically [19,21]. The host range specificity and, more broadly, the ecological niche of strains GPE 39 and MUS 060 remain unclear. *X. albilineans* and “*X. pseudalbilineans*” have probably specialized in separate environmental niches. The presence of the same LPS form (LPS1) in sugarcane pathogens (*X. albilineans* strains) and in the *P. dilatatum* pathogen (MUS 060 strain) is in accordance with a previous study showing no apparent correlation between LPS variation and host- or tissue-specificity [71]. Among the set of accessory genes of “*X. pseudalbilineans*” and *X. albilineans* strains, we listed some candidate genomic determinants that may play a role in the life cycle of these bacteria. In depth functional analyses are now required to explore the role of these genes in the successful invasion of sugarcane xylem vessels by *X. albilineans*. Further analyses conducted on a larger collection of strains of “*X. pseudalbilineans*” should help us to better understand the genetic determinants involved in niche-specific adaptation of “*X. pseudalbilineans*” and to complete species demarcation, for which borders with *X. albilineans* are narrow.

## Supplementary Files

Supplementary File 1
