# Supplementary material for: Full Genome Sequence Analysis of Two Isolates Reveals a Novel Xanthomonas Species Close to the Sugarcane Pathogen Xanthomonas albilineans
Source: Genes (Basel). 2015 Jul 23;6(3):714–33. doi: 10.3390/genes6030714 (PMC4584326; doi:10.3390/genes6030714)
Supplement: Supplementary File 1 [file genes-06-00714-s001.zip › genes-85642-supplementary/Supplementary File S1_v2.docx]

**Supplementary File S1**

**16S ribosomal DNA gene sequences alignment and pairwise identity**

**CLUSTAL format alignment by MAFFT (v7.214)**

16S_GPE PC73 agtgaacgctggcggcaggcctaacacatgcaagtcgaacggcagcacagtggtagcaat

16S_Xa23R1 agtgaacgctggcggcaggcctaacacatgcaagtcgaacggcagcacagtggtagcaat

16S_MUS 060 agtgaacgctggcggcaggcctaacacatgcaagtcgaacggcagcacagtggtagcaat

16S_GPE 39 agtgaacgctggcggcaggcctaacacatgcaagtcgaacggcagcacagtggtagcaat

16S_R1 agtgaacgctggcggcaggcctaacacatgcaagtcgaacggcagcacaggagagcttgc

16S_LMG 476 agtgaacgctggcggcaggcctaacacatgcaagtcgaacggcagcacaggagagcttgc

16S_NCPPB4393 agtgaacgctggcggcaggcctaacacatgcaagtcgaacggcagcacaggagagcttgc

************************************************** .* . . ..

16S_GPE PC73 accatgggtggcgagtggcggacgggtgaggaatacatcggaatctaccttttcgtgggg

16S_Xa23R1 accatgggtggcgagtggcggacgggtgaggaatacatcggaatctaccttttcgtgggg

16S_MUS 060 accatgggtggcgagtggcggacgggtgaggaatacatcggaatctacctattcgtgggg

16S_GPE 39 accatgggtggcgagtggcggacgggtgaggaatacatcggaatctacctattcgtgggg

16S_R1 tctctgggtggcgagtggcggacgggtgaggaatacatcggaatctaccttttcgtgggg

16S_LMG 476 tctctgggtggcgagtggcggacgggtgaggaatacatcggaatctaccttttcgtgggg

16S_NCPPB4393 tctctgggtggcgagtggcggacgggtgaggaatacatcggaatctaccttttcgtgggg

*. ********************************************** *********

16S_GPE PC73 gataacgtagggaaacttacgctaataccgcatacgaccttagggtgaaagcggaggacc

16S_Xa23R1 gataacgtagggaaacttacgctaataccgcatacgaccttagggtgaaagcggaggacc

16S_MUS 060 gataacgtagggaaacttacgctaataccgcatacgaccttagggtgaaagcggaggacc

16S_GPE 39 gataacgtagggaaacttacgctaataccgcatacgaccttagggtgaaagcggaggacc

16S_R1 gataacgtagggaaacttacgctaataccgcatacgaccttagggtgaaagcggaggacc

16S_LMG 476 gataacgtagggaaacttacgctaataccgcatacgaccttagggtgaaagcggaggacc

16S_NCPPB4393 gataacgtagggaaacttacgctaataccgcatacgaccttagggtgaaagcggaggacc

************************************************************

16S_GPE PC73 ttcgggcttcgcgcggatagatgagccgatgtcggattagctagttggcggggtaaaggc

16S_Xa23R1 ttcgggcttcgcgcggatagatgagccgatgtcggattagctagttggcggggtaaaggc

16S_MUS 060 ttcgggcttcgcgcggatagatgagccgatgtcggattagctagttggcggggtaaaggc

16S_GPE 39 ttcgggcttcgcgcggatagatgagccgatgtcggattagctagttggcggggtaaaggc

16S_R1 ttcgggcttcgcgcggatagatgagccgatgtcggattagctagttggcggggtaaaggc

16S_LMG 476 ttcgggcttcgcgcggatagatgagccgatgtcggattagctagttggcggggtaaaggc

16S_NCPPB4393 ttcgggcttcgcgcggatagatgagccgatgtcggattagctagttggcggggtaaaggc

************************************************************

16S_GPE PC73 ccaccaaggcgacgatccgtagctggtctgagaggatgatcagccacactggaactgaga

16S_Xa23R1 ccaccaaggcgacgatccgtagctggtctgagaggatgatcagccacactggaactgaga

16S_MUS 060 ccaccaaggcgacgatccgtagctggtctgagaggatgatcagccacactggaactgaga

16S_GPE 39 ccaccaaggcgacgatccgtagctggtctgagaggatgatcagccacactggaactgaga

16S_R1 ccaccaaggcgacgatccgtagctggtctgagaggatgatcagccacactggaactgaga

16S_LMG 476 ccaccaaggcgacgatccgtagctggtctgagaggatgatcagccacactggaactgaga

16S_NCPPB4393 ccaccaaggcgacgatccgtagctggtctgagaggatgatcagccacactggaactgaga

************************************************************

16S_GPE PC73 cacggtccagactcctacgggaggcagcagtggggaatattggacaatgggcgcaagcct

16S_Xa23R1 cacggtccagactcctacgggaggcagcagtggggaatattggacaatgggcgcaagcct

16S_MUS 060 cacggtccagactcctacgggaggcagcagtggggaatattggacaatgggcgcaagcct

16S_GPE 39 cacggtccagactcctacgggaggcagcagtggggaatattggacaatgggcgcaagcct

16S_R1 cacggtccagactcctacgggaggcagcagtggggaatattggacaatgggcgcaagcct

16S_LMG 476 cacggtccagactcctacgggaggcagcagtggggaatattggacaatgggcgcaagcct

16S_NCPPB4393 cacggtccagactcctacgggaggcagcagtggggaatattggacaatgggcgcaagcct

************************************************************

16S_GPE PC73 gatccagccatgccgcgtgggtgaagaaggccttcgggttgtaaagcccttttgttgggg

16S_Xa23R1 gatccagccatgccgcgtgggtgaagaaggccttcgggttgtaaagcccttttgttgggg

16S_MUS 060 gatccagccatgccgcgtgggtgaagaaggccttcgggttgtaaagcccttttgttggga

16S_GPE 39 gatccagccatgccgcgtgggtgaagaaggccttcgggttgtaaagcccttttgttggga

16S_R1 gatccagccatgccgcgtgggtgaagaaggccttcgggttgtaaagcccttttgttggga

16S_LMG 476 gatccagccatgccgcgtgggtgaagaaggccttcgggttgtaaagcccttttgttggga

16S_NCPPB4393 gatccagccatgccgcgtgggtgaagaaggccttcgggttgtaaagcccttttgttggga

***********************************************************.

16S_GPE PC73 aagaaaagcagtcggttaatacccgattgttctgacggtacccaaagaataagcaccggc

16S_Xa23R1 aagaaaagcagtcggttaatacccgattgttctgacggtacccaaagaataagcaccggc

16S_MUS 060 aagaaaagcagtcggttaatacccgattgttctgacggtacccaaagaataagcaccggc

16S_GPE 39 aagaaaagcagtcggttaatacccggttgttctgacggtacccaaagaataagcaccggc

16S_R1 aagaaaagcagtcggttaatacccgattgttctgacggtacccaaagaataagcaccggc

16S_LMG 476 aagaaaagcagtcggttaatacccgattgttctgacggtacccaaagaataagcaccggc

16S_NCPPB4393 aagaaaagcagtcggttaatacccgattgttctgacggtacccaaagaataagcaccggc

*************************.**********************************

16S_GPE PC73 taacttcgtgccagcagccgcggtaatacgaagggtgcaagcgttactcggaattactgg

16S_Xa23R1 taacttcgtgccagcagccgcggtaatacgaagggtgcaagcgttactcggaattactgg

16S_MUS 060 taacttcgtgccagcagccgcggtaatacgaagggtgcaagcgttactcggaattactgg

16S_GPE 39 taacttcgtgccagcagccgcggtaatacgaagggtgcaagcgttactcggaattactgg

16S_R1 taacttcgtgccagcagccgcggtaatacgaagggtgcaagcgttactcggaattactgg

16S_LMG 476 taacttcgtgccagcagccgcggtaatacgaagggtgcaagcgttactcggaattactgg

16S_NCPPB4393 taacttcgtgccagcagccgcggtaatacgaagggtgcaagcgttactcggaattactgg

************************************************************

16S_GPE PC73 gcgtaaagcgtgcgtaggtggttgtttaagtccgttgtgaaagccctgggctcaacctgg

16S_Xa23R1 gcgtaaagcgtgcgtaggtggttgtttaagtccgttgtgaaagccctgggctcaacctgg

16S_MUS 060 gcgtaaagcgtgcgtaggtggttgtttaagtccgttgtgaaagccctgggctcaacctgg

16S_GPE 39 gcgtaaagcgtgcgtaggtggttgtttaagtccgttgtgaaagccctgggctcaacctgg

16S_R1 gcgtaaagcgtgcgtaggtggttgtttaagtccgttgtgaaagccctgggctcaacctgg

16S_LMG 476 gcgtaaagcgtgcgtaggtggttgtttaagtccgttgtgaaagccctgggctcaacctgg

16S_NCPPB4393 gcgtaaagcgtgcgtaggtggttgtttaagtccgttgtgaaagccctgggctcaacctgg

************************************************************

16S_GPE PC73 gaattgcagtggatactgggcaactagagtgtggtagaggatggcggaattcccggtgta

16S_Xa23R1 gaattgcagtggatactgggcaactagagtgtggtagaggatggcggaattcccggtgta

16S_MUS 060 gaattgcagtggatactgggcaactagagtgtggtagaggatggcggaattcccggtgta

16S_GPE 39 gaattgcagtggatactgggcaactagagtgtggtagaggatggcggaattcccggtgta

16S_R1 gaattgcagtggatactgggcaactagagtgtggtagaggatggcggaattcccggtgta

16S_LMG 476 gaattgcagtggatactgggcaactagagtgtggtagaggatggcggaattcccggtgta

16S_NCPPB4393 gaattgcagtggatactgggcaactagagtgtggtagaggatggcggaattcccggtgta

************************************************************

16S_GPE PC73 gcagtgaaatgcgtagagatcgggaggaacatctgtggcgaaggcggccatctggaccaa

16S_Xa23R1 gcagtgaaatgcgtagagatcgggaggaacatctgtggcgaaggcggccatctggaccaa

16S_MUS 060 gcagtgaaatgcgtagagatcgggaggaacatctgtggcgaaggcggccatctggaccaa

16S_GPE 39 gcagtgaaatgcgtagagatcgggaggaacatctgtggcgaaggcggccatctggaccaa

16S_R1 gcagtgaaatgcgtagagatcgggaggaacatctgtggcgaaggcggccatctggaccaa

16S_LMG 476 gcagtgaaatgcgtagagatcgggaggaacatctgtggcgaaggcggccatctggaccaa

16S_NCPPB4393 gcagtgaaatgcgtagagatcgggaggaacatctgtggcgaaggcggccatctggaccaa

************************************************************

16S_GPE PC73 cactgacactgaggcacgaaagcgtggggagcaaacaggattagataccctggtagtcca

16S_Xa23R1 cactgacactgaggcacgaaagcgtggggagcaaacaggattagataccctggtagtcca

16S_MUS 060 cactgacactgaggcacgaaagcgtggggagcaaacaggattagataccctggtagtcca

16S_GPE 39 cactgacactgaggcacgaaagcgtggggagcaaacaggattagataccctggtagtcca

16S_R1 cactgacactgaggcacgaaagcgtggggagcaaacaggattagataccctggtagtcca

16S_LMG 476 cactgacactgaggcacgaaagcgtggggagcaaacaggattagataccctggtagtcca

16S_NCPPB4393 cactgacactgaggcacgaaagcgtggggagcaaacaggattagataccctggtagtcca

************************************************************

16S_GPE PC73 cgccctaaacgatgcgaactggatgttgggtgcaacttggcacgcagtatcgaagctaac

16S_Xa23R1 cgccctaaacgatgcgaactggatgttgggtgcaacttggcacgcagtatcgaagctaac

16S_MUS 060 cgccctaaacgatgcgaactggatgttgggtgcaacttggcacgcagtatcgaagctaac

16S_GPE 39 cgccctaaacgatgcgaactggatgttgggtgcaacttggcacgcagtatcgaagctaac

16S_R1 cgccctaaacgatgcgaactggatgttgggtgcaacttggcacgcagtatcgaagctaac

16S_LMG 476 cgccctaaacgatgcgaactggatgttgggtgcaacttggcacgcagtatcgaagctaac

16S_NCPPB4393 cgccctaaacgatgcgaactggatgttgggtgcaacttggcacgcagtatcgaagctaac

************************************************************

16S_GPE PC73 gcgttaagttcgccgcctggggagtacggtcgcaagactgaaactcaaaggaattgacgg

16S_Xa23R1 gcgttaagttcgccgcctggggagtacggtcgcaagactgaaactcaaaggaattgacgg

16S_MUS 060 gcgttaagttcgccgcctggggagtacggtcgcaagactgaaactcaaaggaattgacgg

16S_GPE 39 gcgttaagttcgccgcctggggagtacggtcgcaagactgaaactcaaaggaattgacgg

16S_R1 gcgttaagttcgccgcctggggagtacggtcgcaagactgaaactcaaaggaattgacgg

16S_LMG 476 gcgttaagttcgccgcctggggagtacggtcgcaagactgaaactcaaaggaattgacgg

16S_NCPPB4393 gcgttaagttcgccgcctggggagtacggtcgcaagactgaaactcaaaggaattgacgg

************************************************************

16S_GPE PC73 gggcccgcacaagcggtggagtatgtggtttaattcgatgcaacgcgaagaaccttacct

16S_Xa23R1 gggcccgcacaagcggtggagtatgtggtttaattcgatgcaacgcgaagaaccttacct

16S_MUS 060 gggcccgcacaagcggtggagtatgtggtttaattcgatgcaacgcgaagaaccttacct

16S_GPE 39 gggcccgcacaagcggtggagtatgtggtttaattcgatgcaacgcgaagaaccttacct

16S_R1 gggcccgcacaagcggtggagtatgtggtttaattcgatgcaacgcgaagaaccttacct

16S_LMG 476 gggcccgcacaagcggtggagtatgtggtttaattcgatgcaacgcgaagaaccttacct

16S_NCPPB4393 gggcccgcacaagcggtggagtatgtggtttaattcgatgcaacgcgaagaaccttacct

************************************************************

16S_GPE PC73 ggtcttgacatccacggaactttccagagatggattggtgccttcgggaaccgtgagaca

16S_Xa23R1 ggtcttgacatccacggaactttccagagatggattggtgccttcgggaaccgtgagaca

16S_MUS 060 ggtcttgacatccacggaactttccagagatggattggtgccttcgggaaccgtgagaca

16S_GPE 39 ggtcttgacatccacggaactttccagagatggattggtgccttcgggaaccgtgagaca

16S_R1 ggtcttgacatccacggaactttccagagatggattggtgccttcgggaaccgtgagaca

16S_LMG 476 ggtcttgacatccacggaactttccagagatggattggtgccttcgggaaccgtgagaca

16S_NCPPB4393 ggtcttgacatccacggaactttccagagatggattggtgccttcgggaaccgtgagaca

************************************************************

16S_GPE PC73 ggtgctgcatggctgtcgtcagctcgtgtcgtgagatgttgggttaagtcccgcaacgag

16S_Xa23R1 ggtgctgcatggctgtcgtcagctcgtgtcgtgagatgttgggttaagtcccgcaacgag

16S_MUS 060 ggtgctgcatggctgtcgtcagctcgtgtcgtgagatgttgggttaagtcccgcaacgag

16S_GPE 39 ggtgctgcatggctgtcgtcagctcgtgtcgtgagatgttgggttaagtcccgcaacgag

16S_R1 ggtgctgcatggctgtcgtcagctcgtgtcgtgagatgttgggttaagtcccgcaacgag

16S_LMG 476 ggtgctgcatggctgtcgtcagctcgtgtcgtgagatgttgggttaagtcccgcaacgag

16S_NCPPB4393 ggtgctgcatggctgtcgtcagctcgtgtcgtgagatgttgggttaagtcccgcaacgag

************************************************************

16S_GPE PC73 cgcaacccttgtccttagttgccagcacgtaatggtgggaactctaaggagaccgccggt

16S_Xa23R1 cgcaacccttgtccttagttgccagcacgtaatggtgggaactctaaggagaccgccggt

16S_MUS 060 cgcaacccttgtccttagttgccagcacgtcatggtgggaactctaaggagaccgccggt

16S_GPE 39 cgcaacccttgtccttagttgccagcacgtcatggtgggaactctaaggagaccgccggt

16S_R1 cgcaacccttgtccttagttgccagcacgtaatggtgggaactctaaggagaccgccggt

16S_LMG 476 cgcaacccttgtccttagttgccagcacgtaatggtgggaactctaaggagaccgccggt

16S_NCPPB4393 cgcaacccttgtccttagttgccagcacgtaatggtgggaactctaaggagaccgccggt

****************************** *****************************

16S_GPE PC73 gacaaaccggaggaaggtggggatgacgtcaagtcatcatggcccttacgaccagggcta

16S_Xa23R1 gacaaaccggaggaaggtggggatgacgtcaagtcatcatggcccttacgaccagggcta

16S_MUS 060 gacaaaccggaggaaggtggggatgacgtcaagtcatcatggcccttacgaccagggcta

16S_GPE 39 gacaaaccggaggaaggtggggatgacgtcaagtcatcatggcccttacgaccagggcta

16S_R1 gacaaaccggaggaaggtggggatgacgtcaagtcatcatggcccttacgaccagggcta

16S_LMG 476 gacaaaccggaggaaggtggggatgacgtcaagtcatcatggcccttacgaccagggcta

16S_NCPPB4393 gacaaaccggaggaaggtggggatgacgtcaagtcatcatggcccttacgaccagggcta

************************************************************

16S_GPE PC73 cacacgtactacaatggtaaggacagagggctgcaaactcgcgagagtgagccaatccca

16S_Xa23R1 cacacgtactacaatggtaaggacagagggctgcaaactcgcgagagtgagccaatccca

16S_MUS 060 cacacgtactacaatggtaaggacagagggctgcaaactcgcgagagtgagccaatccca

16S_GPE 39 cacacgtactacaatggtaaggacagagggctgcaaactcgcgagagtgagccaatccca

16S_R1 cacacgtactacaatggtagggacagagggctgcaagccggcgacggtaagccaatccca

16S_LMG 476 cacacgtactacaatggtagggacagagggctgcaagccggcgacggtgagccaatccca

16S_NCPPB4393 cacacgtactacaatggtagggacagagggctgcaagccggcgacggtgagccaatccca

*******************.****************.*. **** .**.***********

16S_GPE PC73 gaaaccttatctcagtccggattggagtctgcaactcgactccatgaagtcggaatcgct

16S_Xa23R1 gaaaccttatctcagtccggattggagtctgcaactcgactccatgaagtcggaatcgct

16S_MUS 060 gaaaccttatctcagtccggattggagtctgcaactcgactccatgaagtcggaatcgct

16S_GPE 39 gaaaccttatctcagtccggattggagtctgcaactcgactccatgaagtcggaatcgct

16S_R1 gaaaccctatctcagtccggattggagtctgcaactcgactccatgaagtcggaatcgct

16S_LMG 476 gaaaccctatctcagtccggattggagtctgcaactcgactccatgaagtcggaatcgct

16S_NCPPB4393 gaaaccctatctcagtccggattggagtctgcaactcgactccatgaagtcggaatcgct

******.*****************************************************

16S_GPE PC73 agtaatcgcagatcagcattgctgcggtgaatacgttcccgggccttgtacacaccgccc

16S_Xa23R1 agtaatcgcagatcagcattgctgcggtgaatacgttcccgggccttgtacacaccgccc

16S_MUS 060 agtaatcgcagatcagcattgctgcggtgaatacgttcccgggccttgtacacaccgccc

16S_GPE 39 agtaatcgcagatcagcattgctgcggtgaatacgttcccgggccttgtacacaccgccc

16S_R1 agtaatcgcagatcagcattgctgcggtgaatacgttcccgggccttgtacacaccgccc

16S_LMG 476 agtaatcgcagatcagcattgctgcggtgaatacgttcccgggccttgtacacaccgccc

16S_NCPPB4393 agtaatcgcagatcagcattgctgcggtgaatacgttcccgggccttgtacacaccgccc

************************************************************

16S_GPE PC73 gtcacaccatgggagtttgttgcaccagaagcaggtagcttaaccttcgggagggcgctt

16S_Xa23R1 gtcacaccatgggagtttgttgcaccagaagcaggtagcttaaccttcgggagggcgctt

16S_MUS 060 gtcacaccatgggagtttgttgcaccagaagcaggtagcttaaccttcgggagggcgctt

16S_GPE 39 gtcacaccatgggagtttgttgcaccagaagcaggtagcttaaccttcgggagggcgctt

16S_R1 gtcacaccatgggagtttgttgcaccagaagcaggtagcttaaccttcgggagggcgctt

16S_LMG 476 gtcacaccatgggagtttgttgcaccagaagcaggtagcttaaccttcgggagggcgctt

16S_NCPPB4393 gtcacaccatgggagtttgttgcaccagaagcaggtagcttaaccttcgggagggcgctt

************************************************************

16S_GPE PC73 gccacggtgtggccgatgactggggtgaagtcgtaacaaggtagccgtatcggaaggtgc

16S_Xa23R1 gccacggtgtggccgatgactggggtgaagtcgtaacaaggtagccgtatcggaaggtgc

16S_MUS 060 gccacggtgtggccgatgactggggtgaagtcgtaacaaggtagccgtatcggaaggtgc

16S_GPE 39 gccacggtgtggccgatgactggggtgaagtcgtaacaaggtagccgtatcggaaggtgc

16S_R1 gccacggtgtggccgatgactggggtgaagtcgtaacaaggtagccgtatcggaaggtgc

16S_LMG 476 gccacggtgtggccgatgactggggtgaagtcgtaacaaggtagccgtatcggaaggtgc

16S_NCPPB4393 gccacggtgtggccgatgactggggtgaagtcgtaacaaggtagccgtatcggaaggtgc

************************************************************

**16S** ribosomal DNA gene sequence **pairwise identity between strains:**

|  | **GPE PC73_16S** | **Xa23R1_16S** | **MUS 060_16S** | **GPE 39_16S** | **R1_16S** | **LMG 476_16S** | **NCPPB4393_16S** |
| --- | --- | --- | --- | --- | --- | --- | --- |
| **GPE PC73_16S** |  | 100.00 | 99.80 | 99.73 | 98.60 | 98.67 | 98.67 |
| **Xa23R1_16S** | 100.00 |  | 99.80 | 99.73 | 98.60 | 98.67 | 98.67 |
| **MUS 060_16S** | 99.80 | 99.80 |  | 99.93 | 98.53 | 98.60 | 98.60 |
| **GPE 39_16S** | 99.73 | 99.73 | 99.93 |  | 98.47 | 98.53 | 98.53 |
| **R1_16S** | 98.60 | 98.60 | 98.53 | 98.47 |  | 99.93 | 99.93 |
| **LMG 476_16S** | 98.67 | 98.67 | 98.60 | 98.53 | 99.93 |  | 100.00 |
| **NCPPB4393_16S** | 98.67 | 98.67 | 98.60 | 98.53 | 99.93 | 100.00 |  |
