# Supplementary material for: Full Genome Sequence Analysis of Two Isolates Reveals a Novel Xanthomonas Species Close to the Sugarcane Pathogen Xanthomonas albilineans
Source: Genes (Basel). 2015 Jul 23;6(3):714–33. doi: 10.3390/genes6030714 (PMC4584326; doi:10.3390/genes6030714)
Supplement: Supplementary File 1 [file genes-06-00714-s001.zip › genes-85642-supplementary/Supplementary File S2_v2-2.docx]

**Supplementary File S2**

**16S–23S rRNA internal transcribed spacer sequence (ITS) alignment and pairwise identity**

**CLUSTAL format alignment by MAFFT (v7.214)**

GPE PC73_ITS ggctggatcacctccttttgagcatgacagcttcatctgtcaggcgtcctcacaagtaac

Xa23R1_ITS ggctggatcacctccttttgagcatgacagcttcatctgtcaggcgtcctcacaagtaac

GPE 39_ITS ggctggatcacctccttttgagcatgacagtttcatctgtcaggcgtcctcacaagtaac

MUS 060_ITS ggctggatcacctccttttgagcatgacagtttcatctgtcaggcgtcctcacaagtaac

NCPPB4393_ITS ggctggatcacctccttttgagcatgacagctccatctgtcaggcgtcctcacaagtaac

LMG 476_ITS ggctggatcacctccttttgagcatgacagctccatctgtcaggcgtcctcacaagtaac

R1_ITS ggctggatcacctccttttgagcatgacagcttcatctgtcaggcgtcctcacaagtaac

******************************.*.***************************

GPE PC73_ITS ctgcattcagagagtttcgccatggggcggaacaccccgaaataccggggccatagctca

Xa23R1_ITS ctgcattcagagagtttcgccatggggcggaacaccccgaaataccggggccatagctca

GPE 39_ITS ctgcattcagagagtttcgccaaggggcggaacaccccgaaataccggggccatagctca

MUS 060_ITS ctgcattcagagagtttcgccaaggggcggaacaccccgaaataccggggccatagctca

NCPPB4393_ITS ctgcattcagagagttccgccacagggcggagcaccccgaaataccggggccatagctca

LMG 476_ITS ctgcattcagagagttccgccacagggcggagcaccccgaaataccggggccatagctca

R1_ITS ctgcattcagagagttccgccacagggcggagcaccccgaaataccggggccatagctca

****************.***** .*******.****************************

GPE PC73_ITS gctgggagagcacctgctttgcaagcagggggtcgtcggttcgatcccgactggctccac

Xa23R1_ITS gctgggagagcacctgctttgcaagcagggggtcgtcggttcgatcccgactggctccac

GPE 39_ITS gctgggagagcacctgctttgcaagcagggggtcgtcggttcgatcccgactggctccac

MUS 060_ITS gctgggagagcacctgctttgcaagcagggggtcgtcggttcgatcccgactggctccac

NCPPB4393_ITS gctgggagagcacctgctttgcaagcagggggtcgtcggttcgatcccgactggctccac

LMG 476_ITS gctgggagagcacctgctttgcaagcagggggtcgtcggttcgatcccgactggctccac

R1_ITS gctgggagagcacctgctttgcaagcagggggtcgtcggttcgatcccgactggctccac

************************************************************

GPE PC73_ITS cactgatgacgactttgggtctgtagctcaggtggttagagcgcacccctgataagggtg

Xa23R1_ITS cactgatgacgactttgggtctgtagctcaggtggttagagcgcacccctgataagggtg

GPE 39_ITS cactgaggacgacattgggtctgtagctcaggtggttagagcgcacccctgataagggtg

MUS 060_ITS cactgaggacgacattgggtctgtagctcaggtggttagagcgcacccctgataagggtg

NCPPB4393_ITS cactgacgaagactttgggtctgtagctcaggtggttagagcgcacccctgataagggtg

LMG 476_ITS cactgacgaagactttgggtctgtagctcaggtggttagagcgcacccctgataagggtg

R1_ITS cactgacgaagactttgggtctgtagctcaggtggttagagcgcacccctgataagggtg

****** ** *** **********************************************

GPE PC73_ITS aggtcggtggttcgagtcctcccagacccaccactctgaatgtatcgcacacgaagaatt

Xa23R1_ITS aggtcggtggttcgagtcctcccagacccaccactctgaatgtatcgcacacgaagaatt

GPE 39_ITS aggtcggtggttcgagtcctcccagacccaccactctgaatgtatcgcacacgaagaatt

MUS 060_ITS aggtcggtggttcgagtcctcccagacccaccactctgaatgtatcgcacacgaagaatt

NCPPB4393_ITS aggtcggtggttcgagtcctcccagacccaccactctgaatgtatcgcacactaagaatt

LMG 476_ITS aggtcggtggttcgagtcctcccagacccaccactctgaatgtatcgcacactaagaatt

R1_ITS aggtcggtggttcgagtcctcccagacccaccactctgaatgtatcgcacactaagaatt

**************************************************** *******

GPE PC73_ITS tgaatgattcggcgctgaggccgggtcg-tgttctttaataattagtgatgtagcgaacg

Xa23R1_ITS tgaatgattcggcgctgaggccgggtcg-tgttctttaataattagtgatgtagcgaacg

GPE 39_ITS tgaatgattcggcgctgaggccgggtca-tgttctttaataattagtgatgtagcgaacg

MUS 060_ITS tgaatgattcggcgctgaggccgggtca-tgttctttaataattagtgatgtagcgaacg

NCPPB4393_ITS tagatg-ctcggcgttgaggccgtagcgttgttctttaataatttgtgatgtagcgagcg

LMG 476_ITS tagatg-ctcggcgttgaggccgtagcgttgttctttaataatttgtgatgtagcgagcg

R1_ITS tagatg-ctcggcgttgaggccgtagcgttgttctttaataatttgtgatgtagcgagcg

*..*** .******.******** . *. *************** ************.**

GPE PC73_ITS tttgagaacaatactctcgacgtgtcgttgtggctaaggcggggacttcgagtcccta-a

Xa23R1_ITS tttgagaacaatactctcgacgtgtcgttgtggctaaggcggggacttcgagtcccta-a

GPE 39_ITS tttgagaacaatactctcgacgtgtcgttgtggctaaggcggggacttcgagtcccta-a

MUS 060_ITS tttgagaacaatactctcgacgtgtcgttgtggctaaggcggggacttcgagtcccta-a

NCPPB4393_ITS tttgagaacaaacttctcgacgtgtcgttgtggctaaggcggggacctcgagtccctaga

LMG 476_ITS tttgagaacaaacttctcgacgtgtcgttgtggctaaggcggggacctcgagtccctaga

R1_ITS tttgagaacaaacttctcgacgtgtcgttgtggctaaggcggggacctcgagtccctaga

*********** .********************************.*********** *

GPE PC73_ITS aattgagtcgttatagttcgcgtccgggctttgtacccctgggctgaatatgaccttgag

Xa23R1_ITS aattgagtcgttatagttcgcgtccgggctttgtacccctggactggatatgaccttgag

GPE 39_ITS aattgagtcgttatagttcgcgtccaggctttgtacccctggactgaagatgaccttgag

MUS 060_ITS aattgagtcgttatagttcgcgtccaggctttgtacccctggactgaagatgaccttgag

NCPPB4393_ITS aattgagtcgttatagttcgcgtccaggctttgtacccctggactgaatatgaccttgag

LMG 476_ITS aattgagtcgttatagttcgcgtccaggctttgtacccctggactgaatatgaccttgag

R1_ITS aattgagtcgttatagttcgcgtccaggctttgtacccctggactgaatatgaccttgag

*************************.****************.***.* ***********

GPE PC73_ITS gcaacttgaggttatat

Xa23R1_ITS gcaacttgaggttatat

GPE 39_ITS gcaacttgaggttatat

MUS 060_ITS gcaacttgaggttatat

NCPPB4393_ITS gcaacttgaggttatat

LMG 476_ITS gcaacttgaggttatat

R1_ITS gcaacttgaggttatat

*****************

**16S-23S ITS pairwise identity between strains:**

|  | GPE PC73_ITS | Xa23R1_ITS | GPE 39_ITS | MUS 060_ITS | NCPPB4393_ITS | LMG 476_ITS | R1_ITS |
| --- | --- | --- | --- | --- | --- | --- | --- |
| GPE PC73_ITS |  | 99.60 | 98.38 | 98.38 | 94.77 | 94.77 | 94.97 |
| Xa23R1_ITS | 99.60 |  | 98.38 | 98.38 | 94.77 | 94.77 | 94.97 |
| GPE 39_ITS | 98.38 | 98.38 |  | 100.00 | 94.37 | 94.37 | 94.57 |
| MUS 060_ITS | 98.38 | 98.38 | 100.00 |  | 94.37 | 94.37 | 94.57 |
| NCPPB4393_ITS | 94.77 | 94.77 | 94.37 | 94.37 |  | 100.00 | 99.80 |
| LMG 476_ITS | 94.77 | 94.77 | 94.37 | 94.37 | 100.00 |  | 99.80 |
| R1_ITS | 94.97 | 94.97 | 94.57 | 94.57 | 99.80 | 99.80 |  |
