# Supplementary material for: Full Genome Sequence Analysis of Two Isolates Reveals a Novel Xanthomonas Species Close to the Sugarcane Pathogen Xanthomonas albilineans
Source: Genes (Basel). 2015 Jul 23;6(3):714–33. doi: 10.3390/genes6030714 (PMC4584326; doi:10.3390/genes6030714)
Supplement: Supplementary File 1 [file genes-06-00714-s001.zip › genes-85642-supplementary/Supplementary File S5_v2.docx]

**Supplementary File S5**

Alignment of concatenated nucleic acid sequences used for MLSA phylogeny

1

GPE PC73 ATGACCGACG AACAGAACAC CCACGCAA-- -ACAACGGCA ATTACGACGC GAATAGCATC ACGGCCCTGG

Xa23R1 ATGACCGACG AACAGAACAC CCACGCAA-- -ACAACGGCA ATTACGACGC GAATAGCATC ACCGCCCTGG

GPE 39 ATGACCGACG AACAGAACAC CCCGGCAA-- -ACAACGGCA ATTACGACGC GAATAGCATC ACTGCCCTGG

MUS 060 ATGACCGACG AACAGAACAC CCCGGCAA-- -ACAACGGCA ATTACGACGC GAATAGCATC ACTGCCCTGG

LMG 476 ATGACCGACG AACAGAACAC CCCGCCCA-- -GCAACGGCA ACTACGACGC CAACAGCATC ACCGCCCTGG

NCPPB4393 ATGACCGACG AACAGAACAC CCCGCCCA-- -GCAACGGCA ACTACGACGC CAACAGCATC ACCGCCCTGG

R1 ATGACCGACG AACAGAACAC CCCGCCCA-- -GCAACGGCA ACTACGACGC CAACAGCATC ACCGCCCTGG

XCV85-10 ATGACCGACG AACAAAACAT CCCGCCAACA CCCAACGGCA CCTACGACTC CAGCAAGATC ACCGTGCTGC

71

GPE PC73 AAGGGTTGGA AGCGGTCCGC AAGCGTCCAG GGATGTACAT CGGTGACGTC CATGACGGCA CTGGCCTGCA

Xa23R1 AAGGGCTGGA AGCGGTCCGC AAGCGTCCAG GGATGTACAT CGGTGACGTC CATGACGGCA CTGGCCTGCA

GPE 39 AAGGCCTGGA AGCAGTGCGC AAGCGTCCAG GGATGTACAT CGGTGACGTC CATGACGGCA CTGGCCTGCA

MUS 060 AAGGCCTGGA AGCAGTGCGC AAGCGTCCAG GGATGTACAT CGGTGACGTC CATGACGGCA CTGGCCTGCA

LMG 476 AAGGGCTGGA GGCGGTCCGC AAGCGGCCCG GCATGTACAT CGGCGACGTC CATGACGGCA CCGGCCTGCA

NCPPB4393 AAGGGCTGGA GGCGGTCCGC AAGCGGCCCG GCATGTACAT CGGCGACGTC CATGACGGCA CCGGCCTGCA

R1 AAGGCCTGGA GGCGGTCCGC AAGCGGCCCG GCATGTACAT CGGCGACGTC CATGACGGCA CCGGTCTGCA

XCV85-10 GTGGCCTGGA GGCCGTCCGC AAGCGCCCCG GCATGTATAT CGGCGACGTC CATGACGGCA CCGGCCTGCA

141

GPE PC73 TCACATGGTG TTCGAGGTTG TCGATAACTC CATCGACGAG GCGCTGGCCG GTCATGCGGA CCATGTCGCG

Xa23R1 TCACATGGTG TTCGAGGTTG TCGATAACTC CATCGACGAG GCGCTGGCCG GTCATGCGGA CCATGTCGCG

GPE 39 TCACATGGTG TTCGAGGTGG TCGATAACTC CATCGACGAG GCTTTGGCCG GTCATGCGGA CCATGTCGCG

MUS 060 TCACATGGTG TTCGAGGTGG TCGATAACTC CATCGACGAG GCTTTGGCCG GTCATGCGGA CCATGTCGCG

LMG 476 TCACATGGTG TTCGAGGTCG TCGACAACTC CATCGACGAA GCGCTGGCCG GGCATGCCGA CCACGTCGCG

NCPPB4393 TCACATGGTG TTCGAGGTCG TCGACAACTC CATCGACGAA GCGCTGGCCG GGCATGCCGA CCACGTCGCG

R1 TCACATGGTG TTCGAGGTCG TCGACAACTC CATCGACGAA GCCCTGGCCG GGCATGCCGA CCACGTCGCG

XCV85-10 TCACATGGTG TTCGAGGTGG TCGACAACTC GGTCGACGAA GCCCTGGCCG GGCATGCGGA CGACATCGTG

211

GPE PC73 GTTACGATCC ATGCCGATGG TTCGGTGTCG GTATCGGATA ACGGACGTGG CATTCCGGTC GGCAAGCATG

Xa23R1 GTTACGATCC ATGCTGATGG TTCGGTGTCG GTATCGGATA ACGGACGTGG CATTCCGGTC GGCAAGCATG

GPE 39 GTCATGATCC ATGCCGATGG TTCGGTGTCT GTGTCCGATA ACGGCCGTGG CATTCCGGTC GGCAAGCATG

MUS 060 GTCATGATCC ATGCCGATGG TTCAGTGTCT GTGTCCGATA ACGGCCGTGG CATTCCGGTC GGCAAGCATG

LMG 476 GTGACGATCC ACGCCGATGG CTCGGTCTCA GTGTCGGACA ACGGCCGCGG TATCCCGGTC GGCAAGCACG

NCPPB4393 GTGACGATCC ACGCCGATGG CTCGGTCTCA GTGTCGGACA ACGGCCGCGG TATCCCGGTC GGCAAGCACG

R1 GTGACGATCC ACGCCGATGG TTCGGTCTCG GTGTCGGACA ATGGCCGCGG CATCCCGGTC GGCAAGCACG

XCV85-10 GTGAAGATCC TGGCCGATGG CTCGGTGGCG GTCTCCGACA ACGGGCGCGG CGTACCGGTC GACATCCACA

281

GPE PC73 CGCAGATGAG CAAGAAGCTC GATCGCGAGG TCTCGGCGGC CGAAGTCGTG ATGACCGTCC TGCATGCAGG

Xa23R1 CGCAGATGAG CAAGAAGCTC GATCGCGAGG TCTCGGCGGC CGAAGTCGTG ATGACCGTGC TGCATGCAGG

GPE 39 CGCAGATGAG CAAGAAGCTT GATCGCGAAG TTTCGGCAGC AGAAGTCGTG ATGACCGTCC TGCATGCAGG

MUS 060 CGCAGATGAG CAAGAAGCTT GATCGCGAGG TCTCGGCGGC AGAAGTCGTG ATGACCGTCC TGCATGCAGG

LMG 476 CGCAGATGAG CGCCAAGCTC GGCCGCGAGG TCTCCGCCGC CGAAGTGGTG ATGACCGTGC TGCACGCCGG

NCPPB4393 CGCAGATGAG CGCCAAGCTC GGCCGCGAGG TCTCCGCCGC CGAAGTGGTG ATGACCGTGC TGCACGCCGG

R1 CGCAGATGAG CGCCAAGCTC GGCCGTGAGG TCTCCGCCGC CGAAGTGGTG ATGACCGTCC TGCACGCCGG

XCV85-10 AGGAAGAAGG C--------- ---------G TCTCGGCGGC CGAGGTGATC CTCACCGTGC TGCACGCCGG

351

GPE PC73 CGGTAAGTTC GACGACAACA GCTACAAGGT TTCTGGTGGC CTGCATGGCG TTGGTGTCAG TGTGGTCAAC

Xa23R1 CGGTAAGTTC GACGACAACA GCTACAAGGT CTCTGGTGGC CTGCATGGCG TTGGTGTCAG TGTGGTCAAC

GPE 39 TGGTAAGTTC GACGACAACA GCTACAAGGT TTCTGGTGGC CTGCATGGCG TCGGCGTCAG CGTGGTCAAC

MUS 060 CGGAAAGTTC GACGACAACA GCTACAAGGT TTCTGGTGGC CTGCATGGCG TCGGCGTCAG CGTGGTCAAC

LMG 476 CGGCAAGTTC GACGACAACA GCTACAAGGT GTCCGGCGGC CTGCATGGCG TGGGCGTCAG CGTGGTCAAC

NCPPB4393 CGGCAAGTTC GACGACAACA GCTACAAGGT GTCCGGCGGC CTGCATGGCG TGGGCGTCAG CGTGGTCAAC

R1 CGGCAAGTTC GACGACAACA GCTACAAGGT GTCCGGCGGC CTGCACGGCG TGGGCGTCAG CGTGGTCAAC

XCV85-10 CGGCAAGTTC GACGACAACA GCTACAAGGT CTCCGGCGGC CTGCATGGCG TAGGCGTCTC GGTGGTCAAC

421

GPE PC73 GCGCTGTCGG AAAAGCTGCT GCTGGATATT TTCCAGGGCG GCTTCCATTA CCAGCAGGAA TATAGCCACG

Xa23R1 GCGCTGTCGG AAAAGCTGCT GCTGGATATT TTCCAGGGCG GCTTCCATTA CCAGCAGGAA TATAGCCACG

GPE 39 GCGCTATCGG AAAAGCTGAT GCTGGATATT TTCCAGGGTG GCTTCCATTA CCAGCAGGAA TACAGCCACG

MUS 060 GCGTTGTCGG AAAAGTTGCT GCTGGATATT TTCCAGGGTG GCTTCCATTA CCAGCAGGAA TACAGCCACG

LMG 476 GCGCTGTCGG AGAAGCTGCT GCTGGACGTG TTCCAGGGCG GTTTCCACTA CCAGCAGGAA TTCAGCAATG

NCPPB4393 GCGCTGTCGG AGAAGCTGCT GCTGGACGTG TTCCAGGGCG GTTTCCACTA CCAGCAGGAA TTCAGCAATG

R1 GCGCTGTCGG AGAAGCTGCT GCTGGACGTG TTCCAGGGCG GCTCGCACTA CCAGCAGGAA TTCAGCAACG

XCV85-10 GCATTGTCCG AGCACTTGTG GCTGGATATC TGGCGCGACG GCTTCCACTA CCAGCAGGAA TACGCGCTGG

491

GPE PC73 GCGCCGCCTT GGCGCCGCTG AAGCAGCTGG AAACCAGCGC CAAGCGTGGT ACCACGTTAC GCTTCTGGCC

Xa23R1 GCGCCGCCTT GGCGCCGCTG AAGCAACTGG AAACCAGCGC CAAGCGTGGT ACCACGTTAC GCTTCTGGCC

GPE 39 GTGCCGCCTT GGCACCGCTG AAGCAGTTGG AAACCAGCAC TAAGCGCGGC ACCACACTCC GCTTCTGGCC

MUS 060 GTGCCGCCTT GGCGCCGCTG AAGCAATTGG AAACCAGCAC CAAGCGCGGC ACCACACTCC GCTTCTGGCC

LMG 476 GCGCAGCCGT CGCGCCGCTG GTGAAGCTGG GCGAGACCAC CAAGCGCGGC ACCACCGTGC GCTTCTGGCC

NCPPB4393 GCGCAGCCGT CGCGCCGCTG GTGAAGCTGG GCGAGACCAC CAAGCGCGGC ACCACCGTGC GCTTCTGGCC

R1 GCGCGGCCGT CGCGCCGCTG GTCAAGCTCG GCGAGACCAC CCAGCGCGGT ACCACCGTAC GCTTCTGGCC

XCV85-10 GCGAACCGCA GTACCCGCTC AAGCAGCTGG AAGCCTCGAC CAAGCGCGGC ACCACGCTGC GCTTCAAGCC

561

GPE PC73 GTCGGTGAAG GCGTTCAATA ACAACGTCGA GTTCCACTAC GACATCCTGG CCCGCCGCCT GCGTGAGCTG

Xa23R1 GTCGGTGAAG GCGTTCAATA ACAACGTCGA GTTCCACTAC GACATCCTGG CCCGCCGCCT GCGTGAGCTG

GPE 39 GTCGGTGAAG GCGTTCAATA ACAACGTCGA GTTCCACTAC GACATCCTGG CTCGACGCCT GCGTGAGCTG

MUS 060 GTCGGTGAAG GCGTTCAATA ACAATGTCGA GTTCCACTAC GACATCCTGG CACGGCGCCT GCGTGAGCTG

LMG 476 GTCGGTGGTG GCCTTCCACA ACAACGTGGA GTTCCACTAC GACATCCTGG CGCGCCGCCT GCGCGAGCTG

NCPPB4393 GTCGGTGGTG GCCTTCCACA ACAACGTGGA GTTCCACTAC GACATCCTGG CGCGCCGCCT GCGCGAGCTG

R1 GTCGGTGGTG GCCTTCCACA ACAACGTGGA GTTCCACTAC GACATCCTGG CCCGGCGCCT GCGCGAGCTG

XCV85-10 GTCCGTGGCC ATCTTC---A GCGATGTCGA GTTCCATTAC GACATCCTGG CGCGGCGCCT GCGCGAGCTG

631

GPE PC73 TCCTTCCTCA ACTCCGGGGT CAAGATCGTC CTAGCCGACG AACGTGGGGA AGGCCGTCGC GACGATTTCC

Xa23R1 TCCTTCCTCA ACTCCGGGGT CAAGATCGTC CTAGCCGACG AACGTGGGGA AGGCCGTCGC GACGATTTCC

GPE 39 TCCTTCCTCA ACTCCGGGGT CAAGATCGTG CTCGCCGACG AGCGCGGTGA AGGCCGTCGC GACGATTTCC

MUS 060 TCCTTCCTCA ACTCCGGGGT CAAGATCGTG CTCGCCGACG AGCGCGGCGA AGGCCGTCGC GACGATTTCC

LMG 476 TCCTTCCTCA ATTCCGGAGT CAAGATCGTG CTCGCCGACG AGCGCGGCGA GGGCCGGCGC GACGACTTCC

NCPPB4393 TCCTTCCTCA ATTCCGGAGT CAAGATCGTG CTCGCCGACG AGCGCGGCGA GGGCCGGCGC GACGACTTCC

R1 TCCTTCCTCA ATTCCGGGGT CAAGATCGTG CTCGCCGACG AGCGCGGCGA GGGGCGCCGC GACGACTTCC

XCV85-10 TCCTTCCTCA ATTCCGGCGT CAAGATCACC TTGATCGACG AGCGCGGCGA AGGCCGTCGC GACGATTTCC

701

GPE PC73 ACTACGAAGG CGGCATCCGC AGCTTCGTCG AGCACTTGGC GCACCTGAAA ACCCCACTGC ATCCGAACGT

Xa23R1 ACTACGAAGG CGGCATCCGC AGCTTCGTCG AGCACTTGGC GCACCTGAAA ACCCCACTGC ATCCGAACGT

GPE 39 ATTACGAAGG TGGCATCCGT AGCTTCGTCG AGCACTTGGC GCACCTGAAA ACCCCGCTGC ATCCGAACGT

MUS 060 ACTACGAAGG TGGCATCCGC AGCTTCGTCG AGCACTTGGC GCACCTGAAA ACCCCGCTGC ATCCGAACGT

LMG 476 ACTACGAGGG CGGCATCCGC AGCTTCGTCG AGCACCTGGC GCAGCTGAAG ACCCCGCTGC ATCCGAACGT

NCPPB4393 ACTACGAGGG CGGCATCCGC AGCTTCGTCG AGCACCTGGC GCAGCTGAAG ACCCCGCTGC ATCCGAACGT

R1 ACTACGAGGG CGGCATCCGC AGCTTCGTCG AGCACCTCGC GCAGCTGAAG ACCCCGCTGC ACCCGAACGT

XCV85-10 ATTACGAAGG CGGCATCCGC AGCTTCGTGG AGCATCTGGC GCAGTTGAAG TCGCCGCTGC ACCCGAATGT

771

GPE PC73 GATTTCGGTG AGCGGCGAAC ATAACGGCAT CACCGTGGAA GTGGCGCTGC AGTGGACCGA CTCCTATCAG

Xa23R1 GATTTCGGTG AGCGGCGAAC ATAACGGCAT CACCGTGGAA GTGGCGCTGC AGTGGACCGA CTCCTATCAG

GPE 39 GATTTCGGTG AGCGGTGAAC ATAACGGCAT CACCGTGGAA GTGGCGCTGC AATGGACCGA CTCCTACCAG

MUS 060 GATTTCGGTG AGCGGTGAAC ATAACGGCAT CACCGTGGAA GTGGCGCTGC AATGGACCGA CTCCTATCAG

LMG 476 GATCTCGGTC ACCGGCGAGC ACAACGGCAT CACCGTGGAC GTGGCGCTGC AGTGGACCGA CTCCTACCAG

NCPPB4393 GATCTCGGTC ACCGGCGAGC ACAACGGCAT CACCGTGGAC GTGGCGCTGC AGTGGACCGA CTCCTACCAG

R1 GATCTCTGTC ACCGGCGAGC ACAACGGCAT CACCGTGGAC GTGGCGCTGC AGTGGACCGA CTCCTACCAG

XCV85-10 GATCTCGGTG ACCGGCGAGC ACAACGGCAT CATGGTGGAC GTGGCCCTGC AATGGACCGA CGCCTACCAG

841

GPE PC73 GAGACGATGT ACTGTTTCAC CAACAATATC CCGCAAAAAG ACGGTGGCAC CCACCTGGCA GGTTTCCGCG

Xa23R1 GAGACGATGT ACTGTTTCAC CAACAATATC CCGCAAAAAG ACGGTGGCAC CCATCTGGCA GGTTTCCGCG

GPE 39 GAGACGATGT ACTGCTTCAC CAACAACATC CCGCAAAAAG ACGGTGGCAC TCACCTGGCG GGTTTCCGCG

MUS 060 GAGACGATGT ACTGTTTTAC CAACAACATC CCGCAAAAAG ACGGTGGTAC TCACCTGGCG GGTTTCCGCG

LMG 476 GAGACGATGT ACTGCTTCAC CAACAACATC CCGCAGAAGG ACGGCGGCAC CCACCTGGCC GGCTTCCGCG

NCPPB4393 GAGACGATGT ACTGCTTCAC CAACAACATC CCGCAGAAGG ACGGCGGCAC CCACCTGGCC GGCTTCCGCG

R1 GAGACGATGT ACTGCTTCAC CAACAACATC CCGCAGAAGG ACGGCGGCAC CCACCTGGCC GGCTTCCGCG

XCV85-10 GAAACCATGT ACTGCTTCAC CAACAATATT CCGCAGAAGG ACGGCGGCAC CCACCTGGCC GGTTTCCGTG

911

GPE PC73 GTGCCCTGAC CCGCGTGCTC AACAACTACA TCGAGCAGAA TGGCATTGCC AAGCAGGCCA AGATCAACCT

Xa23R1 GTGCCCTGAC CCGCGTGCTC AACAACTACA TCGAGCAGAA TGGCATTGCC AAGCAGGCCA AGATCAACCT

GPE 39 GTGCCTTGAC CCGCGTGCTC AACAACTACA TCGAGCAAAA TGGCATCGCC AAGCAGGCCA AGATCAATCT

MUS 060 GTGCCTTGAC GCGCGTGCTC AACAACTACA TCGAGCAGAA TGGTATCGCC AAGCAGGCCA AGATCAATCT

LMG 476 GCGCGCTGAC CCGCGTGCTC AACACCTACA TCGAGCAGAA CGGCATCGCC AAGCAGGCCA AGATCAACCT

NCPPB4393 GCGCGCTGAC CCGCGTGCTC AACACCTACA TCGAGCAGAA CGGCATCGCC AAGCAGGCCA AGATCAACCT

R1 GCGCGCTGAC CCGCGTGCTC AATACCTACA TCGAGCAGAA CGGCATCGCC AAGCAGGCCA AGATCAATCT

XCV85-10 CGGCGCTGAC GCGCGTGCTC AGCACCTACA TCGAACAGAA CGGCATCGCC AAGCAGGCCA AGGTCGCGCT

981

GPE PC73 GACCGGCGAT GACATGCGCG AAGGCATGAT TGCGGTGCTC TCGGTGAAGG TGCCGGACCC GAGTTTTTCC

Xa23R1 GACCGGCGAT GACATGCGCG AAGGCATGAT TGCGGTGCTC TCGGTGAAGG TGCCTGACCC GAGTTTTTCC

GPE 39 GACTGGCGAT GACATGCGCG AAGGCATGAT CGCGGTGCTC TCGGTGAAGG TCCCAGACCC GAGTTTTTCC

MUS 060 GACTGGCGAT GACATGCGCG AAGGCATGAT CGCGGTGCTC TCGGTGAAGG TTCCAGACCC GAGTTTTTCC

LMG 476 GACCGGCGAC GACATGCGCG AAGGCATGAT CGCGGTGCTG TCGGTGAAAG TGCCGGACCC GAGCTTCTCC

NCPPB4393 GACCGGCGAC GACATGCGCG AAGGCATGAT CGCGGTGCTG TCGGTGAAAG TGCCGGACCC GAGCTTCTCC

R1 GACCGGCGAC GACATGCGCG AAGGCATGAT CGCGGTGCTG TCGGTGAAGG TGCCGGACCC CAGCTTCTCC

XCV85-10 GACCGGCGAT GACATGCGCG AAGGCATGAT CGCGGTGCTT TCGGTCAAGG TGCCCGACCC CAGCTTCTCT

1051

GPE PC73 AGCCAGACCA AGGAAAAGCT GGTCAGTTCC GACGTGCGCC CGGCGGTGGA AAATGCGTTC GGTGCCCGTC

Xa23R1 AGCCAGACCA AGGAAAAGCT GGTCAGTTCC GACGTGCGCC CGGCGGTGGA AAATGCGTTC GGTGCCCGTC

GPE 39 AGTCAGACCA AGGAAAAGTT GGTCAGTTCC GATGTGCGTC CGGCGGTCGA GAATGCCTTC GGCGCACGTC

MUS 060 AGTCAGACCA AGGAAAAGTT GGTCAGTTCC GATGTGCGTC CGGCGGTCGA GAATGCCTTC GGGGCACGTC

LMG 476 AGCCAGACCA AGGAAAAGCT GGTCAGCTCC GACGTGCGCC CGGCAGTGGA GAACGCCTTC GGCGCGCGCC

NCPPB4393 AGCCAGACCA AGGAAAAGCT GGTCAGCTCC GACGTGCGCC CGGCAGTGGA GAACGCCTTC GGCGCGCGCC

R1 AGCCAGACCA AGGAGAAGCT GGTCAGCTCC GACGTGCGCC CGGCGGTGGA GAACGCCTTC GGCGCGCGCC

XCV85-10 TCGCAGACCA AGGAAAAGCT GGTCAGCTCG GATGTGCGCC CGGCGGTGGA AAACGCCTTC GGTGCGCGCC

1121

GPE PC73 TGGAAGAGTT CCTGCAGGAG AACCCCAACG AGGCCAAGGC CATCGCCGGC AAGATCGTCG ATGCCGCGCG

Xa23R1 TGGAAGAGTT CCTGCAGGAG AACCCCAATG AGGCCAAGGC CATCGCCGGC AAGATCGTCG ATGCCGCGCG

GPE 39 TGGAAGAGTT CCTGCAAGAG AACCCCAACG AAGCCAAAGC CATCGCCGGC AAGATTGTCG ATGCCGCGCG

MUS 060 TGGAAGAGTT CCTGCAGGAG AACCCGAACG AAGCCAAAGC CATCGCCGGC AAGATTGTCG ATGCCGCACG

LMG 476 TGGAGGAGTT CCTGCAGGAA AACCCCAATG AGGCCAAGGC GATCGCCGGC AAGATCGTCG ATGCCGCCCG

NCPPB4393 TGGAGGAGTT CCTGCAGGAA AACCCCAATG AGGCCAAGGC GATCGCCGGC AAGATCGTCG ATGCCGCCCG

R1 TGGAGGAGTT CCTGCAGGAG AACCCCAACG AGGCCAAGGC CATCGCCGGC AAGATCGTCG ATGCCGCCCG

XCV85-10 TGCAGGAGTT CCTGCAGGAG AACCCGAACG AAGCCAAGGC CATCACCGGC AAGATCGTCG ATGCAGCGCG

1191

GPE PC73 CGCACGCGAG GCCGCGCGCA AGGCGCGCGA CCTGACCCGC CGCAAGGGCG CCTTGGATAT CGCGGGCCTG

Xa23R1 CGCACGCGAG GCCGCGCGCA AGGCGCGCGA CCTGACCCGC CGCAAGGGCG CCTTGGATAT CGCGGGCCTG

GPE 39 CGCCCGCGAG GCTGCACGCA AGGCACGCGA CCTGACCCGT CGCAAAGGTG CCTTGGATAT CGCCGGCCTG

MUS 060 CGCGCGCGAG GCCGCACGCA AGGCACGCGA CCTGACCCGT CGCAAAGGTG CCTTGGATAT CGCGGGCCTG

LMG 476 CGCCCGCGAG GCCGCGCGCA AGGCAAGAGA CCTGACCCGC CGCAAGGGCG CGCTGGACAT CGCCGGCCTG

NCPPB4393 CGCCCGCGAG GCCGCGCGCA AGGCAAGAGA CCTGACCCGC CGCAAGGGCG CGCTGGACAT CGCCGGCCTG

R1 CGCCCGTGAG GCCGCGCGCA AGGCAAGAGA CCTCACCCGT CGCAAGGGTG CGCTGGACAT CGCCGGCCTG

XCV85-10 CGCGCGCGAA GCCGCCCGCA AGGCGCGCGA CCTGACCCGC CGCAAGGGCG CGTTGGACAT CGCCGGCCTG

1261

GPE PC73 CCGGGCAAGC TCGCCGACTG CCAGGAAAAA GATCCAGCAC TATCTGAATT GTTCATTGTT GAGGGTGATT

Xa23R1 CCGGGCAAGC TCGCCGACTG CCAGGAAAAA GATCCAGCAC TATCTGAATT GTTCATTGTT GAGGGTGATT

GPE 39 CCCGGCAAGC TCGCCGATTG CCAGGAAAAA GACCCTGCGC TATCTGAACT GTTCATTGTC GAGGGTGACT

MUS 060 CCGGGCAAGC TCGCCGATTG CCAGGAAAAA GATCCGGCGC TATCTGAATT GTTCATTGTT GAGGGTGACT

LMG 476 CCCGGCAAGC TGGCCGACTG CCAGGAAAAG GATCCGGCGC TGTCGGAGCT GTTCATCGTC GAGGGTGACT

NCPPB4393 CCCGGCAAGC TGGCCGACTG CCAGGAAAAG GATCCGGCGC TGTCGGAGCT GTTCATCGTC GAGGGTGACT

R1 CCCGGCAAGC TGGCCGATTG CCAGGAAAAG GATCCGGCGC TGTCGGAGCT GTTCATCGTC GAGGGCGACT

XCV85-10 CCCGGCAAGC TCGCCGATTG CCAGGAAAAG GACCCGGCGC TGTCGGAACT GTTCATCGTC GAGGGTGACT

1331

GPE PC73 CAGCGGGCGG CTCGGCCAAG CAGGGTCGGA ATCGCAGGAA TCAGGCGGTA CTGCCCTTGC GCGGCAAGAT

Xa23R1 CAGCGGGCGG CTCGGCCAAG CAGGGCCGGA ATCGCAGGAA TCAGGCGGTA CTGCCCTTGC GCGGCAAGAT

GPE 39 CGGCAGGTGG CTCGGCCAAG CAGGGCCGCA ATCGCAAGAA TCAGGCGGTC CTGCCCTTGC GTGGCAAGAT

MUS 060 CGGCGGGGGG CTCGGCCAAG CAGGGCCGCA ATCGCAAGAA TCAGGCGGTC CTGCCCCTGC GTGGCAAGAT

LMG 476 CGGCAGGCGG CTCGGCCAAG CAGGGCCGCA ACCGCAAGAA CCAGGCGGTG CTGCCGCTGC GCGGCAAGAT

NCPPB4393 CGGCAGGCGG CTCGGCCAAG CAGGGCCGCA ACCGCAAGAA CCAGGCGGTG CTGCCGCTGC GCGGCAAGAT

R1 CGGCAGGCGG CTCGGCCAAG CAGGGCCGCA ACCGCAAGAA CCAGGCGGTG CTGCCGTTGC GCGGCAAGAT

XCV85-10 CGGCGGGCGG TTCGGCCAAG CAGGGACGCA ACCGCAAGAA CCAGGCCGTG CTGCCGCTGC GCGGCAAGAT

1401

GPE PC73 CCTCAACGTC GAGCGTGCCC GTTTCGATCG CATGCTGGCC TCCGACCAGG TCGGGACGCT GATCACCGCA

Xa23R1 CCTCAACGTC GAGCGTGCCC GTTTCGATCG CATGCTGGCC TCCGACCAGG TCGGGACGCT GATCACCGCA

GPE 39 CCTCAACGTC GAGCGTGCGC GTTTCGATCG CATGCTGGCC TCCGACCAGG TCGGTACGCT GATCACCGCC

MUS 060 CCTCAACGTC GAGCGTGCGC GTTTTGATCG CATGCTGGCC TCCGACCAAG TCGGTACGCT GATCACCGCC

LMG 476 CCTCAACGTC GAGCGTGCGC GCTTCGACCG CATGCTGTCC TCCGACCAGG TCGGCACGCT GATCACCGCG

NCPPB4393 CCTCAACGTC GAGCGTGCGC GCTTCGACCG CATGCTGTCC TCCGACCAGG TCGGCACGCT GATCACCGCG

R1 CCTCAACGTC GAGCGCGCGC GCTTCGACCG CATGCTGTCC TCCGACCAGG TCGGCACGCT GATCACCGCG

XCV85-10 CCTCAATGTC GAACGTGCCC GCTTCGACCG CATGCTGGCC TCCGACCAGG TCGGCACGCT GATCACTGCG

1471

GPE PC73 TTGGGTACCG GCATCGGTCG CGACGAGTAC AACCCGGACA AACTGCGTTA CCACCGCATC ATCATCATGA

Xa23R1 TTGGGTACCG GCATCGGTCG CGACGAGTAC AACCCGGACA AACTGCGTTA CCACCGCATC ATCATCATGA

GPE 39 TTGGGGACCG GCATCGGTCG CGACGAGTAC AACCCGGACA AACTGCGCTA CCACCGCATC ATCATCATGA

MUS 060 TTGGGGACCG GCATCGGTCG CGACGAGTAC AACCCGGACA AACTGCGCTA CCACCGCATC ATCATCATGA

LMG 476 CTGGGCACCG GCATCGGCCG CGACGAGTAC AACCCGGACA AGCTGCGCTA CCACCGCATC ATCATCATGA

NCPPB4393 CTGGGCACCG GCATCGGCCG CGACGAGTAC AACCCGGACA AGCTGCGCTA CCACCGCATC ATCATCATGA

R1 CTGGGCACCG GCATCGGCCG CGACGAGTAC AACCCGGACA AGCTGCGCTA CCACCGCATC ATCATCATGA

XCV85-10 CTGGGCACCG GCATCGGTCG CGACGAGTAC AACCCGGACA AGCTGCGCTA CCACCGCATC ATCCTGATGA

1541

GPE PC73 CCGACGCCGA CGTCGACGGC GCCCACATCC GCACCTTGCT GCTGACCTTC TTCTACCGGC AGATGCCTGA

Xa23R1 CCGACGCCGA CGTCGACGGC GCCCACATCC GCACCTTGCT GCTGACCTTC TTCTACCGGC AGATGCCTGA

GPE 39 CCGACGCCGA CGTCGACGGT GCGCACATTC GGACGTTGCT GCTGACGTTC TTCTACCGGC AGATGCCGGA

MUS 060 CCGATGCCGA CGTCGACGGT GCGCACATTC GTACGTTGCT GCTGACGTTC TTCTACCGGC AGATGCCAGA

LMG 476 CCGACGCCGA CGTCGACGGC GCGCACATCC GCACCCTGCT GCTGACCTTC TTCTACCGGC AGATGCCGGA

NCPPB4393 CCGACGCCGA CGTCGACGGC GCGCACATCC GCACCCTGCT GCTGACCTTC TTCTACCGGC AGATGCCGGA

R1 CCGACGCCGA CGTCGACGGC GCGCACATCC GCACCCTGCT GCTGACCTTT TTCTACCGGC AGATGCCGGA

XCV85-10 CCGACGCCGA CGTGGACGGC TCGCACATCC GCACCCTGCT GCTGACCTTC TTCTACCGGC AGATGCCGGA

1611

GPE PC73 GCTGATCGAA CGCGGCTACG TCTACATCGG CTTGCCCCCG CTCTACCGGC TCAAGCAAGG TAAGCAGGAG

Xa23R1 GCTGATCGAA CGCGGCTACG TCTACATCGG CTTGCCCCCG CTCTACCGGC TCAAGCAAGG TAAGCAGGAG

GPE 39 GCTGATCGAA CGCGGCTACG TGTACATCGG CTTGCCCCCG CTCTACCGGC TCAAGCAAGG CAAGCAGGAG

MUS 060 GCTGATCGAA CGCGGCTACG TGTACATCGG TTTGCCCCCG CTCTATCGGC TCAAGCAAGG CAAGCAGGAG

LMG 476 GCTGATCGAG CGCGGCTACG TCTACATCGG CCTGCCGCCG CTGTACCGGC TCAAGCAGGG CAAGCAGGAG

NCPPB4393 GCTGATCGAG CGCGGCTACG TCTACATCGG CCTGCCGCCG CTGTACCGGC TCAAGCAGGG CAAGCAGGAG

R1 GTTGATCGAG CGCGGCTACG TCTACATCGG CCTGCCGCCG CTGTACCGGC TCAAGCAGGG CAAGCAGGAG

XCV85-10 GTTGATCGAG CGCGGCTACA TCTACATCGG CCTGCCGCCG CTGTACAAGC TCAAGCAGGG CAAGAGCGAG

1681

GPE PC73 CTTTACCTGA AGGACGACAA CGCGCTCAAC GTCTATCTGG CCAGCAGCGC GGTCGAGGGT GCGGCGTTGA

Xa23R1 CTTTACCTGA AGGACGACAA CGCGCTCAAC GTCTATCTGG CCAGCAGCGC GGTCGAGGGT GCGGCGTTGA

GPE 39 CTGTACCTGA AGGATGACAA CGCGCTCAAC GTCTACCTCG CCAGCAGCGC GGTCGAGGGT GCGGCACTGA

MUS 060 CTGTACCTGA AGGACGACAA CGCGCTCAAC GTCTACCTCG CCAGCAGCGC GGTCGAGGGT GCGGCGCTGA

LMG 476 CTGTACCTGA AGGACGACAA CGCGCTCAAC GCCTACCTGG CCAGCAGCGC GGTCGAAGGC GCGGCGCTGG

NCPPB4393 CTGTACCTGA AGGACGACAA CGCGCTCAAC GCCTACCTGG CCAGCAGCGC GGTCGAAGGC GCGGCGCTGG

R1 CTGTACCTGA AGGACGACAA CGCGCTCAAC GCCTACCTGG CCAGCAGCGC GGTCGAAGGC GCGGCGCTGG

XCV85-10 CTGTATCTGA AGGACGACGC GGCGCTCAAC GCCTACCTGG CTAGCAATGC GGTCGAGGGC GCGGCGCTGA

1751

GPE PC73 TCCCGGCGAG CGGCGAGCCG CCGATCACCG GCGCGGCACT GGAGAAGTTG TTGCTGCTGT TCGCCAGCGC

Xa23R1 TCCCGGCGAG CGGCGAGCCG CCGATCACCG GCGCGGCACT GGAGAAGTTG TTGCTGCTGT TCGCCAGCGC

GPE 39 TCCCGGCGAC CGGCGAGCCG CCGATCACCG GTGCCGCTCT GGAGAAGTTA TTGCTGCTGT TCGCCAGCGC

MUS 060 TCCCGGCGAC CGGCGAGCCG CCGATCACCG GTGCCGCTCT GGAGAAGTTG TTGCTGCTGT TCGCCAGCGC

LMG 476 TCCCGGCCAG CAACGAGCCG CCGATCACTG GCGCGGCGCT GGAGAAGCTG CTGCTGCTGT TCGCCAGCGC

NCPPB4393 TCCCGGCCAG CAACGAGCCG CCGATCACTG GCGCGGCGCT GGAGAAGCTG CTGCTGCTGT TCGCCAGCGC

R1 TTCCGGCCAG CAACGAGCCA CCGATCACCG GCGCGGCGCT GGAGAAGCTG CTGCTGCTGT TCGCCAGCGC

XCV85-10 TTCCGGCCAC CGACGAACCG CCGATCACCG GCGAAGCGCT GGAGAAATTG TTGATGCTGT TCACCAGCGC

1821

GPE PC73 CAATGAAACC GTGGTACGCA ACGCCCATCG CTACGATCCT GCACTGCTGA CCGCATTGAT CGACTTGCCG

Xa23R1 CAATGAAACC GTGGTACGCA ACGCCCATCG CTACGATCCT GCACTGCTGA CCGCATTGAT CGACTTGCCG

GPE 39 CAATGAAACT GTGGCACGCA ATGCCCATCG CTACGATCCG GCGCTGCTGA CCGCGTTGAT CGACTTGCCG

MUS 060 CAATGAAACC GTGGCACGCA ATGCCCATCG CTACGATCCG GCGCTGCTGA CCGCGTTGAT CGACTTGCCG

LMG 476 CAACGAGGCG GTGGCGCGCA ATGCGCACCG CTATGACCCG GCGCTGCTCA GCGCGCTGAT CGACCTGCCG

NCPPB4393 CAACGAGGCG GTGGCGCGCA ATGCGCACCG CTATGACCCG GCGCTGCTCA GCGCGCTGAT CGACCTGCCG

R1 CAACGAGGCG GTGGCGCGCA ATGCGCACCG CTACGACCCG GCGCTGCTGA GCGCGCTGAT CGACCTGCCG

XCV85-10 CAACGAAGCG ATCGCGCGCA ACGCACACCG CTACGACCCG GCCCTGCTCA CCGCGCTGAT CGACCTGCCG

1891

GPE PC73 CCACTGGACG TGGCCCAGCT CGAAGCGGAG GGCGAACGGC ATCCAAGCCT GGATGCGCTG CAAATGGTTC

Xa23R1 CCACTGGACG TGGCCCAGCT CGAAGCGGAG GGCGAACGGC ATCCAAGCCT GGATGCGCTG CAAATGGTTC

GPE 39 CCACTGGACG TGAACCAGCT CGAAGCCGAA GGCGAATTGC ATCCAAGCCT GGATGCACTG CAAACGGTTC

MUS 060 CCACTGGATG TCAACCAGCT CGAAGCCGAA GGCGAATTGC ATCCAAGCCT GGATGCACTG CAAACGGTTC

LMG 476 CCGCTGGACG TCGCCCAGCT GGAGAGCGAG GGCGAAGCGC ACCCGAGCCT GGACGCGCTG CAGGCGGTGC

NCPPB4393 CCGCTGGACG TCGCCCAGCT GGAGAGCGAG GGCGAAGCGC ACCCGAGCCT GGACGCGCTG CAGGCGGTGC

R1 CCGCTGGACG TGGCCCAGCT CGAGAGCGAG GGCGAGACGC ATCCGAGCCT GGACGCGCTG CAGGCGGTGC

XCV85-10 CCACTGGATG TGGAAAAACT GCAGGCCGAA GGCGACCAGC ATCCGACCCT GGATGCACTG CAGGCAGTGC

1961

GPE PC73 TCAATCGTGG TAACCTCGGT TCGGCCCGCT ACACCTTGCG CTTTCAGCCC GCCAACGAGC AGCGTTCGGC

Xa23R1 TCAATCGCGG TAACCTCGGT TCAGCCCGCT ACACCTTGCG CTTTCAGCCC GCCAACGAGC AGCGTTCGGC

GPE 39 TCAATCGCGG TAACTTGGGC TCGGCCCGCT ACACCTTGCG CTTCCAGCCG GCCAACGAGC AGCGTCCGGC

MUS 060 TCAATCGCGG TAACTTGGGT TCGGCCCGTT ACACCTTGCG CTTCCAGCCG GCCAACGAGC AGCGTCCGGC

LMG 476 TCAACCGCGG CAGCCTGGGC TCGGCGCGCT ATGCGCTGTC CTTCGAACGC GGCAGCGAGC AGAAGCCGGC

NCPPB4393 TCAACCGCGG CAGCCTGGGC TCGGCGCGCT ATGCGCTGTC CTTCGAACGC GGCAGCGAGC AGAAGCCGGC

R1 TCAACCGCGG CAGCCTGGGC TCGGCGCGCT ACAGCCTGCG TTTCCAGGCG TCCAACGTGC AGCGCCCGGC

XCV85-10 TCAACCGCGG CACCCTGGGT ACCGCGCGCT ACCAGTTGCG CTTCGACCCC GGCAGCGAGA ACGCGCCTGC

2031

GPE PC73 CACCTTGCTG TTGGTGCGTC GCCACATGGG CGAGGAAATG ACCCAGGTGG TGCCGATGGC GGTGTTCGAA

Xa23R1 CACCTTGCTG TTGGTGCGTC GCCACATGGG CGAGGAAATG ACCCAGGTGG TGCCGATGGC GGTGTTCGAA

GPE 39 CACCTTGCTG TTGGTGCGTC GCCACATGGG CGAGGAAATG ACCCAGGTGG TGCCGATGGC GGTGTTCGAA

MUS 060 CACCTTGCTG TTGGTGCGTC GCCACATGGG CGAGGAAATG ACCCAGGTGG TGCCGATGGC GGTGTTCGAA

LMG 476 GATGTTGAAG GTGATCCGTC GCCACATGGG CGAAGAGCTC ACCAGCTGGG TGCCGATGGC CGCCTTCGAG

NCPPB4393 GATGTTGAAG GTGATCCGTC GCCACATGGG CGAAGAGCTC ACCAGCTGGG TGCCGATGGC CGCCTTCGAG

R1 CACCCTGCTG GTGGAGCGCC GGCACATGGG CGAGGAGACC ACCTATGTGG TGCCGATGGC GGTGTTCGAG

XCV85-10 CACCCTGGTG GCCATCCGCC GCCACATGGG CGAAGAGTTC ACCCAGGTGC TGCCGATGGG TGCGTTCGAA

2101

GPE PC73 AGCGGCGAAC TGCGGGCGTT GCGCGAGGTC GCGCTGGCGC TGCACGGTCT GGTCCGCGAG GGCGCGCAGA

Xa23R1 AGCGGCGAAC TGCGGGCGTT GCGCGAGGTC GCGCTGGCGC TGCACGGTCT GGTCCGCGAG GGCGCGCAGA

GPE 39 AGCGGCGAAT TGCGGCCGTT GCGCGAGGTC GCGTTGGCGT TGCACGGTCT GGTGCGCGAG GGTGCGCAAA

MUS 060 AGCGGCGAAT TGCGGCCGTT GCGCGAGGTC GCGTTGGCGC TGCACGGTCT GGTGCGCGAG GGTGCGCAAA

LMG 476 AGCGGCGAAC TGCGGGCCTT GCGCGAGGTC GCGCTGGCGC TGCACGGGCT GGTGCGCGAG GGTGCGCAGA

NCPPB4393 AGCGGCGAAC TGCGGGCCTT GCGCGAGGTC GCGCTGGCGC TGCACGGGCT GGTGCGCGAG GGTGCGCAGA

R1 AGCGGCGAGC TGCGGCCGCT GCGTGAGATC GCGCGGGAGT TGCATGGGCT GGTGCGCGAC GGCGCGCAGA

XCV85-10 AGCGGCGAGC TGCGTCCGCT GCGTGAGGTC TCGCTGGCCT TGCACGACCT GGTGCGCGAA GGTGCCCAGA

2171

GPE PC73 TCGTGCGCGG CAACAAGACC CAAGCCATCT CCAGCTTTGC CCAGGCGCAT GCCTGGCTGT TCGAAGAAGC

Xa23R1 TCGTGCGCGG CAACAAGACC CAAGCCATCT CCAGCTTTGC CCAGGCGCAT GCCTGGCTGT TCGAAGAAGC

GPE 39 TCGTGCGTGG CAATAAGACC CAAGCCATCT CCAGCTTTGC GCAAGCGCAT GCCTGGTTGT TCGAAGAAGC

MUS 060 TCGTGCGTGG CAATAAAACC CAAGCCATCT CCAGCTTTGC GCAAGCGCAT GCCTGGTTGT TCGAAGAAGC

LMG 476 TCGTGCGCGG CAACAAGACC CAGGCCATCA GCAGCTTCGC CCAGGCGCAC GCCTGGCTGT TCGAGGAGGC

NCPPB4393 TCGTGCGCGG CAACAAGACC CAGGCCATCA GCAGCTTCGC CCAGGCGCAC GCCTGGCTGT TCGAGGAGGC

R1 TCGTGCGCGG CAACAAGACC CAGGCCATCA CCAGCTTCGC CCAGGCCTAC GCCTGGCTGT TCGAGGAGGC

XCV85-10 TCGTGCGCGG CAACAAGAGC CACCCGATCA CCAGCTTCGC GCAGGCCCAT GCCTGGTTGC TGGACGAGGC

2241

GPE PC73 CAAAAAAGGC CGTCAGATCC AGCGCTTCAA GGGTCTGGGC GAAATGAATG CCGAACAGCT GTGGGAAACC

Xa23R1 CAAAAAAGGC CGTCAGATCC AGCGCTTCAA GGGTCTGGGC GAAATGAATG CCGAACAGCT GTGGGAAACC

GPE 39 CAAAAAAGGC CGTCAGATCC AGCGCTTCAA GGGTCTGGGC GAAATGAACG CCGAACAGTT GTGGGAAACC

MUS 060 CAAGAAAGGC CGTCAGATCC AGCGCTTCAA GGGTCTGGGC GAAATGAATG CCGAACAGTT GTGGGAAACC

LMG 476 CAAGAAGGGC CGCCAGATCC AGCGCTTCAA GGGCCTGGGC GAAATGAACG CCGAGCAGCT GTGGGAGACC

NCPPB4393 CAAGAAGGGC CGCCAGATCC AGCGCTTCAA GGGCCTGGGC GAAATGAACG CCGAGCAGCT GTGGGAGACC

R1 CAAGAAGGGC CGCCAGATCC AGCGCTTCAA GGGCCTGGGC GAAATGAACG CCGAGCAGCT GTGGGAGACC

XCV85-10 CAAGAAGGGC CGCCAGGTGC AGCGCTTCAA GGGCCTGGGC GAAATGAATG CCGAGCAGCT GTGGGAAACC

2311

GPE PC73 ACGGTCAACC CCGACACCCG CCGCTTGTTG CAGGTGCGCA TCGAGGACGC CGTGGCAGCC GATCAGATTT

Xa23R1 ACGGTCAACC CCGACACCCG CCGCTTGTTG CAGGTGCGCA TCGAGGACGC CGTGGCAGCC GATCAGATTT

GPE 39 ACGGTCAACC CCGACACCCG CCGTCTGTTG CAGGTGCGTA TCGAGGACGC TGTGGCAGCC GATCAAATCT

MUS 060 ACGGTCAACC CCGACACCCG CCGTCTATTG CAGGTGCGTA TCGAGGACGC CGTGGCGGCC GATCAAATCT

LMG 476 ACGGTGAACC CGGACACGCG CCGCCTGCTG CAGGTGCGCA TCGAGGACGC GGTCGCCGCC GACCAGATCT

NCPPB4393 ACGGTGAACC CGGACACGCG CCGCCTGCTG CAGGTGCGCA TCGAGGACGC GGTCGCCGCC GACCAGATCT

R1 ACGGTGAACC CGGATACGCG TCGCCTGCTG CAGGTGCGCA TCGAGGACGC GGTCGCCGCC GACCAGATCT

XCV85-10 ACGGTCAACC CCGACACGCG TCGCCTGTTG CAGGTGCGCA TCGAGGACGC GGTGGCCGCC GACCAGATCT

2381

GPE PC73 TCAGCACCTT GATGGGCGAT GTGGTCGAGC CGCGTCGTGA TTTCATCGAG GAAAACGCGC TAAAGGTCGC

Xa23R1 TCAGCACCTT GATGGGCGAT GTGGTCGAGC CGCGTCGTGA TTTCATCGAG GAAAACGCGC TAAAGGTCGC

GPE 39 TCAGCACCTT GATGGGCGAT GTGGTCGAAC CGCGTCGTGA TTTCATCGAG GAAAACGCGC TAAAGGTCGC

MUS 060 TCAGCACCTT GATGGGCGAT GTGGTCGAAC CGCGTCGTGA TTTCATCGAG GAAAACGCGC TAAAGGTCGC

LMG 476 TCAGCACCCT GATGGGCGAT GTGGTCGAAC CGCGCCGCGA CTTCATCGAA GAGAACGCGC TGAAGGTCGC

NCPPB4393 TCAGCACCCT GATGGGCGAT GTGGTCGAAC CGCGCCGCGA CTTCATCGAA GAGAACGCGC TGAAGGTCGC

R1 TCAGCACGCT GATGGGCGAC GTGGTCGAAC CGCGCCGCGA CTTCATCGAA GAGAACGCGC TGAAGGTCGC

XCV85-10 TCAGCACCTT GATGGGCGAT GTGGTCGAGC CACGCCGCGA CTTCATCGAG GACAACGCGC TGAAGGTGTC

2451

GPE PC73 CAACCTCGAC ATTTGAATGA GTCAGGGCAA GATCGTTCAG ATCATCGGCG CGGTCGTCGA CGTCGAATTC

Xa23R1 CAACCTCGAC ATTTGAATGA GTCAGGGCAA GATCGTTCAG ATCATCGGCG CGGTCGTCGA CGTCGAATTC

GPE 39 CAACCTCGAC ATTTGAATGA GTCAGGGCAA GATCGTTCAG ATCATCGGCG CAGTCGTCGA CGTCGAATTC

MUS 060 CAACCTGGAC ATTTGAATGA GTCAGGGCAA GATCGTTCAG ATCATCGGCG CGGTCGTCGA CGTCGAATTC

LMG 476 CAACCTCGAC ATCTGAATGA GTCAGGGCAA GATCGTTCAG ATCATCGGCG CGGTCGTCGA CGTCGAATTC

NCPPB4393 CAACCTCGAC ATCTGAATGA GTCAGGGCAA GATCGTTCAG ATCATCGGCG CGGTCGTCGA CGTCGAATTC

R1 CAACCTCGAC ATCTGAATGA GTCAGGGCAA GATCGTTCAG ATCATCGGCG CGGTCGTCGA CGTCGAATTC

XCV85-10 CAACCTGGAT ATCTGAATGA GTCAGGGCAA GATCGTTCAG ATCATCGGCG CGGTCGTCGA CGTCGAGTTC

2521

GPE PC73 GCGCGGGCCG ATGTGCCGAA GATTTACGAC GCATTGAAGG TCGAAGGCAC CGCCATCACG CTGGAAGTAC

Xa23R1 GCACGGGCCG ATGTGCCGAA GATTTACGAC GCGTTGAAGG TCGAAGGCAC CGCCATCACG CTGGAAGTAC

GPE 39 GCGCGGGCCG ATGTGCCGAA GATTTACGAC GCATTGAAGG TCGAAGGCAC CGCCATCACG CTGGAAGTGC

MUS 060 GCGCGGGCCG ATGTGCCGAA GATTTACGAC GCATTGAAGG TCGAAGGCAC CGCCATCACG CTGGAAGTAC

LMG 476 GCGCGTGCCG ATGTGCCGAA GATTTACGAC GCACTGAAGG TCGAAGGCAC CGCCATCACG CTGGAAGTGC

NCPPB4393 GCGCGTGCCG ATGTGCCGAA GATTTACGAC GCACTGAAGG TCGAAGGCAC CGCCATCACG CTGGAAGTGC

R1 GCGCGTGCCG ATGTGCCGAA GGTGTACGAC GCACTGAAGG TCGACGGCAC CGCCATCACG CTGGAAGTGC

XCV85-10 CAGCGCAATG AAGTGCCGAA GGTGTACGAC GCGCTGAAGG TCGAAGGCAC CGCCATCACC CTGGAAGTGC

2591

GPE PC73 AGCAGCAGCT GGGCGATGGC GTTGTGCGCA CCATTGCCCT CGGCTCCACC GATGGCCTCA AGCGCAACCT

Xa23R1 AGCAGCAGCT GGGCGATGGC GTCGTGCGCA CTATTGCCCT CGGCTCCACC GATGGCCTCA AGCGCAACCT

GPE 39 AGCAGCAGTT GGGCGATGGC GTCGTGCGCA CCATCGCCCT CGGCTCCACC GATGGCCTGA AGCGCAATCT

MUS 060 AGCAGCAGTT GGGCGATGGC GTCGTGCGCA CCATCGCCCT CGGCTCCACC GATGGCCTGA AGCGCAATCT

LMG 476 AGCAGCAGCT GGGCGACGGC GTCGTGCGCA CCATCGCGCT CGGCTCCACC GACGGCCTCA AGCGCCACCT

NCPPB4393 AGCAGCAGCT GGGCGACGGC GTCGTGCGCA CCATCGCGCT CGGCTCCACC GACGGCCTCA AGCGCCACCT

R1 AGCAGCAGTT GGGCGACGGC GTCGTGCGTA CGATCGCCCT CGGCTCCACC GACGGCCTCA AGCGCAACCT

XCV85-10 AGCAGCAGCT GGGCGACGGC GTCGTGCGCA CCATTGCGCT CGGTTCCACC GACGGCCTCA AGCGCAACCT

2661

GPE PC73 GGTCGCGACC AACACTGGCC GCGCGATCGC GGTGCCGGTC GGCGCGGGCA CGCTGGGCCG CATCATGGAC

Xa23R1 GGTCGCGACC AACACCGGTC GCGCGATCGC GGTGCCGGTA GGCGCGGGCA CGCTGGGCCG CATCATGGAC

GPE 39 GATCGCGACC AACACCGGCC GCGCGATCGC GGTGCCGGTC GGCGCGGGTA CGCTGGGCCG CATCATGGAC

MUS 060 GATCGCGACC AACACCGGCC GCGCGATCGC GGTGCCGGTC GGCGCGGGTA CGCTGGGCCG CATCATGGAT

LMG 476 GGTCGCCACC AACACCGGCA AGGCGATCGC CGTGCCGGTC GGCACCGCCA CCCTGGGCCG CATCATGAAC

NCPPB4393 GGTCGCCACC AACACCGGCA AGGCGATCGC CGTGCCGGTC GGCACCGCCA CCCTGGGCCG CATCATGAAC

R1 GGTCGCCACC AACACCGGCC GTGCGATCTC GGTGCCGGTC GGCGCCGGCA CGCTGGGCCG CATCATGGAC

XCV85-10 GGTCGCCACC AACACCGAAC GCGCCATCTC GGTGCCGGTC GGCGCCGGTA CCCTGGGCCG CATCATGGAC

2731

GPE PC73 GTGCTGGGCC GTCCGATCGA CGAAGCCGGT GAGGTGCAGG CCACCGACCA TTGGGAAATC CACCGCGCTG

Xa23R1 GTGCTGGGCC GTCCGATCGA CGAAGCCGGC GAGGTGAAGG CCACCGACCA TTGGGAAATC CACCGCGCTG

GPE 39 GTGCTGGGCC GTCCGATCGA CGAAGCCGGC GAGGTGCAGG CCACCGACCA TTGGGAAATC CACCGCGCTG

MUS 060 GTGCTGGGCC GTCCGATCGA CGAAGCCGGC GAGGTGCAGG CCACCGACCA TTGGGAAATC CACCGCGCTG

LMG 476 GTGCTCGGCG AGCCGATCGA CGAGCGCGGC CCGGTCCAGT CCGACGTGCA GTGGGAAATC CACCGCGAGG

NCPPB4393 GTGCTCGGCG AGCCGATCGA CGAGCGCGGC CCGGTCCAGT CCGACGTGCA GTGGGAAATC CACCGCGAGG

R1 GTGCTGGGTC GTCCGATCGA CGAGGCCGGC GACGTGCAGG CCACCGACCA TTGGGAAATC CACCGCGCGG

XCV85-10 GTGCTGGGTC GCCCGATCGA CGAGGCCGGC GACGTGCAGG CGTCCGATCA TTGGGAAATC CACCGTAGCG

2801

GPE PC73 CGCCGACATA TGAAGATCAG TCCTCGGCCA CCGAATTGCT GGAAACCGGC ATCAAGGTCA TCGACCTGAT

Xa23R1 CGCCGACATA TGAAGATCAG TCCTCGGCCA CCGAATTGCT GGAAACCGGC ATCAAGGTCA TCGACCTGAT

GPE 39 CGCCGACATA CGAAGATCAG TCCTCGGCCA CCGAATTGCT GGAAACCGGC ATCAAGGTCA TCGACCTGAT

MUS 060 CGCCGACATA CGAAGATCAG TCCTCGGCCA CCGAATTGCT GGAAACCGGC ATCAAGGTCA TCGACCTGAT

LMG 476 CGCCCGACTA CGCCGACCAG TCCTCCAGCA CCGAACTGCT GGAAACCGGC ATCAAGGTCA TCGACCTGAT

NCPPB4393 CGCCCGACTA CGCCGACCAG TCCTCCAGCA CCGAACTGCT GGAAACCGGC ATCAAGGTCA TCGACCTGAT

R1 CGCCGACCTA CGAAGACCAG TCCTCGGCCA CCGAGCTGCT GGAGACCGGC ATCAAGGTCA TCGACCTGAT

XCV85-10 CCCCGTCCTA CGAGGACCAG GCGTCGACCA CCGAACTGCT GGAAACCGGC ATCAAGGTGA TCGACCTGAT

2871

GPE PC73 GTGCCCGTTC GCCAAGGGCG GCAAGGTCGG CCTGTTCGGC GGCGCCGGTG TCGGCAAGAC CGTCAACATG

Xa23R1 GTGCCCGTTC GCCAAGGGCG GTAAGGTTGG CCTATTCGGC GGCGCCGGTG TCGGCAAGAC CGTCAACATG

GPE 39 GTGCCCGTTC GCCAAGGGCG GTAAGGTCGG CCTGTTCGGC GGCGCCGGTG TCGGCAAGAC CGTCAACATG

MUS 060 GTGCCCGTTC GCCAAGGGCG GCAAGGTCGG CCTGTTCGGC GGCGCCGGTG TCGGCAAGAC CGTCAACATG

LMG 476 GTGCCCGTTC GCCAAGGGCG GCAAGGTCGG CCTGTTCGGC GGCGCCGGCG TCGGCAAGAC CGTCAACATG

NCPPB4393 GTGCCCGTTC GCCAAGGGCG GCAAGGTCGG CCTGTTCGGC GGCGCCGGCG TCGGCAAGAC CGTCAACATG

R1 GTGCCCGTTC GCCAAGGGCG GCAAGGTCGG CCTGTTCGGC GGCGCCGGCG TCGGCAAGAC CGTCAACATG

XCV85-10 GTGCCCGTTC GCCAAGGGCG GCAAGGTCGG CCTGTTCGGC GGCGCCGGCG TCGGCAAGAC CGTCAACATG

2941

GPE PC73 ATGGAGCTGA TCAACAACAT CGCCAAGGCA CACGCAGGCT TGTCGGTGTT TGCTGGCGTG GGCGAGCGGA

Xa23R1 ATGGAACTGA TCAACAACAT CGCCAAGGCA CACGCGGGCT TGTCGGTGTT CGCTGGCGTG GGCGAGCGGA

GPE 39 ATGGAACTGA TCAACAACAT CGCCAAGGCG CACGCGGGCC TGTCGGTGTT CGCCGGCGTG GGCGAGCGCA

MUS 060 ATGGAACTGA TCAACAACAT CGCCAAGGCG CACGCGGGCC TGTCGGTGTT CGCCGGCGTG GGCGAGCGCA

LMG 476 CTGGAATTGA TCAACAACAT CGCGACCGAG CACTCGGGCC TGTCGGTGTT CGCCGGCGTC GGCGAGCGGA

NCPPB4393 CTGGAATTGA TCAACAACAT CGCGACCGAG CACTCGGGCC TGTCGGTGTT CGCCGGCGTC GGCGAGCGGA

R1 ATGGAACTGA TCAACAACAT CGCCAAGGCG CACTCGGGTC TGTCCGTGTT CGCCGGCGTG GGCGAGCGTA

XCV85-10 ATGGAACTGA TCAACAACAT CGCCAAGGCG CACTCGGGTC TGTCGGTGTT CGCCGGCGTG GGCGAGCGTA

3011

GPE PC73 CCCGCGAGGG CAACGATTTC TACCACGAAA TGAAGGACTC CAACGTCCT- ---------- ----------

Xa23R1 CCCGCGAGGG CAACGACTTC TACCACGAAA TGAAGGACTC CAACGTCCT- ---------- ----------

GPE 39 CCCGCGAGGG CAACGACTTC TACCACGAGA TGAAAGACTC CAACGTCCT- ---------- ----------

MUS 060 CCCGCGAGGG CAACGACTTC TACCACGAGA TGAAAGACTC CAACGTCCT- ---------- ----------

LMG 476 CCCGCGAGGG CAACGACTTC TATCACGAGA TGAGCGACGC CAACGTCATC GTCCAGGACG ACCTGAGCAA

NCPPB4393 CCCGCGAGGG CAACGACTTC TATCACGAGA TGAGCGACGC CAACGTCATC GTCCAGGACG ACCTGAGCAA

R1 CCCGCGAGGG CAACGACTTC TACCACGAGA TGAAGGACTC CAACGTCCT- ---------- ----------

XCV85-10 CCCGCGAGGG CAACGACTTC TACCACGAGA TGAAGGACTC CAACGTCCT- ---------- ----------

3081

GPE PC73 GGACAAGGTC GCGATGGTGT ATGGCCAGAT GAACGAGCCG CCGGGCAACC GATTGCGCGT CGCACTGACC

Xa23R1 GGACAAGGTC GCGATGGTGT ACGGCCAGAT GAACGAGCCG CCGGGCAATC GCCTGCGCGT CGCACTGACC

GPE 39 GGACAAGGTC GCGATGGTGT ACGGCCAGAT GAACGAGCCG CCGGGCAACC GCCTGCGCGT CGCGCTGACC

MUS 060 GGACAAGGTC GCGATGGTGT ACGGCCAGAT GAACGAGCCG CCGGGCAACC GCCTGCGCGT CGCGCTGACC

LMG 476 GTCCAAGGTG GCGATGGTGT ACGGCCAGAT GAACGAGCCG CCGGGCAACC GTCTGCGCGT GGCGCTGACC

NCPPB4393 GTCCAAGGTG GCGATGGTGT ACGGCCAGAT GAACGAGCCG CCGGGCAACC GTCTGCGCGT GGCGCTGACC

R1 GGACAAGGTG GCGATGGTGT ACGGCCAGAT GAACGAGCCG CCGGGCAACC GTCTGCGCGT CGCGCTGACC

XCV85-10 GGACAAGGTG GCGATGGTGT ACGGCCAGAT GAACGAGCCG CCGGGCAACC GTCTGCGCGT GGCGTTGACC

3151

GPE PC73 GGCCTGACCA TGGCCGAGTA CTTCCGCGAC GAAAAGGATG CTTCGGGCAA GGGCAAGGAC GTGCTGCTGT

Xa23R1 GGCCTGACCA TGGCCGAGTA CTTCCGCGAC GAGAAGGATG CCTCGGGCAA GGGCAAGGAC GTGCTGCTGT

GPE 39 GGCCTGACCA TGGCCGAGTA CTTCCGCGAC GAGAAGGATG CCTCGGGCAA GGGTAAGGAC GTGCTGTTGT

MUS 060 GGCCTGACCA TGGCCGAGTA CTTCCGTGAC GAGAAGGATG CCTCGGGCAA GGGTAAGGAC GTGCTGTTGT

LMG 476 GGCCTGACCA TGGCCGAGTA CTTCCGCGAC GAAAAGGACG CCAACGGCAA GGGCCGCGAC GTGCTGTTCT

NCPPB4393 GGCCTGACCA TGGCCGAGTA CTTCCGCGAC GAAAAGGACG CCAACGGCAA GGGCCGCGAC GTGCTGTTCT

R1 GGCCTGACCA TGGCCGAGTA CTTCCGCGAC GAGAAGGACG CCTCGGGCAA GGGCAAGGAC GTGCTGCTGT

XCV85-10 GGCCTGACCA TGGCCGAGTA CTTCCGCGAC GAGAAGGATG CCAGCGGCAA GGGCAAGGAC GTGCTGCTGT

3221

GPE PC73 TCGTCGATAA CATCTACCGC TACACCCTGG CCGGTACCGA AGTGTCGGCG TTGCTGGGCC GCATGCCGTC

Xa23R1 TCGTCGACAA TATCTACCGC TACACCCTGG CCGGTACCGA AGTGTCGGCG CTGCTGGGCC GCATGCCGTC

GPE 39 TCGTCGATAA CATCTATCGC TACACCCTGG CCGGTACCGA AGTGTCGGCG CTGCTGGGCC GTATGCCGTC

MUS 060 TCGTCGATAA CATCTATCGC TACACCCTGG CCGGTACCGA AGTGTCGGCG CTGCTGGGGC GTATGCCGTC

LMG 476 TCGTCGACAA CATCTACCGC TACACCCTGG CCGGTACCGA AGTGTCGGCG CTGCTGGGCC GCATGCCGTC

NCPPB4393 TCGTCGACAA CATCTACCGC TACACCCTGG CCGGTACCGA AGTGTCGGCG CTGCTGGGCC GCATGCCGTC

R1 TCGTCGACAA CATCTACCGC TACACCCTGG CCGGTACCGA AGTGTCGGCG CTGCTGGGCC GCATGCCGTC

XCV85-10 TCGTGGACAA CATCTACCGC TACACGCTGG CCGGTACCGA AGTGTCGGCG CTGCTCGGCC GCATGCCGTC

3291

GPE PC73 GGCGGTGGGT TACCAGCCGA CCCTGGCCGA GGAAATGGGC GTGTTGCAGG AGCGCATCAC CTCCACCAAG

Xa23R1 GGCGGTGGGT TACCAGCCGA CCCTGGCCGA GGAAATGGGC GTGTTGCAGG AGCGCATCAC CTCCACCAAG

GPE 39 GGCGGTGGGT TACCAGCCGA CCCTGGCCGA GGAAATGGGC GTGTTGCAGG AGCGCATCAC CTCCACCAAG

MUS 060 GGCGGTGGGT TACCAGCCGA CCCTGGCCGA GGAAATGGGC GTGTTGCAGG AGCGCATCAC CTCCACCAAG

LMG 476 GGCGGTGGGC TACCAGCCGA CCCTGGCCGA GGAAATGGGC GTGCTGCAGG AGCGCATCAC CTCGACCAAG

NCPPB4393 GGCGGTGGGC TACCAGCCGA CCCTGGCCGA GGAAATGGGC GTGCTGCAGG AGCGCATCAC CTCGACCAAG

R1 GGCGGTGGGC TACCAGCCGA CCCTGGCCGA GGAAATGGGC GTGCTGCAGG AGCGCATCAC CTCGACCAAG

XCV85-10 GGCGGTGGGC TACCAGCCGA CCCTGGCCGA GGAAATGGGC GTGCTGCAGG AGCGCATCAC CTCGACCAAG

3361

GPE PC73 AACGGCTCGA TCACCTCGAT CCAGGCGGTG TACGTGCCCG CGGACGACCT CACCGACCCG TCGCCGGCGA

Xa23R1 AACGGCTCGA TCACCTCGAT CCAGGCGGTG TACGTGCCCG CGGACGACCT CACCGACCCG TCGCCGGCGA

GPE 39 AACGGCTCGA TCACCTCGAT CCAGGCGGTG TACGTGCCTG CGGACGACTT GACCGACCCG TCGCCTGCGA

MUS 060 AACGGCTCGA TCACCTCGAT CCAGGCGGTG TACGTGCCTG CGGACGACTT GACCGACCCG TCGCCTGCGA

LMG 476 ACCGGCTCGA TCACCTCGAT CCAGGCCGTG TACGTGCCCG CGGACGACCT GACCGACCCG TCGCCGGCGA

NCPPB4393 ACCGGCTCGA TCACCTCGAT CCAGGCCGTG TACGTGCCCG CGGACGACCT GACCGACCCG TCGCCGGCGA

R1 TCCGGCTCGA TCACCTCGAT CCAGGCCGTG TACGTGCCCG CGGACGACCT GACCGACCCG TCGCCGGCGA

XCV85-10 AGCGGTTCGA TCACCTCGAT CCAGGCCGTG TACGTGCCTG CGGACGACCT GACCGACCCG TCGCCGGCGA

3431

GPE PC73 CCACCTTCGC CCACCTCGAC GCCACCGTCG TGCTGTCGCG TAACATCGCC TCGCTGGGCA TCTATCCGGC

Xa23R1 CCACCTTCGC CCACCTCGAC GCCACCGTCG TGCTGTCGCG TAACATCGCC TCGCTGGGCA TCTATCCGGC

GPE 39 CCACCTTCGC CCACCTCGAC GCCACCGTGG TGCTGTCGCG TAACATCGCC TCGCTGGGTA TCTATCCGGC

MUS 060 CCACCTTCGC CCACCTCGAC GCCACCGTGG TGCTGTCGCG TAACATCGCC TCGCTGGGTA TCTATCCGGC

LMG 476 CCACCTTCGC CCACCTCGAC GCCACCGTCG TGCTGTCGCG TAACATCGCC TCGCTGGGTA TCTACCCGGC

NCPPB4393 CCACCTTCGC CCACCTCGAC GCCACCGTCG TGCTGTCGCG TAACATCGCC TCGCTGGGTA TCTACCCGGC

R1 CCACCTTCGC CCACCTCGAC GCCACCGTCG TGCTGTCGCG TAACATCGCT TCGCTGGGTA TCTACCCGGC

XCV85-10 CCACCTTCGC CCACCTGGAT TCCACCGTCA CGCTGAGCCG TAACATCGCC TCGCTGGGTA TCTACCCGGC

3501

GPE PC73 GGTGGACCCG TTGGATTCGA CCAGCCGCCA GCTCGATCCG AACGTGATCG GCCACGAGCA TTACGACACC

Xa23R1 GGTGGACCCG TTGGATTCGA CCAGCCGCCA GCTCGATCCG AACGTGATCG GCCACGAGCA TTACGACACC

GPE 39 GGTGGACCCG CTGGATTCGA CCAGCCGCCA GCTCGATCCG AACGTGATCG GCCACGAGCA TTACGACACC

MUS 060 GGTGGACCCG CTGGATTCGA CCAGCCGCCA GCTCGATCCG AACGTGATCG GCCACGAGCA TTACGACACC

LMG 476 GGTGGATCCG CTCGACTCGA CCAGCCGTCA GCTGGACCCG AACGTGATCG GCCATGAGCA CTACGACACC

NCPPB4393 GGTGGATCCG CTCGACTCGA CCAGCCGTCA GCTGGACCCG AACGTGATCG GCCATGAGCA CTACGACACC

R1 GGTGGATCCG CTCGATTCGA CCAGCCGCCA GCTGGACCCG AACGTGATCG GCCACGAGCA CTACGACACC

XCV85-10 GGTGGATCCG CTGGACTCGA CCAGCCGCCA GATGGATCCG CTGGTGATCG GCCACGAGCA TTACGACACC

3571

GPE PC73 GCGCGCCGCG TGCAGTCCAC CTTGCAGAAG TACAAGGAGC TGAAGGACAT CATCGCGATC CTGGGCATGG

Xa23R1 GCGCGCCGCG TGCAGTCCAC CTTGCAGAAG TACAAGGAGC TGAAGGACAT CATCGCGATC CTGGGCATGG

GPE 39 GCGCGCCGCG TGCAGTCCAC CTTGCAGAAG TACAAGGAGC TGAAGGACAT CATCGCGATC CTCGGCATGG

MUS 060 GCGCGCCGCG TGCAGTCCAC CTTGCAGAAG TACAAGGAGC TGAAGGACAT CATCGCGATC CTCGGCATGG

LMG 476 GCCCGCCGCG TGCAGTCCAC CTTGCAGAAG TACAAGGAAC TGAAGGACAT CATCGCGATC CTGGGCATGG

NCPPB4393 GCCCGCCGCG TGCAGTCCAC CTTGCAGAAG TACAAGGAAC TGAAGGACAT CATCGCGATC CTGGGCATGG

R1 GCCCGCCGCG TGCAGTCCAC CTTGCAGAAG TACAAGGAAC TGAAGGACAT CATCGCGATT CTGGGCATGG

XCV85-10 GCCCAGCGCG TCCAGCAGAC CTTGCAGAAG TACAAGGAAC TGAAGGACAT CATCGCCATC CTGGGCATGG

3641

GPE PC73 ACGAACTGTC CGAAGAGGAC AAGCAGGCCG TGTCGCGCGC GCGCAAGATC GAGCGCTTCT TCAGCCAGCC

Xa23R1 ACGAACTGTC CGAAGAGGAC AAGCAGGCCG TGTCGCGCGC GCGCAAGATC GAGCGCTTCT TCAGCCAGCC

GPE 39 ATGAACTGTC CGAAGAGGAT AAGCAGGCCG TCTCGCGTGC GCGCAAGATC GAACGCTTCT TCAGCCAACC

MUS 060 ATGAACTGTC CGAAGAGGAT AAGCAGGCCG TCTCGCGTGC GCGCAAGATC GAGCGCTTCT TCAGCCAACC

LMG 476 ACGAACTGTC CGAAGAGGAC AAGCAGGCCG TGTCGCGCGC GCGCAAGATC GAGCGCTTCT TCAGCCAGCC

NCPPB4393 ACGAACTGTC CGAAGAGGAC AAGCAGGCCG TGTCGCGCGC GCGCAAGATC GAGCGCTTCT TCAGCCAGCC

R1 ACGAACTGTC CGAAGAGGAC AAGCAGGCCG TGTCGCGCGC GCGCAAGATC GAGCGCTTCT TCAGCCAGCC

XCV85-10 ACGAGCTGAG CGAAGAAGAC AAGCAGTCGG TGTCGCGCGC GCGCAAGATC GAGCGCTTCT TCAGCCAGCC

3711

GPE PC73 GTTCCACGTG GCCGAAGTGT TCACCGGCTC GCCGGGCAAG TACGTGTCGC TGAAGGACAC CATCCGCGGC

Xa23R1 GTTCCACGTG GCCGAAGTGT TCACCGGCTC GCCGGGCAAG TACGTGTCGC TGAAGGACAC CATCCGCGGC

GPE 39 GTTCCACGTG GCCGAAGTGT TCACCGGCTC GCCGGGCAAG TACGTGTCGC TGAAGGAGAC CATCCGCGGC

MUS 060 GTTCCACGTG GCCGAAGTGT TCACCGGATC GCCGGGCAAG TACGTGTCGC TGAAGGACAC CATCCGCGGC

LMG 476 GTTCCACGTG GCCGAAGTGT TCACCGGCTC GCCGGGCAAG TACGTGTCGC TGAAGGACAC CATCCGCGGC

NCPPB4393 GTTCCACGTG GCCGAAGTGT TCACCGGCTC GCCGGGCAAG TACGTGTCGC TGAAGGACAC CATCCGCGGC

R1 GTTCCACGTG GCCGAAGTGT TCACCGGCTC GCCGGGCAAG TACGTGTCGC TGAAGGACAC CATCCGCGGC

XCV85-10 TTTCCACGTG GCCGAAGTGT TCACCGGTTC GCCGGGCAAG TACGTGTCGC TGAAGGACAC CATCCGCGGC

3781

GPE PC73 TTCAAGGCGA TCGTCGATGG CGAGTACGAC CACCTGCCGG AGCAGGCGTT CTACATGGTC GGCGGCATCG

Xa23R1 TTCAAGGCGA TCGTCGATGG CGAGTACGAC CACCTGCCGG AGCAGGCGTT CTACATGGTC GGCGGCATCG

GPE 39 TTCAAGGCGA TTGTCGATGG CGAGTACGAC CACCTGCCGG AGCAGGCGTT CTACATGGTC GGCGGCATCG

MUS 060 TTCAAGGCGA TCGTCGATGG CGAGTACGAC CACCTGCCGG AGCAGGCGTT CTACATGGTC GGCGGGATCG

LMG 476 TTCAAGGCCA TCGTCGACGG CGAATACGAC CACCTGCCGG AGCAGGCCTT CTACATGGTC GGCGGCATCG

NCPPB4393 TTCAAGGCCA TCGTCGACGG CGAATACGAC CACCTGCCGG AGCAGGCCTT CTACATGGTC GGCGGCATCG

R1 TTCAAGGCCA TCGTCGACGG CGAATACGAC CACCTGCCGG AGCAGGCCTT CTACATGGTC GGCGGCATCG

XCV85-10 TTCAAGGCGA TCTGCGACGG CGAATACGAC CACCTGCCGG AGCAGGCGTT CTACATGGTC GGCAGCATCG

3851

GPE PC73 AGGAAGCGGT CGAGAAAGCC AAGAAGATGG CCGAAAAGGC CTGAATGGGC AAGATCATCG GCATCGACCT

Xa23R1 AGGAAGCGGT CGAGAAAGCC AAGAAGATGG CCGAAAAGGC CTGAATGGGC AAGATCATCG GCATCGACCT

GPE 39 AGGAAGCGGT CGAAAAAGCC AAAAAGATGG CCGAAAAGGC CTGAATGGGC AAGATCATCG GCATCGACCT

MUS 060 AGGAAGCGGT CGAGAAAGCC AAAAAGATGG CCGAAAAGGC CTGAATGGGC AAGATCATCG GCATCGACCT

LMG 476 AGGAAGCGGT CGAGAAGGCC AAGAAGATGG CCGAGAAGGC GTAAATGGGC AAGATCATCG GCATCGACCT

NCPPB4393 AGGAAGCGGT CGAGAAGGCC AAGAAGATGG CCGAGAAGGC GTAAATGGGC AAGATCATCG GCATCGACCT

R1 AGGAAGCGGT CGAGAAGGCC AAGAAGATGG CCGAGAAGGC GTAAATGGGC AAGATCATCG GCATCGACCT

XCV85-10 AAGAAGCGGT CGAGAAAGCC AACAAGATG- ---------- ---AATGGGC AAGATCATTG GTATTGACCT

3921

GPE PC73 GGGCACGACC AATTCGTGCG TGGCGATCAT GGACGGCGGC AAGGCTCGCG TCATCGAGAA TTCCGAGGGC

Xa23R1 GGGCACGACC AATTCGTGCG TGGCGATCAT GGACGGCGGC AAGGCTCGCG TCATCGAGAA TTCCGAGGGC

GPE 39 GGGCACGACC AATTCGTGCG TGGCGATCAT GGACGGCGGC AAGGCTCGCG TCATCGAGAA TTCCGAGGGC

MUS 060 GGGCACGACC AATTCGTGCG TGGCGATCAT GGACGGCGGC AAGGCTCGCG TCATCGAAAA TTCCGAGGGC

LMG 476 GGGCACGACC AACTCGTGCG TGGCGATCAT GGACGGCGGC AAGGCCCGCG TCATCGAGAA TTCCGAGGGT

NCPPB4393 GGGCACGACC AACTCGTGCG TGGCGATCAT GGACGGCGGC AAGGCCCGCG TCATCGAGAA TTCCGAGGGT

R1 GGGCACGACC AATTCGTGCG TGGCGATCAT GGACGGCGGC AAGGCCCGCG TCATCGAGAA TTCCGAGGGT

XCV85-10 CGGCACCACG AACTCGTGCG TGTCGATCAT GGACGGCGGC AAGGCCCGCG TCATCGAAAA CTCCGAGGGC

3991

GPE PC73 GACCGCACCA CGCCTTCGAT CGTCGCCTAC ACCAAGGACG GCGAAGTGTT GGTGGGCGCC TCGGCCAAGC

Xa23R1 GACCGCACCA CGCCTTCGAT CGTCGCCTAC ACCAAGGACG GCGAAGTGTT GGTGGGCGCC TCGGCCAAGC

GPE 39 GACCGCACCA CGCCTTCGAT CGTCGCCTAC ACCAAGGACG GCGAAGTGTT GGTGGGCGCC TCGGCCAAGC

MUS 060 GACCGTACCA CGCCTTCGAT CGTCGCTTAC ACCAAGGATG GCGAAGTGTT GGTGGGTGCC TCGGCCAAGC

LMG 476 GACCGCACCA CGCCTTCGAT CGTCGCCTAC ACCAAGGACG GCGAAGTGCT GGTGGGCGCC TCGGCCAAGC

NCPPB4393 GACCGCACCA CGCCTTCGAT CGTCGCCTAC ACCAAGGACG GCGAAGTGCT GGTGGGCGCC TCGGCCAAGC

R1 GACCGCACCA CGCCTTCGAT CGTCGCCTAC ACCAAGGATG GCGAAGTGCT GGTGGGCGCC TCGGCCAAGC

XCV85-10 GATCGCACCA CGCCTTCGAT CGTCGCCTAC ACCAAGGACG GCGAAGTGCT GGTCGGCGCC TCGGCCAAGC

4061

GPE PC73 GCCAGGCGGT GACCAACCCG AAGAACACCT TCCATGCGGT CAAGCGTCTG ATCGGCCGCA AGTTCGGCGA

Xa23R1 GCCAGGCGGT GACCAACCCG AAGAACACCT TCCATGCGGT CAAGCGTCTG ATCGGCCGCA AGTTCGGCGA

GPE 39 GCCAGGCGGT GACCAACCCG AAGAACACCT TCCATGCGGT CAAGCGTCTG ATCGGCCGTA AATTCGGCGA

MUS 060 GTCAGGCAGT GACCAACCCG AAGAACACTT TCTACGCGGT CAAGCGTCTG ATCGGCCGTA AATTCGGCGA

LMG 476 GCCAGGCCGT CACCAACCCC AAGAACACCT TCTACGCGGT CAAGCGCCTG ATCGGCCGCA AGTTCACCGA

NCPPB4393 GCCAGGCCGT CACCAACCCC AAGAACACCT TCTACGCGGT CAAGCGCCTG ATCGGCCGCA AGTTCACCGA

R1 GCCAGGCCGT CACCAACCCC AAGAACACCT TCTACGCGGT CAAGCGCCTG ATCGGCCGCA AGTTCACCGA

XCV85-10 GCCAGGCCGT GACCAACCCG AAGAACACCT TCTACGCGGT GAAGCGCCTG ATCGGCCGCA AGTTCACCGA

4131

GPE PC73 TGCCGAAGTG CAGAAGGACA TCGGCCTGGT GCCTTACGCC ATCGCTCAGC ACGACAACGG CGATGCCTGG

Xa23R1 TGCCGAAGTG CAGAAGGACA TCGGCCTGGT GCCTTACGCC ATCGCTCAGC ACGACAACGG CGATGCCTGG

GPE 39 TGCCGAAGTG CAGAAGGACA TTGGCCTGGT CCCTTACTCC ATCGCCCAGC ACGACAACGG CGATGCCTGG

MUS 060 TGCCGAAGTG CAGAAGGACA TTGGCCTGGT CCCTTACTCC ATCGCCCAGC ACGACAACGG CGATGCCTGG

LMG 476 CGCCGAAGTC CAGAAGGACA TCGGCCTGGT GCCGTACAGC ATCGTCCAGC ACGACAACGG CGACGCCTGG

NCPPB4393 CGCCGAAGTC CAGAAGGACA TCGGCCTGGT GCCGTACAGC ATCGTCCAGC ACGACAACGG CGACGCCTGG

R1 CGCCGAAGTG CAGAAGGACA TCGGCCTGGT GCCGTACAGC ATCGTCCAGC ACGACAACGG CGACGCCTGG

XCV85-10 TGGCGAAGTG CAGAAGGACA TCTCGCACGT GCCGTACGGC ATCCTGGCGC ACGACAACGG CGACGCCTGG

4201

GPE PC73 GTGGCCACTG CCGATGGACG CAAGCTGGCG CCGCAGGAAA TTTCCGCACA GGTGCTGGAA AAGATGAAAA

Xa23R1 GTGGCCACTG CCGACGGACG CAAGCTGGCG CCGCAGGAAA TTTCCGCGCA GGTGCTGGAA AAGATGAAAA

GPE 39 GTGGCCACCG CCGACGGACG CAAGCTGGCA CCGCAGGAAA TTTCCGCGCA GGTGCTGGAA AAGATGAAAA

MUS 060 GTGGCCACCG CCGACGGACG CAAGCTGGCG CCGCAGGAAA TTTCCGCGCA GGTGCTGGAA AAGATGAAAA

LMG 476 GTGGCCACCG CCGACGGGCG CAAGCTGGCC TCGCAGGAAA TCTCCGCGCA GGTGCTGGAG AAGATGAAGA

NCPPB4393 GTGGCCACCG CCGACGGGCG CAAGCTGGCC TCGCAGGAAA TCTCCGCGCA GGTGCTGGAG AAGATGAAGA

R1 GTGGCCACCG CCGACGGGCG CAAGCTGGCC TCGCAGGAAA TCTCCGCGCA GGTGCTGGAG AAGATGAAGA

XCV85-10 GTGCAGACCA GCGATGCCAA GCGCATGGCG CCGCAGGAAA TCTCCGCGCG CGTGCTGGAG AAGATGAAGA

4271

GPE PC73 AGACCGCCGA GGCGTTCCTT GGCGAGAAGG TGACCGAAGC GGTCATCACC GTGCCGGCGT ACTTCAACGA

Xa23R1 AGACCGCCGA GGCGTTCCTT GGCGAGAAGG TGACCGAAGC GGTCATCACC GTGCCGGCGT ACTTCAACGA

GPE 39 AGACCGCTGA GGCGTTTCTC GGCGAGACGG TGACCGAGGC CGTCATCACC GTGCCGGCGT ACTTCAACGA

MUS 060 AGACCGCCGA GGCGTTTCTC GGGGAGACGG TGACCGAGGC CGTCATCACC GTGCCGGCGT ACTTCAACGA

LMG 476 AGACCGCCGA GGCGTTCCTG GGCGAGACCG TCACCGAGGC GGTCATCACC GTGCCGGCGT ACTTCAACGA

NCPPB4393 AGACCGCCGA GGCGTTCCTG GGCGAGACCG TCACCGAGGC GGTCATCACC GTGCCGGCGT ACTTCAACGA

R1 AGACCGCCGA GGCGTTCCTG GGCGAGACCG TCACCGAGGC GGTCATCACC GTGCCGGCGT ACTTCAACGA

XCV85-10 AGACCGCCGA AGACTTCCTC GGCGAGAAGG TCACCGAGGC GGTGATCACC GTGCCGGCAT ACTTCAACGA

4341

GPE PC73 TAGCCAGCGT CAGGCGACCA AGGACGCCGG TCGCATCGCC GGCCTGGACG TCAAGCGCAT CATCAACGAG

Xa23R1 TAGCCAGCGT CAGGCGACCA AGGACGCCGG TCGCATCGCT GGCCTGGACG TCAAGCGCAT CATCAACGAG

GPE 39 TAGCCAGCGT CAGGCGACCA AGGACGCCGG TCGTATCGCC GGCTTGGATG TCAAGCGCAT CATCAACGAG

MUS 060 TAGTCAGCGT CAGGCGACCA AGGACGCCGG TCGTATCGCC GGCTTGGATG TCAAGCGCAT CATCAACGAG

LMG 476 CAGCCAGCGC CAGGCGACCA AGGACGCCGG CCGCATCGCC GGCCTGGACG TCAAGCGCAT CATCAACGAG

NCPPB4393 CAGCCAGCGC CAGGCGACCA AGGACGCCGG CCGCATCGCC GGCCTGGACG TCAAGCGCAT CATCAACGAG

R1 CAGCCAGCGC CAGGCGACCA AGGACGCCGG CCGCATCGCC GGCCTGGACG TCAAGCGCAT CATCAACGAG

XCV85-10 CAGCCAGCGT CAGGCCACCA AGGATGCCGG CCGCATCGCC GGTCTGGACG TCAAGCGCAT CATCAACGAG

4411

GPE PC73 CCTACAGCGG CGGCGCTTGC ATATGGCTTG GACAAGGCTC ACGGCGGTGA TCGCAAGATT GCCGTGTACG

Xa23R1 CCTACGGCGG CGGCGCTGGC CTATGGCTTG GACAAGGCTC ACGGCGGTGA TCGCAAGATT GCCGTGTACG

GPE 39 CCGACGGCGG CGGCGCTTGC CTATGGCTTG GACAAGGCGC ACGGCGGCGA CCGCAAGATT GCCGTGTACG

MUS 060 CCAACGGCGG CGGCGCTTGC CTATGGCTTG GACAAGGCGC ACGGCGGCGA CCGCAAGATT GCCGTGTACG

LMG 476 CCGACCGCGG CGGCGTTGGC CTACGGCCTG GACAAGGGCC AGGGCGGCGA CCGCAAGATC GCCGTGTACG

NCPPB4393 CCGACCGCGG CGGCGTTGGC CTACGGCCTG GACAAGGGCC AGGGCGGCGA CCGCAAGATC GCCGTGTACG

R1 CCGACTGCGG CTGCGCTGGC CTATGGCCTG GACAAGGGCC AGGGCGGCGA CCGCAAGATC GCCGTGTACG

XCV85-10 CCGACCGCCG CAGCCCTGGC CTATGGCCTG GACAAGA--- ACGGCGGCGA CCGCAAGATA GCCGTGTACG

4481

GPE PC73 ACCTGGGTGG CGGCACCTTC GACGTGTCGA TCATCGAAAT CGCCAATGTC GACGGCGAAA AGCAGTTCGA

Xa23R1 ACCTGGGTGG CGGCACCTTC GACGTGTCGA TCATCGAAAT CGCCAATGTC GACGGCGAGA AGCAGTTCGA

GPE 39 ACCTGGGTGG CGGCACCTTC GACGTGTCGA TCATCGAAAT CGCCAATGTC GACGGCGAAA AGCAGTTCGA

MUS 060 ACCTGGGTGG CGGCACCTTC GACGTGTCGA TCATCGAAAT CGCCAATGTC GACGGCGAAA AGCAGTTCGA

LMG 476 ACCTGGGCGG CGGCACCTTC GACGTGTCGA TCATCGAGAT CGCCAACGTC GACGGTGAGA AGCAGTTCGA

NCPPB4393 ACCTGGGCGG CGGCACCTTC GACGTGTCGA TCATCGAGAT CGCCAACGTC GACGGTGAGA AGCAGTTCGA

R1 ACCTGGGCGG CGGCACCTTC GACGTGTCGA TCATCGAGAT CGCCAATGTC GACGGCGAGA AGCAGTTCGA

XCV85-10 ACCTGGGCGG CGGTACCTTC GACGTGTCGA TTATCGAGAT CGCCGAAGTC GATGGTGAAA AGCAGTTCGA

4551

GPE PC73 AGTGCTAGCC ACCAATGGCG ACACCTTCCT GGGTGGCGAG GATTTCGACA AGCGCGTCAT CGACTATCTG

Xa23R1 AGTGCTGGCC ACCAATGGCG ACACCTTCCT GGGTGGCGAG GATTTCGACA AGCGCGTCAT CGACTATCTG

GPE 39 AGTACTGGCC ACCAACGGCG ACACCTTCCT GGGTGGCGAA GATTTCGACA AGCGCGTCAT CGACTATCTG

MUS 060 AGTACTGGCC ACCAACGGCG ACACCTTCCT GGGTGGCGAA GATTTCGACA AGCGCGTCAT CGACTATCTG

LMG 476 AGTGCTGGCC ACCAACGGCG ACACCTTCTT GGGCGGCGAA GACTTCGACA AGCGCGTCAT CGACTACCTC

NCPPB4393 AGTGCTGGCC ACCAACGGCG ACACCTTCTT GGGCGGCGAA GACTTCGACA AGCGCGTCAT CGACTACCTC

R1 AGTGCTGGCC ACCAACGGCG ACACCTTCCT GGGCGGCGAA GACTTCGACA AGCGCGTCAT CGACTACTTG

XCV85-10 AGTGCTGGCC ACCAACGGCG ACACCTTCCT GGGCGGCGAA GACTTCGACA ACCGCGTCAT CGAGTACCTG

4621

GPE PC73 GTGGACGAGT TCAACAAAGA TCAAGGCATC GATCTGCGCA AGGATCCGTT GGCGCTACAG CGCCTGAAGG

Xa23R1 GTGGACGAGT TCAACAAAGA TCAAGGCATC GATCTGCGCA AGGATCCGTT GGCGCTACAG CGCCTGAAGG

GPE 39 GTGGACGAGT TCAACAAGGA TCAGGGAATC GACCTGCGCA AGGATCCGTT GGCGCTACAG CGTCTGAAGG

MUS 060 GTGGACGAGT TCAACAAGGA TCAGGGAATC GACCTGCGCA AGGATCCGTT GGCGCTACAG CGTCTGAAGG

LMG 476 GTCGAAGAGT TCAACAAGGA CCAGGGCATC GACCTGCGCA AGGATCCGCT GGCGCTGCAG CGCCTGAAGG

NCPPB4393 GTCGAAGAGT TCAACAAGGA CCAGGGCATC GACCTGCGCA AGGATCCGCT GGCGCTGCAG CGCCTGAAGG

R1 GTGGAAGAGT TCAACAAGGA CCAGGGCATC GACCTGCGCA AGGATCCGCT GGCGCTGCAG CGCCTGAAGG

XCV85-10 GTCGACGAAT TCAACAAGGA CCAGGGCATC GACCTGCGTA AGGATCCGCT GGCGCTGCAG CGCCTGAAGG

4691

GPE PC73 ATGCCGCCGA GCGTGCCAAG ATCGAACTCT CGTCCTCGCA GCAGACCGAA GTCAACTTGC CTTACGTCAC

Xa23R1 ATGCCGCCGA GCGTGCCAAG ATCGAACTCT CGTCCTCGCA GCAGACCGAA GTCAACTTGC CTTACGTCAC

GPE 39 ATGCCGCCGA GCGTGCCAAG ATCGAGCTGT CGTCCTCGCA GCAGACCGAA GTCAACCTGC CTTACGTCAC

MUS 060 ATGCCGCCGA GCGTGCCAAG ATCGAGCTGT CGTCCTCGCA GCAGACCGAA GTCAACCTGC CTTACGTCAC

LMG 476 ATGCCGCCGA GCGCGCCAAG ATCGAGCTGT CGTCCTCGCA GCAGACCGAA GTCAACCTGC CGTACGTCAC

NCPPB4393 ATGCCGCCGA GCGCGCCAAG ATCGAGCTGT CGTCCTCGCA GCAGACCGAA GTCAACCTGC CGTACGTCAC

R1 ATGCCGCCGA GCGCGCCAAG ATCGAGTTGT CGTCCTCGCA GCAGACCGAA GTCAACCTGC CGTACGTCAC

XCV85-10 ACGCCGCAGA GCGCGCCAAG ATCGAGCTGT CGTCCAGCCA GCAGACCGAA GTGAACCTGC CGTACGTCAC

4761

GPE PC73 CGCCGACGCG TCGGGTCCGA AGCATCTGAA CATCAAGCTG ACCCGGGCCA AGCTCGAGGC GCTGGTGGAA

Xa23R1 CGCCGACGCG TCGGGTCCGA AGCATCTGAA CATCAAGCTG ACCCGGGCCA AGCTCGAGGC GCTGGTGGAA

GPE 39 CGCCGACGCG TCTGGCCCGA AGCATCTGAA CATCAAGCTG ACCCGGGCCA AGCTCGAAGC GCTGGTGGAA

MUS 060 CGCCGACGCG TCTGGCCCGA AGCATCTGAA CATCAAGCTG ACCCGGGCCA AGCTCGAAGC GCTGGTGGAA

LMG 476 CGCCGACGCG TCGGGCCCGA AGCACCTCAA CATCAAGCTG ACCCGGGCCA AGCTCGAGGC GCTGGTGGAG

NCPPB4393 CGCCGACGCG TCGGGCCCGA AGCACCTCAA CATCAAGCTG ACCCGGGCCA AGCTCGAGGC GCTGGTGGAG

R1 CGCCGACGCG TCGGGCCCGA AGCACCTCAA CATCAAGCTG ACCCGGGCCA AGCTCGAGGC GCTGGTGGAG

XCV85-10 CGCCGATGCG TCGGGCCCGA AGCACCTCAA CATCAAGTTG ACCCGTGCCA AGCTCGAAGC GCTGGTGGAA

4831

GPE PC73 GATCTGGTCA AGCGCACCAT CGACCCGTGC CGCACCGCGT TGAACGATGC CGGCTTGCGC GCCAGCGACA

Xa23R1 GATCTGGTCA AGCGCACCAT CGATCCGTGC CGCACCGCGT TGAACGATGC CGGCTTGCGC GCCAGCGACA

GPE 39 GATCTGGTCA AGCGCACCAT CGATCCGTGC CGCACCGCGC TGAACGACGC CGGTCTGCGC GCCAGCGACA

MUS 060 GATCTGGTCA AGCGCACCAT CGATCCGTGC CGCACCGCGC TGAACGACGC CGGCCTGCGC GCCAGCGACA

LMG 476 GACCTGGTCA AGCGCACCAT CGATCCGTGC CGCACCGCGC TGAACGACGC CGGCCTGCGC GCCAGCGACA

NCPPB4393 GACCTGGTCA AGCGCACCAT CGATCCGTGC CGCACCGCGC TGAACGACGC CGGCCTGCGC GCCAGCGACA

R1 GACCTGGTCA AGCGCACCAT CGATCCGTGC CGCACCGCGC TGAACGACGC CGGCCTGCGC GCCAGCGACA

XCV85-10 GACCTGGTCA AGAAGTCGAT CGAACCGTGC CGCACCGCGT TGAACGACGC CGGCCTGCGC GCCAGCGACA

4901

GPE PC73 TCACCGAGGT GATCCTGGTC GGTGGCCAGA CCCGCATGCC CAAGGTGCAG CAGGCCGTGG CCGAGTTCTT

Xa23R1 TCACCGAGGT GATCCTGGTC GGTGGCCAGA CCCGCATGCC CAAGGTGCAG CAGGCCGTGG CCGAGTTCTT

GPE 39 TCACCGAGGT GATCCTGGTC GGTGGGCAGA CCCGTATGCC CAAGGTGCAG CAGGCCGTGG CCGAGTTCTT

MUS 060 TCACCGAGGT GATCCTGGTC GGTGGGCAGA CCCGTATGCC CAAGGTGCAG CAGGCCGTGG CCGAGTTCTT

LMG 476 TCACCGAGGT GATCCTGGTC GGTGGCCAGA CCCGCATGCC GAAGGTGCAG CAGGCGGTGG CCGAGTTCTT

NCPPB4393 TCACCGAGGT GATCCTGGTC GGTGGCCAGA CCCGCATGCC GAAGGTGCAG CAGGCGGTGG CCGAGTTCTT

R1 TCACCGAGGT GATCCTGGTC GGTGGCCAGA CCCGCATGCC GAAGGTGCAG CAGGCGGTGG CCGAGTTCTT

XCV85-10 TCAACGAAGT GATCCTGGTC GGCGGCCAGA CCCGCATGCC GAAGGTGCAG CAGGCGGTTG CCGATTTCTT

4971

GPE PC73 CGGCAAGGAT CCGCGCAAGG ACGTCAACCC GGACGAGGCC GTGGCGTTGG GCGCGGCGAT CCAGGGCGGT

Xa23R1 CGGCAAGGAT CCGCGCAAGG ACGTCAACCC GGACGAGGCC GTGGCGTTGG GCGCGGCGAT CCAGGGCGGT

GPE 39 CGGCAAGGAC CCGCGCAAAG ACGTCAACCC GGATGAGGCG GTGGCGTTGG GCGCGGCGAT CCAGGGCGGT

MUS 060 CGGCAAGGAC CCGCGCAAAG ACGTCAACCC GGATGAGGCT GTGGCATTGG GCGCGGCGAT CCAGGGCGGT

LMG 476 CGGCAAGGAG CCGCGCAAGG ACGTCAACCC CGACGAGGCC GTGGCGCTGG GCGCGGCGAT CCAGGGCGGC

NCPPB4393 CGGCAAGGAG CCGCGCAAGG ACGTCAACCC CGACGAGGCC GTGGCGCTGG GCGCGGCGAT CCAGGGCGGC

R1 CGGCAAGGAG CCGCGCAAGG ACGTCAACCC CGACGAGGCC GTGGCGCTGG GCGCCGCGAT CCAGGGCGGC

XCV85-10 CGGCAAGGAA CCGCGCAAGG ACGTCAACCC GGACGAAGCG GTGGCCGTGG GTGCGGCGAT CCAGGGCGGC

5041

GPE PC73 GTGCTGGCCG GCGACGTCAA GGACGTCTTG CTGCTCGACG TGACCCCGCT GAGCCTGGGG ATCGAGACCC

Xa23R1 GTGCTGGCCG GCGACGTCAA GGACGTCTTG CTGCTCGACG TGACGCCGCT GAGCCTGGGG ATCGAGACCC

GPE 39 GTGCTGGCCG GCGACGTCAA GGACGTCTTG CTGCTCGACG TGACCCCGCT GAGCCTGGGA ATCGAGACCC

MUS 060 GTGCTGGCCG GCGACGTCAA GGACGTCTTG CTGCTCGACG TGACCCCGCT GAGCCTGGGA ATCGAGACCC

LMG 476 GTGCTGGCCG GCGACGTCAA GGACGTGCTG CTGCTCGACG TGACCCCGCT GAGCCTGGGC ATCGAGACCC

NCPPB4393 GTGCTGGCCG GCGACGTCAA GGACGTGCTG CTGCTCGACG TGACCCCGCT GAGCCTGGGC ATCGAGACCC

R1 GTGCTGGCCG GCGACGTCAA GGACGTGCTG CTGCTCGACG TGACCCCGCT GAGCCTGGGC ATCGAGACCC

XCV85-10 GTGCTGGCCG GCGACGTCAA GGACGTGCTG CTGCTGGACG TGACCCCGCT GTCGCTGGGT ATCGAGACCA

5111

GPE PC73 TGGGTGGCGT GTTCACCAAG ATCATCGAGA AGAACACCAC CATCCCGACC AAGGCCGCGC AGGTGTTCTC

Xa23R1 TGGGTGGCGT GTTCACCAAG ATCATCGAGA AGAACACCAC CATCCCGACC AAGGCCGCGC AGGTGTTCTC

GPE 39 TGGGCGGCGT GTTCACCAAG ATCATCGAGA AGAACACCAC CATCCCGACC AAGGCCGCGC AGGTGTTTTC

MUS 060 TGGGCGGCGT GTTCACCAAG ATCATCGAGA AGAACACCAC CATCCCGACC AAGGCCGCGC AGGTGTTCTC

LMG 476 TGGGCGGCGT GTTCACCAAG ATCATCGAGA AGAACACCAC CATCCCGACC AAGGCCTCGC AGGTGTTCTC

NCPPB4393 TGGGCGGCGT GTTCACCAAG ATCATCGAGA AGAACACCAC CATCCCGACC AAGGCCTCGC AGGTGTTCTC

R1 TGGGCGGCGT GTTCACCAAG ATCATCGAGA AGAACACCAC CATCCCGACC AAGGCCTCGC AGGTGTTCTC

XCV85-10 TGGGCGGCGT GTTCACCAAG ATCATCGAAA AGAACACCAC CATTCCGACC AAGGCCTCGC AGACCTTCTC

5181

GPE PC73 CACTGCCGAG GACAATCAGT CGGCAGTGAC CGTGCATGTG CTGCAGGGCG AGCGCGAACA GGCTCGCTAT

Xa23R1 CACTGCCGAG GACAATCAGT CGGCAGTGAC CGTGCATGTG CTGCAGGGCG AGCGCGAACA GGCTCGCTAT

GPE 39 CACCGCCGAT GACAATCAGT CGGCAGTGAC CGTGCATGTG TTGCAGGGCG AACGCGAACA GGCTCGCTAC

MUS 060 CACCGCCGAG GACAATCAGT CGGCGGTGAC CGTGCATGTG TTGCAGGGCG AGCGCGAACA GGCTCGCTAC

LMG 476 CACCGCCGAG GACAACCAGT CGGCGGTGAC CGTGCACGTG CTGCAGGGCG AGCGCGAACA GGCCCGCTTC

NCPPB4393 CACCGCCGAG GACAACCAGT CGGCGGTGAC CGTGCACGTG CTGCAGGGCG AGCGCGAACA GGCCCGCTTC

R1 CACCGCCGAG GACAACCAGT CGGCGGTGAC CGTGCACGTG CTGCAGGGCG AGCGCGAGCA GGCCCGCTAC

XCV85-10 CACCGCCGAA GACAACCAGT CGGCCGTGAC CGTGCATGTG TTGCAGGGTG AGCGCGAGCA GGCCCGCTTC

5251

GPE PC73 AACAAGTCTT TGGCCAAGTT TGATCTGTCC GGCATTGAGC CGGCGCCGCG TGGCCTGCCG CAGGTGGAGG

Xa23R1 AACAAGTCTT TGGCCAAGTT TGATCTGTCC GGCATTGAGC CGGCGCCGCG TGGCCTGCCG CAAGTGGAGG

GPE 39 AACAAGTCCT TGGCCAAGTT CGATCTGTCC GGCATCGAAC CGGCGCCACG CGGCCTGCCG CAGGTGGAGG

MUS 060 AACAAGTCCT TGGCCAAGTT CGATCTGTCC GGCATCGAAC CGGCGCCACG CGGCTTGCCG CAGGTGGAGG

LMG 476 AACAAGTCGC TGGCCAAGTT CGACCTGTCC GGCATCGAGC CGGCGCCGCG CGGCCTGCCG CAGGTGGAGG

NCPPB4393 AACAAGTCGC TGGCCAAGTT CGACCTGTCC GGCATCGAGC CGGCGCCGCG CGGCCTGCCG CAGGTGGAGG

R1 AACAAGTCGC TGGCCAAGTT CGACCTGTCC GGCATCGAGC CGGCGCCGCG CGGCCTGCCG CAGGTGGAGG

XCV85-10 AACAAGTCGC TGGCCAAGTT CGACCTGTCC GGCATCGAGC CGGCGCCGCG CGGCATGCCG CAGGTGGAAG

5321

GPE PC73 TGTCCTTCGA CATCGACGCC AACGGCATCT TGCACGTGTC GGCCAAGGAC AAGAAGACCA ACAAGGAACA

Xa23R1 TGTCCTTCGA CATCGACGCC AACGGCATCT TGCACGTGTC GGCCAAGGAC AAGAAGACCA ACAAGGAACA

GPE 39 TGTCCTTCGA CATCGACGCC AACGGCATCC TGCACGTGTC GGCCAAAGAC AAGAAGACCA ACAAGGAACA

MUS 060 TGTCCTTCGA CATCGACGCC AACGGCATCC TGCACGTGTC GGCCAAAGAC AAGAAGACCA ACAAGGAACA

LMG 476 TGTCCTTCGA CATCGACGCC AACGGCATCC TGCACGTGTC GGCCAAGGAC AAGAAGACCA ACAAGGAACA

NCPPB4393 TGTCCTTCGA CATCGACGCC AACGGCATCC TGCACGTGTC GGCCAAGGAC AAGAAGACCA ACAAGGAACA

R1 TGTCCTTCGA CATCGACGCC AACGGCATTC TGCACGTGTC GGCCAAGGAC AAGAAGACCA ACAAGGAACA

XCV85-10 TGTCCTTCGA CATCGACGCC AACGGCATCC TGCACGTGTC GGCCAAGGAC AAGAAGACCA ACAAGGAACA

5391

GPE PC73 GAAGGTCGAG ATCAAGGCCG GGTCGGGTCT GTCGGACGCG GAAATCCAGC GCATGGTCGC CGATGCCGAA

Xa23R1 GAAGGTCGAG ATCAAGGCCG GGTCGGGTCT GTCGGACGCG GAAATCCAGC GCATGGTCGC CGATGCCGAA

GPE 39 GAAGGTCGAG ATCAAGGCTG GCTCGGGCCT GTCAGATGCG GAAATCCAGC GGATGGTCGC CGACGCCGAA

MUS 060 GAAGGTCGAG ATCAAGGCTG GTTCGGGCCT GTCGGATGCG GAAATCCAGC GGATGGTCGC CGACGCTGAA

LMG 476 GAAGGTCGAG ATCAAGGCCG GTTCCGGCCT GTCGGACGAC GAGATCCAGC GGATGGTCGC CGACGCGGAA

NCPPB4393 GAAGGTCGAG ATCAAGGCCG GTTCCGGCCT GTCGGACGAC GAGATCCAGC GGATGGTCGC CGACGCGGAA

R1 GAAGGTCGAG ATCAAGGCCG GCTCCGGTCT GTCGGACGAC GAGATCCAGC GGATGGTCGC CGACGCGGAA

XCV85-10 GAAGGTCGAG ATCAAGGCCG GTTCGGGTCT GTCGGACGAA GAGATCCAGC GCATGGTCGC CGACGCGGAA

5461

GPE PC73 GCCAATCGCG AGGAAGACAA GAAGTTCCAC GAGTTGGTGC AGGCGCGCAA CCAGGCCGAT GGCTTGATCC

Xa23R1 GCCAATCGCG AGGAAGACAA GAAGTTCCAC GAGTTGGTGC AGGCGCGCAA CCAGGCCGAT GGCCTGATCC

GPE 39 GCCAACCGCG AGGAAGACAA GAAGTTCCAC GAGTTGGTGC AGGCGCGCAA CCAGGCCGAC GGCTTGATCC

MUS 060 GCCAACCGCG AGGAAGACAA GAAGTTCCAC GAGTTGGTGC AGGCGCGCAA CCAGGCCGAC GGCTTGATCC

LMG 476 GCCAACCGCG AGGAAGACAA GAAGTTCCAC GAGCTGGTGC AGGCGCGCAA CCAGGCCGAC GGCCTGATCC

NCPPB4393 GCCAACCGCG AGGAAGACAA GAAGTTCCAC GAGCTGGTGC AGGCGCGCAA CCAGGCCGAC GGCCTGATCC

R1 GCCAACCGCG AGGAAGACAA GAAGTTCCAC GAGCTGGTGC AGGCGCGCAA CCAGGCCGAC GGCCTGATCC

XCV85-10 GCCAACCGCG AAGAAGACAA AAAGTTCCAG GAGCTGGTGC AGGCCCGCAA CCAGGCCGAT GGTCTGATCC

5531

GPE PC73 ACTCCACGCG CAGCGCGATC GCCGAGCACG GCAGCAAGGT TGGCGGCGAC GTGATTGGCA AGGTCGAAGC

Xa23R1 ACTCCACGCG CAGCGCGATC GCCGAGCACG GCAGCAAGGT TGGCGGCGAC GTGATTGGCA AGGTCGAAGC

GPE 39 ACTCCACGCG CAGCGCGATC ACCGAGCATG GCAGCAAGGT CGGCGGTGAC GTGATCGGTA AGGTCGAAGC

MUS 060 ACTCCACGCG CAGCGCGATC ACCGAGCACG GCAGCAAGGT CGGCGGTGAC GTGATCGGTA AGGTCGAAGC

LMG 476 ACGCCACCCG CAGCGCGATC ACCGAGCACG GCAGCAAGGT CGGCGGCGAC GTGATCGGCA AGGTCGAGTC

NCPPB4393 ACGCCACCCG CAGCGCGATC ACCGAGCACG GCAGCAAGGT CGGCGGCGAC GTGATCGGCA AGGTCGAGTC

R1 ACGCCACCCG TAGCGCGATC ACCGAGCACG GCAGCAAGGT CGGCGGCGAT GTGATCGGCA AGGTCGAGTC

XCV85-10 ACGCCACCCG TACCGCGATC ACCGAGCATG GCAGCAAGGT CGGTGGCGAT GTGATCGGCA AGGTGGAAGC

5601

GPE PC73 CGCACTGGCC GATCTTGAAA CCGCGATGAA GGGCGAGGAC AAGAGCCAGA TTGAGGCCAA GAGCAAAACG

Xa23R1 CGCACTGGCC GATCTGGAAA CCGCGATGAA GGGCGAGGAC AAGAGCCAGA TTGAGGCCAA GAGCAAAACG

GPE 39 CGCACTGGCG GATTTGGAAA CCGCGATGAA GGGCGAGGAC AAGAGCCAGA TCGAGGCCAA GACCAAAACC

MUS 060 CGCACTGGCC GATTTGGAAA CCGCGATGAA GGGCGAGGAC AAGAGCCAGA TCGAGGCCAA GACCAAAACG

LMG 476 GGCGCTGGCG GACCTGGAAA CCGCGATGAA GGGCGACGAC AAGGGCCAGA TCGAGGCCAA GACCAAGGCG

NCPPB4393 GGCGCTGGCG GACCTGGAAA CCGCGATGAA GGGCGACGAC AAGGGCCAGA TCGAGGCCAA GACCAAGGCG

R1 GGCGCTGGCG GATCTGGAAA CCGCGATGAA GGGCGACGAC AAGGGCCAGA TCGAGGCCAA GACCAAGGCG

XCV85-10 GGCGCTGTCG GATCTGGAAA CCGCCATGAA GGGCGACGAC AAGGCGCAGA TCGAAGCACG CACCAAGACG

5671

GPE PC73 TTGGAGGAGG TCGGCCAGTC GTTGTACGCG GCTGCGGCGG CC---GGCGA GCAGCCGCCG GCCGCCAGTG

Xa23R1 TTGGAGGAGG TCGGCCAGTC GTTGTACGCG GCTGCGGCGG CC---GGCGA GCAGCCGCCG GGCGCCAGTG

GPE 39 TTGGAGGAGG TCGGCCAGTC GTTGTACGCG GCGGCCGCGG CCGGTGGCGA GCAGCCGGCC GGTGCCAGTG

MUS 060 TTGGAGGAGG TCGGCCAGTC GTTGTACGCG GCGGCGGCGG CCGGTGGCGA GCAGCCGGCC GGTGCCAGTG

LMG 476 CTGGAAGAGG CCGGTCAGTC GCTGTACGCG GCCGCGGCGG CG---GGCGA GCAGCAGCCG GGCGGCAACG

NCPPB4393 CTGGAAGAGG CCGGTCAGTC GCTGTACGCG GCCGCGGCGG CG---GGCGA GCAGCAGCCG GGCGGCAACG

R1 CTGGAAGAGG CCGGTCAGTC GCTGTACGCG GCCGCGGCGG CG---GGCGA GCAGCAGCCG GGCGGCAACG

XCV85-10 CTGGAAGAAG CCGGTCAGTC GTTGTACGCA GCAGCTGCGG CG---GCAGA GCAGGGCGGT AACGCCGATG

5741

GPE PC73 C------GGG TGGCGCGCAT GCGTCCTCG- --TCGGCCGA TGACGTGGTC GATGCCGAGT TTACCGAAGT

Xa23R1 C------GGG TGGTGCGCAT GCGTCCTCG- --TCGGCCGA TGACGTGGTC GATGCCGAGT TTACCGAAGT

GPE 39 C------CGG TGGCGCGCAC GCGTCCTCG- --TCGGCCGA CGACGTGGTC GACGCCGAGT TCACCGAAGT

MUS 060 C------CGG TGGCGCGCAC GCGTCCTCG- --TCGGCCGA CGACGTGGTC GACGCTGAGT TCACCGAAGT

LMG 476 C------CGG CGGCGCGCAT GCCTCCTCGG CCTCGTCCGA CGACGTGGTC GACGCCGAGT TCACCGAGGT

NCPPB4393 C------CGG CGGCGCGCAT GCCTCCTCGG CCTCGTCCGA CGACGTGGTC GACGCCGAGT TCACCGAGGT

R1 C------CGG CGGCGCGCAT GCGTCCTCGG CCCAGTCCGA CGACGTGGTC GACGCCGAGT TCACCGAAGT

XCV85-10 CGGCCAGCGG CAACGCCCAG GCCTCCAAG- --GCCGCCGA CGACGTGGTG GACGCCGAGT TCACCGAGGT

5811

GPE PC73 CAAGGACGGC AAGAAGTAAA TGGCCAGCTA TGGCATGAAC GATGTCAAGA ACGGGATGAA GATCCTGGTC

Xa23R1 CAAGGACGGC AAGAAGTAAA TGGCCAGCTA TGGCATGAAC GATGTCAAGA ACGGGATGAA GATCCTGGTC

GPE 39 CAAGGACGGC AAGAAGTAAA TGGCCAGCTA TGGCATGAAC GATGTCAAGA ACGGGATGAA AATCCTGGTC

MUS 060 CAAGGACGGC AAGAAGTAAA TGGCCAGCTA TGGCATGAAC GATGTCAAGA ACGGGATGAA AATCCTGGTC

LMG 476 CAAGGACGAC AAGAAGTAAA TGGCCAGCTA CGGCATGAAC GACGTCAAGA ACGGGATGAA GATCCTGGTC

NCPPB4393 CAAGGACGAC AAGAAGTAAA TGGCCAGCTA CGGCATGAAC GACGTCAAGA ACGGGATGAA GATCCTGGTC

R1 CAAGGACGAC AAGAAGTAAA TGGCCAGCTA CGGCATGAAC GACGTCAAGA ACGGGATGAA GATCCTGGTC

XCV85-10 CAAGGACGAC AAGAAGTAAA TGGCCACTGT TGGCATGAAC GACGTCAAGA ACGGCATGAA GATCCTGGTC

5881

GPE PC73 AACAGCGAAC CGGCGATCAT CACCGATACC GAATACGTGA AGCCGGGCAA GGGCCAGGCC TTCACCCGGG

Xa23R1 AACAGCGAAC CGGCGATCAT CACCGATACC GAATACGTGA AGCCGGGCAA GGGCCAGGCC TTCACCCGGG

GPE 39 AACAGCGAAC CTGCGATCAT CACCGATACC GAATACGTGA AGCCGGGCAA GGGCCAGGCG TTCACCCGGG

MUS 060 AACAGCGAAC CTGCGATCAT CACCGATACC GAATACGTGA AGCCGGGCAA GGGCCAGGCG TTCACCCGGG

LMG 476 AACAGCGAGC CTGCGATCAT CACCGATACC GAATACGTCA AGCCCGGCAA GGGCCAGGCG TTCACCCGCG

NCPPB4393 AACAGCGAGC CTGCGATCAT CACCGATACC GAATACGTCA AGCCCGGCAA GGGCCAGGCG TTCACCCGCG

R1 AACAGCGAGC CTGCGATCAT CACCGATACC GAATACGTCA AGCCCGGCAA GGGCCAGGCG TTCACCCGCG

XCV85-10 AACAACGAGC CGGCGGTCAT CACCGAGACC GAATACGTCA AGCCGGGCAA GGGCCAGGCC TTCACCCGCA

5951

GPE PC73 TCAAATATCG TTTCATCAAA TCCGGGCGCG TGGTCGAAAT GACCATGAAG GCCACCGACA GCGTGGAAGC

Xa23R1 TCAAATATCG TTTCATCAAA TCCGGGCGCG TGGTCGAAAT GACCATGAAG GCCACCGACA GCGTGGAAGC

GPE 39 TCAAATATCG CTTCATCAAG TCCGGGCGCG TGGTCGAAAT GACCATGAAG GCCACCGACA ACGTGGAAGC

MUS 060 TCAAATATCG CTTCATCAAG TCCGGGCGCG TGGTCGAAAT GACCATGAAG GCCACCGACA ACGTGGAAGC

LMG 476 TCAAGTACCG CTTCATCAAG TCCGGGCGCG TGGTCGAAAT GACCATGAAG GCGACCGACA GCGTGGAAGC

NCPPB4393 TCAAGTACCG CTTCATCAAG TCCGGGCGCG TGGTCGAAAT GACCATGAAG GCGACCGACA GCGTGGAAGC

R1 TCAAGTACCG CTTCATCAAG TCCGGGCGCG TGGTCGAAAT GACCATGAAG GCGACCGACA GCGTGGAAGC

XCV85-10 TGAAGTACCG CTTCATCAAG TCCGGTCGCG TGGTCGAAAT GACCATGAAG GCGACCGACG ATGTGGAAGT

6021

GPE PC73 GGCCGATGTG GTCGATACCA ACATGCAGTA TCTGTATACC GATGGCGAAT ACTGGCACTT CATGCAGCAG

Xa23R1 GGCCGATGTG GTCGATACCA ACATGCAGTA TCTGTATACC GATGGCGAAT ACTGGCACTT CATGCAGCAG

GPE 39 GGCCGACGTG GTCGATACCA ACATGCAGTA CCTGTACACC GACGGCGAAT ACTGGCACTT CATGCAGCAG

MUS 060 CGCCGACGTG GTCGATACCA ACATGCAGTA CCTGTACACC GACGGCGAAT ACTGGCACTT CATGCAGCAG

LMG 476 GGCCGATGTG GTCGACACCG ACATGCAGTA CCTGTACAGC GACGGCGAGT ACTGGCACTT CATGCAGCAG

NCPPB4393 GGCCGATGTG GTCGACACCG ACATGCAGTA CCTGTACAGC GACGGCGAGT ACTGGCACTT CATGCAGCAG

R1 GGCCGACGTG GTCGACACCG ACATGCAGTA CCTGTACAGC GACGGCGAGT ACTGGCACTT CATGCAGCAG

XCV85-10 GGCCGACGTG GTCGATACCG ACATGCGCTA CCTCTACAGC GATGGCGAGT ACTGGCACTT CATGGACCCG

6091

GPE PC73 GAGACCTTCG AGCAGGTGCA GGCCGACAAG GCCGGCGTCG GCGATGCCGC CAAATGGATC AAGGGGGAGG

Xa23R1 GAGACCTTCG AGCAGGTGCA GGCCGACAAG GCCGGCGTCG GCGATGCCGC CAAATGGATC AAGGGGGAGG

GPE 39 GAGACCTTCG AGCAGGTGCA GGCCGACAAG ACCGGCGTTG GCGATGCCGC CAAGTGGATC AAGGGCGAGG

MUS 060 GAGACCTTCG AGCAGGTGCA GGCCGACAAG ACCGGCGTCG GCGATGCCGC CAAGTGGATC AAGGGCGAGG

LMG 476 GAAACCTTCG AGCAGGTGCA GGCCGACAAG GCCGGCGTCG GCGACGCCGC CAAGTGGATC AAGGGCGAGG

NCPPB4393 GAAACCTTCG AGCAGGTGCA GGCCGACAAG GCCGGCGTCG GCGACGCCGC CAAGTGGATC AAGGGCGAGG

R1 GAAACCTTCG AGCAGGTGCA GGCCGACAAG GCCGGCGTCG GCGACGCCGC CAAGTGGATC AAGGGCGAGG

XCV85-10 GAAACCTTCG AGCAGGTGCA GACCGACAAG GCCGGCATGG GCGGCGCCGA CAAGTGGCTC AAGGGCGAGG

6161

GPE PC73 AAGACTGCGT GGTGACGTTG TGGAACGGTA CCCCGATCCA GGTCACCCCG CCGAATTTCG TCGAGCTGAA

Xa23R1 AAGACTGCGT GGTGACGTTG TGGAACGGTA CCCCGATCCA GGTCACCCCG CCGAATTTCG TCGAGCTGAA

GPE 39 AGGACTGCGT GGTGACGTTG TGGAACGGGA CCCCGATCCA GGTCACTCCG CCGAATTTCG TCGAGCTGAA

MUS 060 AGGACTGCGT GGTGACGTTG TGGAACGGGA CCCCGATCCA GGTCACCCCG CCGAATTTCG TCGAGCTGAA

LMG 476 AAGATTGCGT GGTGACGCTG TGGAACGGCA CGCCGATCCA GGTCACCCCG CCGAACTTCG TCGAACTGAA

NCPPB4393 AAGATTGCGT GGTGACGCTG TGGAACGGCA CGCCGATCCA GGTCACCCCG CCGAACTTCG TCGAACTGAA

R1 AGGATTGCGT GGTGACGCTG TGGAACGGCA CGCCGATCCA GGTCACCCCG CCGAACTTCG TCGAACTGAA

XCV85-10 AAGACTGCAT CGTGACCTTG TGGAACGGCA CCCCGATCTG GGTGCAGCCG CCGAACTTCG TCGAGCTGAA

6231

GPE PC73 GATCGTCCAG ACCGATCCGG GCGTGCGTGG CGATACCTCT GGCGGCGGCG GCAAGCCGGC CACGCTGGAG

Xa23R1 GATCGTCCAG ACCGATCCGG GCGTGCGTGG CGATACCTCT GGCGGCGGCG GCAAGCCGGC CACGCTGGAG

GPE 39 GATCGTCGAG ACCGATCCAG GTGTGCGTGG CGATACCTCT GGCGGTGGCG GCAAGCCGGC CACGCTGGAG

MUS 060 GATCGTCGAG ACCGATCCAG GCGTGCGTGG CGATACCTCT GGCGGTGGCG GCAAGCCGGC CACGCTGGAG

LMG 476 GATCGTCGAG ACCGACCCGG GCGTGCGTGG CGATACCTCC GGCGGCGGCG GCAAGCCGGC CACCCTGGAG

NCPPB4393 GATCGTCGAG ACCGACCCGG GCGTGCGTGG CGATACCTCC GGCGGCGGCG GCAAGCCGGC CACCCTGGAG

R1 GATCGTCGAG ACCGACCCGG GCGTGCGCGG CGATACCTCC GGCGGCGGCG GCAAGCCGGC GACCCTGGAG

XCV85-10 GATCACCGAG ACCGATCCGG GCGTGCGCGG CGACACCTCC GGCGGCGGCG GCAAGCCGGC CACGCTGGAA

6301

GPE PC73 ACCGGCGCGG TGGTGCGGGT GCCCTTGTTC GTTGGCCAGG ACGAAGTGAT CAAGGTCGAT ACGCGCTCGG

Xa23R1 ACCGGCGCGG TGGTGCGGGT GCCCTTGTTC GTTGGCCAGG ACGAAGTGAT CAAGGTCGAT ACGCGCTCGG

GPE 39 ACTGGCGCGG TGGTGCGCGT GCCCTTGTTC GTCGGCCAGG ACGAAGTGAT CAAGGTCGAT ACGCGTTCGG

MUS 060 ACTGGCGCGG TGGTGCGCGT GCCCTTGTTC GTCGGCCAGG ACGAAGTGAT CAAGGTCGAT ACGCGTTCGG

LMG 476 ACCGGTGCAG TGGTGCGCGT GCCGCTGTTC GTCGGCCAGG AAGAAGTGAT CCGCGTCGAC ACACGTTCGG

NCPPB4393 ACCGGTGCAG TGGTGCGCGT GCCGCTGTTC GTCGGCCAGG AAGAAGTGAT CCGCGTCGAC ACACGTTCGG

R1 ACCGGCGCGG TGGTGCGCGT GCCGCTGTTC GTCGGCCAGG ACGAAGTGAT CCGCGTCGAT ACGCGTTCGG

XCV85-10 ACCGGCGCGG TGGTGCGCGT GCCGCTGTTC GTCAACCAGG ACGAGATCAT CAAGGTCGAT ACCCGTTCGG

6371

GPE PC73 GCGAATATTT CTCGCGCGTC AAGTAAATGG CTGCCAAAGA TATCCGTTTC GGCGAAGACG CCCGTTCGCG

Xa23R1 GCGAATATTT CTCGCGCGTC AAGTAAATGG CTGCCAAAGA TATCCGTTTC GGCGAAGACG CCCGTTCGCG

GPE 39 GCGAATATTT CTCGCGCGTC AAGTAAATGG CTGCCAAAGA TATCCGTTTC GGTGAAGACG CCCGTTCGCG

MUS 060 GCGAGTATTT CTCGCGCGTC AAGTAAATGG CTGCCAAAGA TATCCGTTTC GGTGAAGACG CCCGTTCGCG

LMG 476 GCGAATACGT CTCGCGCGTC AAGTAAATGG CTGCCAAAGA TATCCGTTTC GGTGAAGACG CCCGTTCGCG

NCPPB4393 GCGAATACGT CTCGCGCGTC AAGTAAATGG CTGCCAAAGA TATCCGTTTC GGTGAAGACG CCCGTTCGCG

R1 GCGAGTACGT CTCGCGCGTC AAATAAATGG CTGCCAAAGA TATCCGTTTC GGTGAAGACG CCCGTTCGCG

XCV85-10 GCGAATACTC GGCGCGCGTC AAGTAAATGG CTGCTAAAGA CATTCGTTTC GGTGAAGACG CACGTACCCG

6441

GPE PC73 CATGGTGCGC GGTGTCAACA TCCTCGCCAA TGCCGTCAGT GCCACCCTTG GCCCGAAGGG CCGCAACGTC

Xa23R1 CATGGTGCGC GGTGTCAACA TCCTCGCCAA TGCCGTCAGT GCCACCCTTG GCCCGAAGGG CCGCAACGTC

GPE 39 CATGGTGCGC GGTGTCAATA TCCTCGCCAA TGCCGTCAAG GCGACCCTTG GCCCGAAGGG CCGCAACGTC

MUS 060 CATGGTGCGC GGTGTCAACA TCCTCGCCAA TGCCGTCAAG GCGACCCTTG GCCCGAAGGG CCGCAACGTC

LMG 476 CATGGTGCGC GGCGTCAACA TCCTCGCCAA TGCCGTCAAG GCCACCCTGG GCCCGAAGGG CCGCAACGTC

NCPPB4393 CATGGTGCGC GGCGTCAACA TCCTCGCCAA TGCCGTCAAG GCCACCCTGG GCCCGAAGGG CCGCAACGTC

R1 CATGGTGCGC GGCGTCAACA TCCTCGCCAA TGCCGTCAAG GCCACCCTGG GCCCGAAGGG CCGCAACGTC

XCV85-10 CATGGTTCGT GGCGTCAACG TGCTTGCCAA TGCCGTGAAG GCCACCCTGG GCCCGAAGGG CCGCAACGTC

6511

GPE PC73 GTGCTCGAGA AGAGCTTCGG TGCCCCGCAC ATCACCAAGG ACGGCGTCTC CGTCGCCAAG GAAATCGAAC

Xa23R1 GTGCTCGATA AGAGCTTCGG TGCCCCGCAC ATCACCAAGG ACGGCGTCTC CGTCGCCAAG GAAATCGAAC

GPE 39 GTGCTCGAGA AGAGCTTCGG TGCCCCGACC ATCACCAAGG ACGGCGTCTC CGTTGCCAAG GAAATCGAGC

MUS 060 GTGCTCGAGA AGAGCTTCGG TGCCCCGACC ATCACCAAGG ATGGCGTCTC CGTTGCCAAG GAAATCGAGC

LMG 476 GTGCTCGAGA AGAGCTTCGG CGCCCCGACC ATCACCAAGG ACGGCGTCTC CGTTGCCAAG GAAATCGAGC

NCPPB4393 GTGCTCGAGA AGAGCTTCGG CGCCCCGACC ATCACCAAGG ACGGCGTCTC CGTTGCCAAG GAAATCGAGC

R1 GTGCTCGAGA AGAGCTTCGG CGCCCCGACC ATCACCAAGG ACGGCGTCTC CGTCGCCAAG GAAATCGAAC

XCV85-10 GTGCTCGAGA AGAGCTTCGG CGCGCCGACC ATCACCAAGG ACGGCGTTTC CGTCGCCAAG GAAATCGAAC

6581

GPE PC73 TGGCGGATAA GTTCGAAAAT ATGGGCGCAC AGATGGTGAA GGAAGTCGCC TCCAAAACGT CCGATAACGC

Xa23R1 TGGCGGATAA GTTCGAGAAT ATGGGCGCAC AGATGGTGAA GGAAGTCGCC TCCAAAACGT CCGATAACGC

GPE 39 TGGCGGACAA GTTCGAGAAC ATGGGCGCGC AGATGGTGAA GGAAGTCGCC TCCAAGACCT CCGACAACGC

MUS 060 TGGCGGACAA GTTCGAGAAC ATGGGCGCGC AGATGGTGAA GGAAGTCGCC TCCAAGACCT CCGACAACGC

LMG 476 TGGCCGACAA GTTCGAGAAC ATGGGCGCGC AGATGGTGAA GGAAGTCGCG TCCAAGACCT CCGACAACGC

NCPPB4393 TGGCCGACAA GTTCGAGAAC ATGGGCGCGC AGATGGTGAA GGAAGTCGCG TCCAAGACCT CCGACAACGC

R1 TGGCCGACAA GTTCGAGAAC ATGGGCGCGC AGATGGTGAA GGAAGTTGCG TCCAAGACCT CCGACAACGC

XCV85-10 TGGCCGACAA GTTCGAGAAC ATGGGCGCGC AGATGGTCAA GGAAGTCGCG TCCAAGACCA ACGACAACGC

6651

GPE PC73 CGGCGATGGC ACCACCACCG CCACCGTGCT GGCGCAGGCG CTGATCCGCG AAGGCTCCAA GGCCGTGGCT

Xa23R1 CGGCGACGGC ACCACCACCG CCACCGTGCT GGCGCAGGCG CTGATCCGCG AAGGCTCCAA GGCCGTGGCT

GPE 39 CGGCGACGGC ACCACCACCG CCACCGTGCT GGCGCAGGCG CTGATCCGCG AAGGCTCCAA GGCCGTCGCT

MUS 060 CGGCGACGGC ACCACCACCG CCACCGTGTT GGCGCAGGCG CTGATCCGCG AAGGCTCCAA GGCCGTCGCT

LMG 476 CGGCGACGGC ACCACCACCG CCACCGTGCT GGCGCAGGCG CTGATCCGCG AAGGTTCCAA GGCGGTAGCC

NCPPB4393 CGGCGACGGC ACCACCACCG CCACCGTGCT GGCGCAGGCG CTGATCCGCG AAGGTTCCAA GGCGGTAGCC

R1 CGGCGATGGC ACCACCACCG CCACCGTGCT GGCGCAGGCG CTGATCCGCG AAGGTTCCAA GGCGGTCGCC

XCV85-10 CGGCGACGGC ACCACCACCG CCACCGTGCT GGCCCAGGCC CTGATCCGCG AAGGCGCCAA GGCCGTGGCC

6721

GPE PC73 GCCGGCATGA ACCCGATGGA CCTCAAGCGC GGCATCGACA AGGCGGTTGC GGCCGCCGTG GTCGAGTTGA

Xa23R1 GCCGGCATGA ACCCGATGGA CCTCAAGCGC GGCATCGACA AGGCGGTTGC GGCCGCCGTG GTCGAGTTGG

GPE 39 GCCGGCATGA ACCCGATGGA CCTCAAGCGC GGTATCGACA AGGCCGTTGA GGCTGCCGTG GCTGAACTGA

MUS 060 GCCGGCATGA ACCCGATGGA CCTCAAGCGC GGTATCGACA AGGCCGTCGA GGCCGCCGTG GCCGAACTGA

LMG 476 GCCGGCATGA ATCCGATGGA CCTCAAGCGC GGCATCGACA AGGCCGTCGT GGCCGCCGTG GCCGAGCTGA

NCPPB4393 GCCGGCATGA ATCCGATGGA CCTCAAGCGC GGCATCGACA AGGCCGTCGT GGCCGCCGTG GCCGAGCTGA

R1 GCCGGCATGA ACCCGATGGA CCTCAAGCGC GGCATCGACA AGGCCGTCGT GGCCGCCGTG GCCGAGCTGA

XCV85-10 GCCGGCATGA ACCCGATGGA CCTCAAGCGT GGTATCGACC AGGCCGTCAA GGCTGCGGTC GTCGAGCTGA

6791

GPE PC73 AGAAGATCAG CAAGCCCACC GCCGACGACA AGGCCATCGC CCAGGTCGGC ACCATCTCCG CCAACTCCGA

Xa23R1 AGAAGATCAG CAAGCCCGCC GATGGCGACG AGGGGATCGC CCAGGTCGGC ACCATCTCCG CCAACTCCGA

GPE 39 AGAAGATCAG CAAGCCCACC GCCGATGACA AGGCCATCGC TCAGGTCGGC ACCATTTCGG CCAACTCCGA

MUS 060 AGAAGATCAG CAAGCCCACC GCCGACGACA ACGCCATCGC CCAGGTCGGC ACCATTTCGG CCAACTCCGA

LMG 476 AGAAGATCAG CAAGCCCACC GCCGACGACA AGGCCATCGC CCAGGTCGGC ACCATCTCCG CCAACTCCGA

NCPPB4393 AGAAGATCAG CAAGCCCACC GCCGACGACA AGGCCATCGC CCAGGTCGGC ACCATCTCCG CCAACTCCGA

R1 AGAAGATCAG CAAGCCCACC GCCGACGACA AGGCCATCGC CCAGGTCGGC ACCATCTCCG CCAACTCCGA

XCV85-10 AGAACATCTC CAAGCCCACC ACCGACGACA AGGCGATTGC CCAGGTCGGC ACCATTTCGG CCAACTCGGA

6861

GPE PC73 CGCGTCGATC GGCGACATCA TCGCCGATGC GATGAAGAAG GTCGGCAAGA AGGGTGTGAT CACGGTCGAG

Xa23R1 TAAGGAGATC GGCAGGATCA TCGCCGATGC GATGAAGAAG GTCGGCAAGA AGGGCGTGAT CACGGTCGAG

GPE 39 TAAGGCGATC GGCGACATCA TCGCCGATGC GATGAAGAAG GTCGGCAAGA AGGGCGTGAT CACGGTCGAG

MUS 060 TAAGGCGATC GGCAACATCA TCGCCGACGC GATGAAGAAG GTCGGCAAGA AGGGCGTGAT CACGGTCGAG

LMG 476 CGAGTCGATC GGCAACATCA TCGCCGACGC GATGAAGAAG GTCGGCAAGG AAGGCGTGAT CACCGTCGAG

NCPPB4393 CGAGTCGATC GGCAACATCA TCGCCGACGC GATGAAGAAG GTCGGCAAGG AAGGCGTGAT CACCGTCGAG

R1 CGAGTCGATC GGCAACATCA TCGCCGACGC GATGAAGAAG GTCGGCAAGG AAGGCGTGAT CACGGTCGAG

XCV85-10 CGAGTCGATC GGCAACATCA TTGCCGAAGC GATGAAGAAG GTCGGCAAGG AAGGCGTGAT CACCGTTGAA

6931

GPE PC73 GAAGGCTCGG GCCTGGTCAA CGAACTGGAT GTGGTCGAAG GCATGCAGTT CGACCGCGGC TACCTGTCGC

Xa23R1 GAAGGCTCGG GCCTGGTCAA CGAACTGGAT GTGGTCGAAG GCATGCAGTT CGACCGCGGC TACCTGTCGC

GPE 39 GAAGGCTCGG GCCTGGACAA CGAGCTGGAC GTGGTCGAGG GCATGCAGTT CGACCGCGGC TACCTGTCGC

MUS 060 GAAGGCTCGG GCCTGGACAA CGAGCTGGAC GTGGTCGAGG GCATGCAGTT CGACCGCGGC TACCTGTCGC

LMG 476 GAAGGCTCGG GCCTGGAGAA CGAGCTGGAC GTGGTCGAGG GCATGCAGTT CGATCGCGGC TACCTGTCGC

NCPPB4393 GAAGGCTCGG GCCTGGAGAA CGAGCTGGAC GTGGTCGAGG GCATGCAGTT CGATCGCGGC TACCTGTCGC

R1 GAAGGCTCGG GCCTGGAGAA CGAGCTGGAC GTGGTCGAGG GCATGCAGTT CGACCGCGGC TACCTGTCGC

XCV85-10 GAAGGCTCGG GCCTGGAAAA CGAGCTGGAC GTGGTCGAGG GCATGCAGTT CGACCGCGGC TACCTCTCCC

7001

GPE PC73 CATACTTCAT CAACAACCAG CAGAGCCAGT CGGCCGACTT GGACGATCCG TTCATCCTGC TGCACGACAA

Xa23R1 CATACTTCAT CAACAACCAG CAGAGCCAGT CGGCCGACTT GGACGATCCG TTCATCCTGC TGCACGACAA

GPE 39 CGTACTTCAT CAACAACCAG CAGAGCCAGT CGGCCGACCT GGACGATCCG TTCATCCTGC TGCACGACAA

MUS 060 CGTACTTCAT CAACAACCAG CAGAGCCAGT CGGCCGACCT GGACGATCCG TTCATCCTGC TGCACGACAA

LMG 476 CGTACTTCAT CAACAACCAG CAGAGCCAGC AGGCCGACCT GGACGACCCG TTCGTGCTGC TGCACGACAA

NCPPB4393 CGTACTTCAT CAACAACCAG CAGAGCCAGC AGGCCGACCT GGACGACCCG TTCGTGCTGC TGCACGACAA

R1 CGTACTTCAT CAACAACCAG CAGAGCCAGC AGGCCGACCT GGACGACCCG TTCGTGCTGC TGCACGACAA

XCV85-10 CGTACTTCAT CAACAACCAG CAGAGCCAGT CGGCCGACCT GGACGACCCG TTCATCCTGC TGCACGACAA

7071

GPE PC73 GAAGATCTCC AACGTGCGTG ACCTGCTGCC GTTGCTGGAA GGCGTGGCCA AGGCCGGCAA GCCGCTGCTG

Xa23R1 GAAGATCTCC AACGTCCGTG ACCTGCTGCC GTTGCTGGAA GGCGTGGCCA AGGCCGGCAA GCCGCTGCTG

GPE 39 GAAGATCTCC AACGTGCGTG ACCTGCTGCC GCTGCTGGAA GGCGTGGCCA AAGCGGGTAA GCCGCTGCTG

MUS 060 GAAGATCTCC AACGTGCGTG ACCTGCTGCC GTTGCTGGAA GGCGTGGCCA AAGCGGGCAA GCCGCTGCTG

LMG 476 GAAGATCTCC AACGTGCGTG ACCTGCTGCC GGTGCTGGAA GGCGTGGCCA AGGCCGGCAA GCCGCTGCTG

NCPPB4393 GAAGATCTCC AACGTGCGTG ACCTGCTGCC GGTGCTGGAA GGCGTGGCCA AGGCCGGCAA GCCGCTGCTG

R1 GAAGATCTCC AACGTGCGTG ACCTGCTGCC GGTGCTGGAA GGCGTGGCCA AGGCCGGCAA GCCGCTGCTG

XCV85-10 GAAGATCTCC AACGTGCGTG ACCTGCTGCC CGTGCTGGAA GGCGTGGCCA AGGCCGGCAA GCCGCTGCTG

7141

GPE PC73 ATCGTCGCCG AGGAAGTGGA AGGCGAGGCG CTGGCGACTC TGGTAGTCAA CACCATTCGT GGCATCGTCA

Xa23R1 ATAGTCGCCG AGGAAGTGGA AGGTGAGGCG CTGGCAACCT TGGTGGTCAA CACCATTCGT GGCATCGTCA

GPE 39 ATCGTCGCCG AGGAAGTGGA AGGCGAGGCG CTGGCGACCC TGGTGGTCAA CACCATCCGT GGCATCGTCA

MUS 060 ATCGTCGCCG AGGAAGTGGA AGGTGAGGCG CTGGCGACCC TGGTGGTCAA CACCATCCGT GGCATCGTCA

LMG 476 ATCGTTGCCG AGGAAGTGGA AGGCGAGGCG CTGGCGACCC TGGTGGTCAA CACCATCCGT GGCATCGTCA

NCPPB4393 ATCGTTGCCG AGGAAGTGGA AGGCGAGGCG CTGGCGACCC TGGTGGTCAA CACCATCCGT GGCATCGTCA

R1 ATCGTCGCCG AGGAAGTGGA AGGCGAGGCG CTGGCGACCC TGGTGGTCAA CACCATCCGC GGCATCGTCA

XCV85-10 ATCGTCGCCG AAGAAGTCGA AGGCGAAGCG CTGGCGACCC TGGTGGTCAA CACCATCCGC GGCATCGTCA

7211

GPE PC73 AGGTCGTGGC GGTCAAGGCT CCTGGCTTCG GCGACCGCCG CAAGGCGATG CTGGAAGACA TGGCGGTGCT

Xa23R1 GGGTCGTGGC GGTCAAGGCT CCTGGCTTCG GCGACCGCCG CAAGGCGATG CTGGAAGACA TGGCGGTGCT

GPE 39 AGGTCGTGGC GGTCAAGGCG CCGGGCTTCG GCGACCGTCG CAAGGCGATG TTGGAAGACA TGGCCGTGCT

MUS 060 AGGTCGTGGC GGTCAAGGCG CCGGGCTTCG GCGACCGTCG CAAGGCGATG TTGGAAGACA TGGCCGTGCT

LMG 476 AGGTCGTGGC GGTCAAGGCC CCGGGCTTCG GCGACCGTCG CAAGGCGATG CTGGAAGACA TGGCCGTGCT

NCPPB4393 AGGTCGTGGC GGTCAAGGCC CCGGGCTTCG GCGACCGTCG CAAGGCGATG CTGGAAGACA TGGCCGTGCT

R1 AGGTCGTGGC GGTCAAGGCC CCGGGCTTCG GCGACCGTCG CAAGGCGATG CTGGAAGACA TGGCCGTGCT

XCV85-10 AGGTCGTGGC CGTCAAGGCG CCGGGCTTCG GCGACCGTCG CAAGGCGATG CTGGAAGACA TGGCCGTGCT

7281

GPE PC73 GACTGGCGGC ACCGTGATCT CCGAGGAGGT GGGTCTGTCC CTCGAGAAGG CGACCCTGAA GGATCTGGGC

Xa23R1 GACTGGCGGC ACCGTGATCT CCGAGGAGGT GGGTCTGTCC CTCGAGAAGG CGACCCTGAA GGATCTGGGC

GPE 39 GACCGGCGGC ACCGTGATCT CCGATGAAGT GGGCCTGTCG CTGGAGAAGG CTACCCTCAA GGATCTGGGC

MUS 060 GACCGGCGGC ACCGTGATCT CCGATGAAGT GGGCCTGTCG CTGGAGAAGG CGACCCTCAA GGATCTGGGC

LMG 476 GACCGGCGGC ACCGTGATCT CCGAGGAAGT GGGCCTGGCG CTGGAGAAGG CGACCATCAA GGACCTGGGC

NCPPB4393 GACCGGCGGC ACCGTGATCT CCGAGGAAGT GGGCCTGGCG CTGGAGAAGG CGACCATCAA GGACCTGGGC

R1 GACCGGCGGC ACCGTGATCT CCGAGGAAGT GGGCCTGGCG CTGGAGAAGG CGACCATCAA GGACCTGGGC

XCV85-10 GACCGGCGGT ACCGTGATCT CCGAGGAAGT GGGCCTGGCG CTTGAGAAGG CGACCATCAA GGACCTGGGC

7351

GPE PC73 CGCGCCAAGA AGGTACAGGT TTCCAAGGAG AACACCACCA TCATCGATGG CGTCGGCGAT ACGGCCGCGA

Xa23R1 CGCGCCAAGA AGGTGCAGGT CTCCAAGGAG AACACCACCA TCATCGATGG CGTCGGCGAT ACGGCCGCGA

GPE 39 CGCGCCAAGA AGGTGCAGGT CTCCAAAGAA AACACCACCA TCATCGATGG CGTCGGCGAT ACCGCCGGGA

MUS 060 CGCGCCAAGA AGGTGCAGGT TTCCAAAGAA AACACCACCA TCATCGATGG CGTCGGCGAT ACCGCCGGGA

LMG 476 CGCGCCAAGA AGGTGCAGGT CTCCAAGGAG AACACCACCA TCATCGACGG CGCTGGCGAC ACCTCGGCGA

NCPPB4393 CGCGCCAAGA AGGTGCAGGT CTCCAAGGAG AACACCACCA TCATCGACGG CGCTGGCGAC ACCTCGGCGA

R1 CGCGCCAAGA AGGTGCAGGT CTCCAAGGAG AACACCACCA TCATCGACGG CGCCGGCGAC ACCTCGGCGA

XCV85-10 CGCGCCAAGA AGGTGCAGGT CTCCAAGGAA AACACCACCA TCATCGACGG CGCCGGCGAC ACCGCGGCGA

7421

GPE PC73 TCGAGTCGCG CATCAAGCAG ATCGAATTGC AGATTGCCGA GACCTCCTCC GACTACGACA AGGAGAAGTT

Xa23R1 TCGAGTCGCG CATCAAGCAG ATCGAATTGC AGATTGCCGA GACCTCCTCC GACTACGACA AGGAGAAGCT

GPE 39 TCGAGTCGCG CATCAAGCAG ATCGAATCGC AGATCGCCGA GACCTCGTCG GACTACGACA AAGAGAAGCT

MUS 060 TCGAGTCGCG CATCAAGCAG ATCGAATCGC AGATCGCCGA GACCTCGTCG GACTACGACA AAGAGAAGCT

LMG 476 TCGAATCGCG CATCAAGCAG ATCAAGGCGC AGATCGAAGA GACCTCGTCG GATTACGACC GCGAGAAGCT

NCPPB4393 TCGAATCGCG CATCAAGCAG ATCAAGGCGC AGATCGAAGA GACCTCGTCG GATTACGACC GCGAGAAGCT

R1 TCGAGTCGCG CATCAAGCAG ATCAAGGCGC AGATCGAAGA GACCTCTTCG GACTACGACC GCGAGAAGCT

XCV85-10 TCGAATCGCG CGTGGGCCAG ATCAAGACCC AGATCGAAGA CACCTCGTCC GATTACGACC GTGAGAAGCT

7491

GPE PC73 GCAGGAGCGC ATGGCCAAGC TGGCCGGCGG TGTGGCGGTG ATCAAGGTCG GTGCTGCGAC CGAAATCGAG

Xa23R1 GCAGGAGCGC ATGGCCAAGC TGGCCGGCGG CGTGGCGGTG ATCAAGGTCG GTGCTGCGAC CGAAATCGAG

GPE 39 GCAAGAGCGC ATGGCCAAGC TGGCTGGCGG CGTGGCGGTG ATCAAGGTCG GCGCTGCCAC CGAGATCGAA

MUS 060 GCAAGAGCGC ATGGCCAAGC TGGCTGGCGG CGTGGCGGTG ATCAAGGTCG GCGCTGCCAC CGAGATCGAA

LMG 476 GCAGGAGCGC GTGGCCAAGC TGGCCGGCGG CGTGGCGGTG ATCAAGGTCG GTGCCTCCAC CGAGATCGAG

NCPPB4393 GCAGGAGCGC GTGGCCAAGC TGGCCGGCGG CGTGGCGGTG ATCAAGGTCG GTGCCTCCAC CGAGATCGAG

R1 GCAGGAGCGC GTGGCCAAGC TGGCCGGCGG CGTGGCGGTG ATCAAGGTCG GTGCCTCGAC CGAGATCGAG

XCV85-10 GCAGGAGCGC GTGGCCAAGC TGGCCGGTGG CGTTGCAGTG ATCAAGGTCG GCGCCTCGAC CGAAATCGAA

7561

GPE PC73 ATGAAGGAAA AGAAGGCACG CGTCGAAGAT GCCCTGCACG CCACCCGTGC CGCAGTGGAA GAAGGCGTGG

Xa23R1 ATGAAGGAAA AGAAGGCACG CGTCGAAGAT GCCCTGCACG CCACCCGTGC CGCGGTGGAA GAAGGCGTGG

GPE 39 ATGAAAGAAA AGAAGGCGCG CGTCGAAGAC GCCCTGCACG CCACCCGTGC GGCGGTGGAA GAAGGCGTGG

MUS 060 ATGAAAGAAA AGAAGGCGCG CGTCGAAGAC GCCCTGCACG CCACCCGTGC GGCGGTGGAA GAAGGCGTGG

LMG 476 ATGAAGGAAA AGAAGGCGCG CGTCGAAGAC GCCCTGCACG CGACCCGTGC GGCGGTGGAA GAAGGCGTGG

NCPPB4393 ATGAAGGAAA AGAAGGCGCG CGTCGAAGAC GCCCTGCACG CGACCCGTGC GGCGGTGGAA GAAGGCGTGG

R1 ATGAAGGAAA AGAAGGCGCG CGTCGAAGAC GCCCTGCACG CGACCCGTGC GGCGGTGGAA GAAGGCGTGG

XCV85-10 ATGAAGGAAA AGAAGGCACG CGTCGAAGAC GCCCTGCACG CCACCCGTGC AGCCGTCGAA GAAGGCGTGG

7631

GPE PC73 TCCCCGGCGG CGGCGTGGCG CTGGTGCGCG CGCTGAGCGC GATCGGTTCG CTCACGGGCG ACAATGAAGA

Xa23R1 TCCCCGGCGG CGGCGTGGCG CTGGTGCGCG CGCTGAGCGC GATCGGTTCG CTCACGGGCG ACAATGAAGA

GPE 39 TCCCCGGCGG TGGCGTGGCC CTGGTGCGTG CGTTGAGCGC GATCGGTTCG CTCAAGGGCG ATAACGAAGA

MUS 060 TCCCCGGCGG CGGCGTGGCC CTGGTGCGTG CGTTGAGCGC GATCGGTTCG CTCAAGGGCG ATAACGAAGA

LMG 476 TCCCGGGCGG CGGCGTGGCG CTGGTGCGCG CGCTGAGCGC GATCGGCGAG CTGAAGGGCG CCAATGAAGA

NCPPB4393 TCCCGGGCGG CGGCGTGGCG CTGGTGCGCG CGCTGAGCGC GATCGGCGAG CTGAAGGGCG CCAATGAAGA

R1 TCCCGGGCGG CGGCGTGGCG CTGGTGCGCG CGCTGAGCGC GATCGGCGAG CTGAAGGGCG CCAATGAAGA

XCV85-10 TCCCGGGCGG CGGCGTGGCC CTGGTGCGTG CGCTGGTGGC CGTGGGCGAG CTCAAGGGCG CCAACGAAGA

7701

GPE PC73 TCAGACCCAC GGCATCCAGA TCGCGCTGCG CGCGATGGAA GCGCCGCTGC GCGCGATCGT CACCAACGCT

Xa23R1 TCAGACCCAC GGCATCCAGA TCGCGCTGCG CGCGATGGAA GCGCCGCTGC GCGCGATCGT CACCAACGCT

GPE 39 TCAGACCCAC GGTGTGCAGA TCGCGCTGCG TGCGATGGAA GCACCGCTGC GCGAGATCGT CACCAACGCC

MUS 060 TCAGACCCAC GGTGTGCAGA TCGCGCTGCG TGCGATGGAA GCACCGCTGC GCGAGATCGT CACCAACGCC

LMG 476 TCAGACCCAC GGCATCCAGA TCGCGCTGCG CGCGATGGAA GCGCCGCTGC GCGAGATCGT CACCAACGCC

NCPPB4393 TCAGACCCAC GGCATCCAGA TCGCGCTGCG CGCGATGGAA GCGCCGCTGC GCGAGATCGT CACCAACGCC

R1 TCAGACCCAC GGCATCCAGA TCGCGCTGCG CGCGATGGAA GCGCCGCTGC GCGAGATCGT CACCAACGCC

XCV85-10 CCAGACCCAC GGCATCCAGA TCGCCCTGCG CGCCATGGAA GCCCCGCTGC GCGAGATCGT GGCCAATGCC

7771

GPE PC73 GGTGAAGAAC CGTCGGTGAT TCTCAATAAG GTCAAGGAAG GCACCGGCAA CTTCGGCTAC GACGCTGCTA

Xa23R1 GGTGAAGAAC CGTCGGTGAT TCTCAATAAG GTCAAGGAAG GCACCGGCAA CTTCGGCTAC GACGCTGCTA

GPE 39 GGCGAAGAGC CGTCGGTGAT CCTCAACAAA GTCAAGGAAG GCACCGGCAA CTTCGGTTAC GACGCCGCCA

MUS 060 GGCGAAGAGC CGTCGGTGAT TCTCAACAAA GTCAAGGAAG GCACCGGCAA CTTCGGTTAC GACGCCGCCA

LMG 476 GGCGAAGAGC CGTCCGTGAT CCTCAACAAG GTCAAGGAAG GCAGCGGCAA CTTCGGCTAC AACGCCGCCA

NCPPB4393 GGCGAAGAGC CGTCCGTGAT CCTCAACAAG GTCAAGGAAG GCAGCGGCAA CTTCGGCTAC AACGCCGCCA

R1 GGCGAAGAGC CGTCCGTGAT CCTCAACAAG GTCAAGGAAG GCAGCGGCAA CTTCGGCTAC AACGCCGCCA

XCV85-10 GGCGAAGAGC CGTCCGTGAT CCTGAACAAG GTCAAGGAAG GCAGCGGCAA CTACGGCTAC AACGCCGCCA

7841

GPE PC73 AAGGCGAGTT CGGCGACATG GTCGCGTTCG GTATCCTGGA TCCGACCAAG GTGACCCGTT CGGCGCTGCA

Xa23R1 AAGGCGAGTT CGGCGACATG GTCGCGTTCG GTATCCTGGA TCCGACCAAG GTGACCCGTT CGGCGTTGCA

GPE 39 CCGGCCAGTT CGGCGACATG GTTGCGTTCG GTATCCTGGA TCCGACCAAG GTGACCCGTC TGGCCCTGCT

MUS 060 CCGGCCAGTT CGGCGACATG GTTGCGTTCG GTATCCTGGA TCCGACCAAG GTGACCCGTT TGGCGCTGCT

LMG 476 ACGGCGAGTT CGGCGACATG GTCGAGTTCG GCATCCTGGA TCCGACCAAG GTGACCCGTT CGGCGCTGCA

NCPPB4393 ACGGCGAGTT CGGCGACATG GTCGAGTTCG GCATCCTGGA TCCGACCAAG GTGACCCGTT CGGCGCTGCA

R1 ACGGCGAGTT CGGCGACATG GTCGAGTTCG GCATCCTGGA TCCGACCAAG GTGACCCGTT CGGCGCTGCA

XCV85-10 ACGGCGAGTT CGGCGACATG GTCGAATTCG GCATCCTGGA CCCGACCAAG GTGACCCGTT CGGCACTGCA

7911

GPE PC73 GAACGCTTCC TCGATCGCTG GCCTGATGAT CACCACCGAG GCGATGGTGG CCGAAGCGCC GAAGAAGGAC

Xa23R1 GAACGCCTCC TCGATCGCCG GCCTGATGAT CACCACCGAG GCGATGGTGG CCGAAGCGCC GAAGAAGGAA

GPE 39 GAATGCGTCC TCGATCGCCG GCCTGATGAT CACCACCGAA GCGATGGTGG CCGAAGCGCC GAAGAAGGAA

MUS 060 GAATGCGTCC TCGATCGCCG GCCTGATGAT CACCACCGAG GCGATGGTGG CCGAAGCGCC GAAGAAGGAA

LMG 476 GAACGCCGCC TCGATCGCCG GCCTGATGAT CACCACCGAA GCGATGGTGG CCGAGGCCCC GAAGAAGGAA

NCPPB4393 GAACGCCGCC TCGATCGCCG GCCTGATGAT CACCACCGAA GCGATGGTGG CCGAGGCCCC GAAGAAGGAA

R1 GAACGCCGCC TCGATCGCCG GCCTGATGAT CACCACCGAA GCGATGGTGG CCGAGGCCCC GAAGAAGGAA

XCV85-10 GAACGCCGCC TCGATCGCCG GCCTGATGAT CACCACCGAA GCCATGGTGG CCGATGCGCC GAAGAAGGAC

7981

GPE PC73 GAGCCGGCCG CGCCGGGCGG TATGGGCGGT GGCATGGGTG GCATGGGCGG CATGGATTTC TGAATGTCTG

Xa23R1 GAGCCGGCCG CGCCGGGCGG CATGGGCGGT GGCATGGGCG GCATGGGCGG CATGGATTTC TGAATGTCTG

GPE 39 GAGCCGGCCG CCGGCGGTGG CATGGGCGGT GGCATGGGCG GCATGGGCGG CATGGATTTC TGAATGTCTG

MUS 060 GAGCCGGCCG TCGGCGGTGG CATGGGCGGT GGCATGGGCG GCATGGGCGG CATGGATTTC TGAATGTCTG

LMG 476 GAGCCGGCCG CGCCGGGCGG CATGGGCGGC GGCATGGGCG GCATGGGCGG CATGGATTTC TGAATGTCTG

NCPPB4393 GAGCCGGCCG CGCCGGGCGG CATGGGCGGC GGCATGGGCG GCATGGGCGG CATGGATTTC TGAATGTCTG

R1 GAGCCGGCCG CTCCGGGCGG CATGGGCGGC GGCATGGGCG GCATGGGCGG CATGGATTTC TGAATGTCTG

XCV85-10 GAGCCGGCGA TGCCGGCC-- ----GGCGGC GGCATGGGCG GCATGGGCGG CATGGATTTC TGAATGTCCG

8051

GPE PC73 CGGAAACTAT TGAGAAGTTG GTTAAGGACA ACAAGATCGA GTTCGTCGAC CTGCGCTTCG TCGACCTGCG

Xa23R1 CGGAAACTAT TGAGAAGTTG GTTAAGGACA ACAAGATCGA GTTCGTCGAC CTGCGCTTCG TCGACCTGCG

GPE 39 CGGAAACTAT TGAGAAGCTG GTTAAGGACA ACAAGATCGA GTTCGTCGAC CTGCGCTTCG TCGACATGCG

MUS 060 CGGAAACTAT TGAGAAGCTG GTTAAGGACA ACAAGATCGA GTTCGTCGAC TTGCGCTTCG TCGACATGCG

LMG 476 CGGAAACTAT TGAGAAGCTG GTCAAGGACA ACAAGATCGA GTTCGTCGAC CTGCGCTTCG TCGACATGCG

NCPPB4393 CGGAAACTAT TGAGAAGCTG GTCAAGGACA ACAAGATCGA GTTCGTCGAC CTGCGCTTCG TCGACATGCG

R1 CGGAAACTAT TGAGAAGCTG GTCAAGGACA ACAAGATCGA GTTCGTCGAC CTGCGCTTCG TCGACATGCG

XCV85-10 TGGAAAATGT TGAAAAGCTG ATCAAGGACA ACAAGGTCGA GTTCGTCGAC CTGCGCTTCG TCGATATGCG

8121

GPE PC73 CGGCGTGCAG CAGCACGTCA CGTTCCCGGT CAGCATCATC GAGCCTGCAT TGTTCGAAGA GGGCAAGATG

Xa23R1 CGGCGTGCAG CAGCACGTCA CGTTCCCGGT CAGCATCATC GAGCCTGCAT TGTTCGAAGA GGGCAAGATG

GPE 39 CGGCGTGCAG CAGCACGTCA CGTTCCCGGT CAGCATCATC GAGCCGGCAC TGTTCGAAGA GGGCAAGATG

MUS 060 CGGCGTGCAG CAGCACGTCA CGTTCCCGGT CAGCATCATC GAGCCGGCAC TGTTCGAAGA GGGCAAGATG

LMG 476 CGGCGTGCAG CAGCACGTCA CCTTCCCGGT CAGCATCATC GAGCCGTCGC TGTTCGAGGA AGGCAAGATG

NCPPB4393 CGGCGTGCAG CAGCACGTCA CCTTCCCGGT CAGCATCATC GAGCCGTCGC TGTTCGAGGA AGGCAAGATG

R1 CGGCGTGCAG CAGCACGTCA CCTTCCCGGT CAGCATCATC GAGCCGGCGC TGTTCGAAGA AGGCAAGATG

XCV85-10 TGGTGTACAG CAGCACATCA CCTTTCCGGT CAATATCATC GAGCCGGCGC TGTTTGAAGA AGGCAAGATG

8191

GPE PC73 TTCGACGGCA GCTCGATCGC CGGCTGGAAG GGCATCAATG AGTCGGACAT GGTTCTGCTG CCGGATGCCG

Xa23R1 TTCGACGGCA GCTCGATCGC CGGCTGGAAG GGCATCAATG AGTCGGACAT GGTTCTGCTG CCGGATGCCG

GPE 39 TTCGACGGCA GCTCGATCGC CGGCTGGAAG GGCATCAATG AGTCGGACAT GGTCCTGCTG CCGGACGCTG

MUS 060 TTCGACGGCA GCTCGATCGC CGGCTGGAAG GGCATCAATG AGTCGGACAT GGTCCTGCTG CCGGACGCTG

LMG 476 TTCGACGGCA GCTCGATCGC CGGCTGGAAG GGCATCAACG AGTCGGACAT GGTCCTGCTG CCCGACGCCG

NCPPB4393 TTCGACGGCA GCTCGATCGC CGGCTGGAAG GGCATCAACG AGTCGGACAT GGTCCTGCTG CCCGACGCCG

R1 TTCGACGGCA GCTCGATCGC CGGCTGGAAG GGCATCAACG AGTCGGACAT GGTCCTGCTG CCCGACGCCG

XCV85-10 TTCGACGGCA GTTCGATCGC GGGCTGGAAG GGCATCAACG AGTCGGACAT GGTGTTGCTG CCGGACGCGG

8261

GPE PC73 ACACCGCTTT CCTCGACCCG TTCATGGCTG ACCCGACCCT GGTGCTGACC TGCGACATCC TCGATCCGGC

Xa23R1 ACACCGCCTT CCTCGACCCG TTCATGGCCG ACCCGACCCT GGTGCTGACC TGCGACATCC TCGATCCGGC

GPE 39 ACACGGCTTT CCTCGACCCG TTCATGGCCG ACCCGACCCT GGTGCTGACC TGCGACATCC TTGATCCGGC

MUS 060 ACACGGCTTT CCTCGACCCG TTCATGGCCG ACCCGACCCT GGTGCTGACC TGCGACATCC TTGATCCGGC

LMG 476 ACACCGCCTT CGTCGATCCG TTCATGGCCG ATCCGACCCT GGTGCTGACC TGCGACATCC TCGACCCGGC

NCPPB4393 ACACCGCCTT CGTCGATCCG TTCATGGCCG ATCCGACCCT GGTGCTGACC TGCGACATCC TCGACCCGGC

R1 ACACCGCCTT CGTCGATCCG TTCATGGCCG ATCCGACCCT GGTGCTGACC TGCGACATCC TCGACCCGGC

XCV85-10 GCACCGCGTA TCTGGATCCG TTCTTCGCCG ATCCGACCAT CGTGCTGACC TGCGACATCC TCGACCCGGC

8331

GPE PC73 GACCATGCAG AGCTATGCGC GCGATCCGCG CGGCGTGGCC AAGCGCGCCG AGGCTTATCT GAAGTCCAGC

Xa23R1 GACCATGCAG AGCTATGCGC GCGATCCGCG CGGCGTGGCC AAGCGCGCCG AGGCTTATCT GAAGTCCAGC

GPE 39 GACCATGCAG AGCTATGCGC GCGATCCGCG CGGCGTGGCC AAGCGCGCCG AGGCTTATCT GAAGTCCAGC

MUS 060 GACCATGCAT AGCTATGCGC GCGATCCGCG CGGCGTGGCC AAGCGCGCCG AGGCTTATTT GAAGTCCAGC

LMG 476 CACCATGCAG AGCTACGGCC GCGACCCGCG CGGCGTGGCC AAGCGCGCCG AGGCCTACCT GAAGTCCAGC

NCPPB4393 CACCATGCAG AGCTACGGCC GCGACCCGCG CGGCGTGGCC AAGCGCGCCG AGGCCTACCT GAAGTCCAGC

R1 CACCATGCAG AGCTACGGCC GCGATCCGCG CGGCGTGGCC AAGCGCGCCG AGGCCTACCT GAAGTCCAGC

XCV85-10 CACCATGCAG AGCTATGAGC GCGACCCGCG CGGCATCGCC AAGCGCGCCG AGGCCTACCT GAAGTCCTCC

8401

GPE PC73 GGCATCGCCG ATCAGGCGTT CTTCGGCCCG GAGCCGGAAT TCTTCATCTT CGACGGCGTG CGTTTCGGCA

Xa23R1 GGCATCGCCG ATCAGGCGTT CTTCGGCCCG GAGCCGGAAT TCTTCATCTT CGACGGCGTG CGTTTCGGCA

GPE 39 GGTATCGCCG ATCAGGCGTT CTTCGGCCCG GAGCCGGAAT TCTTCATCTT CGACGGCGTG CGTTTCGGCA

MUS 060 GGTATCGCCG ATCAGGCGTT CTTCGGCCCG GAGCCGGAAT TCTTCATCTT CGACGGCGTG CGTTTCGGCA

LMG 476 GGCATCGCCG ACCAGGCGTT CTTCGGCCCG GAGCCGGAAT TCTTCATCTT CGACGGCGTG CGCTTCGGCA

NCPPB4393 GGCATCGCCG ACCAGGCGTT CTTCGGCCCG GAGCCGGAAT TCTTCATCTT CGACGGCGTG CGCTTCGGCA

R1 GGCATCGCCG ACCAGGCGTT CTTCGGCCCG GAGCCGGAAT TCTTCATCTT CGACGGCGTG CGCTTCGGCA

XCV85-10 GGCACCGCAG ACCAGGCGTT CTTCGGCCCG GAGCCGGAAT TCTTCATCTT CGATTCGGTG CGCTTTGCCA

8471

GPE PC73 ACGACATGGG CCACACCTTC TTCAAGATCG ATTCGGAAGA AGCGGCTTGG AGCAGCGGCA GCAAGATCGA

Xa23R1 ACGACATGGG CCACACCTTC TTCAAGATCG ATTCGGAAGA AGCGGCGTGG AGCAGCGGCA GCAAGATCGA

GPE 39 ACGACATGGG CCACACCTTC TTCAAGATCG ACTCGGAAGA AGCGGCCTGG AGCAGCGGCA GCAAGATCGA

MUS 060 ACGACATGGG CCACACCTTC TTCAAGATCG ATTCCGAGGA AGCGGCGTGG AGCAGCGGCA GCAAGATCGA

LMG 476 ACGAGATGGG CCACACCTTC TTCAAGATCG ATTCGGAAGA GGCAGCCTGG AGCAGCGGCA GCAAGATCGA

NCPPB4393 ACGAGATGGG CCACACCTTC TTCAAGATCG ATTCGGAAGA GGCAGCCTGG AGCAGCGGCA GCAAGATCGA

R1 ACGAGATGGG CCACACCTTC TTCAAGATCG ATTCGGAAGA GGCGGCCTGG AGCAGCGGCA GCAAGATCGA

XCV85-10 ACGACATGGG CCACACCTTC TTCCAGGTGG GTTCGGAAGA GGCGGCCTGG AACACCGGCG AAAAGTACGA

8541

GPE PC73 GGGTGGCAAC AGCGGTTATC GTCCGGCGGT CAAGGGCGGC TACTTCCCGG TGCCGCCGAC CGACTCGCTG

Xa23R1 GGGTGGCAAC AGCGGTTATC GTCCGGCGGT CAAGGGTGGC TACTTCCCGG TGCCGCCGAC CGACTCGCTG

GPE 39 AGGCGGTAAC AGCGGTTATC GTCCGGCGGT CAAGGGTGGC TACTTCCCGG TGCCGCCGAC CGACTCGCTG

MUS 060 AGGCGGTAAC AGCGGTTATC GTCCGGCGGT CAAGGGTGGC TACTTCCCGG TGCCGCCGAC CGACTCGCTG

LMG 476 AGGCGGCAAC AGCGGCTACC GTCCGGCGGT CAAGGGCGGC TACTTCCCGG TGCCGCCTAC CGACTCGCTG

NCPPB4393 AGGCGGCAAC AGCGGCTACC GTCCGGCGGT CAAGGGCGGC TACTTCCCGG TGCCGCCTAC CGACTCGCTG

R1 AGGCGGCAAC AGCGGCTACC GTCCGGCGGT CAAGGGCGGC TACTTCCCGG TCCCGCCGAC CGACTCGCTG

XCV85-10 CGGCGGCAAC AGCGGTTACC GCCCGGGCGT GAAGGGCGGC TACTTCCCGG TTCCGCCGAC CGACAGCCTG

8611

GPE PC73 CAGGATCTGC GCGCGGAAAT GTGCAAGACC CTGGAGCAGG TCGGCATCGA GGTCGAAGTA CACCACCACG

Xa23R1 CAGGACCTGC GCGCGGAAAT GTGCAAGACC CTGGAGCAGG TCGGCATCGA GGTCGAAGTG CACCACCACG

GPE 39 CAGGATCTGC GCGCGGAAAT GTGCAAGACC CTGGAGCAGG TCGGCATCGA AGTCGAGGTG CACCACCACG

MUS 060 CAGGACCTGC GCGCGGAAAT GTGCAAGACC CTGGAGCAGG TCGGCATCGA AGTCGAGGTG CACCACCACG

LMG 476 CAGGACCTGC GCGCCGAGAT GTGCAAGACC CTGGAGCAGG TCGGCATCGA AGTCGAAGTG CACCACCACG

NCPPB4393 CAGGACCTGC GCGCCGAGAT GTGCAAGACC CTGGAGCAGG TCGGCATCGA AGTCGAAGTG CACCACCACG

R1 CAGGACCTGC GCGCCGAGAT GTGCAAGACC CTGGAGCAGG TCGGCATCGA AGTCGAAGTG CACCACCACG

XCV85-10 CACGACCTGC GCGCGGAAAT GATCAAGACG CTGGAACAGG TCGGCATCGA AACCGAGGTG CACCACCACG

8681

GPE PC73 AGGTCGCCAC CGCTGGCCAG TGCGAGATCG GCACCAAGTT CAACTCGCTG GTGAAGAAGG CCGACGAGCT

Xa23R1 AGGTCGCCAC CGCTGGTCAG TGCGAGATCG GCACCAAGTT CAACTCGCTG GTGAAGAAGG CCGACGAGCT

GPE 39 AGGTTGCCAC CGCTGGTCAG TGTGAAATCG GCACCAAGTT CAACTCGCTG GTGAAGAAGG CCGACGAGCT

MUS 060 AGGTTGCCAC CGCTGGTCAG TGTGAAATCG GCACCAAGTT CAACTCGCTG GTGAAGAAGG CCGACGAGCT

LMG 476 AAGTGGCCAC CGCCGGCCAG TGCGAGATCG GCACCAAGTT CAACTCGCTG GTGAAGAAGG CCGACGAGCT

NCPPB4393 AAGTGGCCAC CGCCGGCCAG TGCGAGATCG GCACCAAGTT CAACTCGCTG GTGAAGAAGG CCGACGAGCT

R1 AAGTGGCCAC CGCCGGCCAG TGCGAGATCG GCACCAAGTT CAACTCGCTG GTGAAGAAGG CCGACGAGCT

XCV85-10 AAGTGGCCAC CGCCGGCCAG TGCGAGATCG GCACCAAGTT CAGCTCGCTG GTGCAGAAGG CCGACGAACT

8751

GPE PC73 GATGACGATG AAGTACATCA TCAAGAACGT TGCCTACCGC AACGGCAAGA CTGTGACCTT CATGCCCAAG

Xa23R1 GATGACGATG AAGTACATCA TCAAGAACGT TGCCTACCGC AACGGCAAGA CTGTGACCTT CATGCCCAAG

GPE 39 GATGATGATG AAGTACATCA TCAAGAACGT CGCCTATCGC AACGGCAAGA CCGTGACCTT CATGCCCAAG

MUS 060 GATGATGATG AAGTACATCA TCAAGAACGT CGCCTATCGC AACGGCAAGA CCGTGACCTT CATGCCCAAG

LMG 476 GATGATGATG AAGTACATCA TCAAGAACGT CGCCTACCGC AACGGCAAGA CCGCGACCTT CATGCCCAAG

NCPPB4393 GATGATGATG AAGTACATCA TCAAGAACGT CGCCTACCGC AACGGCAAGA CCGCGACCTT CATGCCCAAG

R1 GATGATGATG AAGTACATCA TCAAGAACGT CGCCTACCGC AACGGCAAGA CCGCGACCTT CATGCCCAAG

XCV85-10 GCTGACGATG AAGTACATCA TCAAGAACGT CGCCTACCGC AACGGCAAGA CCGCCACCTT CATGCCCAAG

8821

GPE PC73 CCGATCGTCG GCGACAACGG CTCGGGCATG CATGTGCACC AGTCGCTGGC CAAGGGCGGC ACCAACCTGT

Xa23R1 CCGATCGTCG GCGACAACGG CTCGGGCATG CATGTGCACC AGTCGCTAGC CAAGGGCGGC ACCAATCTGT

GPE 39 CCGATCGTCG GCGACAACGG CTCGGGCATG CACGTGCACC AGTCGCTTGC CAAGGGCGGC GCCAACCTGT

MUS 060 CCGATCGTCG GCGACAACGG CTCGGGCATG CACGTGCACC AGTCGCTTGC CAAGGGCGGC GTCAACCTGT

LMG 476 CCGATCGTCG GCGACAACGG CTCGGGCATG CACGTGCACC AGTCGCTGGC CAAGGGCGGC ACCAACCTGT

NCPPB4393 CCGATCGTCG GCGACAACGG CTCGGGCATG CACGTGCACC AGTCGCTGGC CAAGGGCGGC ACCAACCTGT

R1 CCGATCGTCG GCGACAACGG CTCGGGCATG CACGTGCACC AGTCGCTGGC CAAGGGCGGC ACCAACCTGT

XCV85-10 CCCATCGTGG GCGACAACGG CTCGGGCATG CACGTGCACC AGTCGCTGGC CAAGGGCGGC GTCAACCTGT

8891

GPE PC73 TCTCCGGCGA CGGCTATGGT GGCCTGTCGC AGATGGCGCT GTGGTACATC GGCGGCATCT TCAAGCATGC

Xa23R1 TCTCCGGCGA CGGCTATGGC GGCCTGTCGC AGATGGCGCT GTGGTACATC GGCGGCATCT TCAAGCATGC

GPE 39 TCTCCGGCGA CGGCTATGGC GGCCTGTCGC AGATGGCGCT GTGGTACATC GGCGGCATTT TCAAGCATGC

MUS 060 TCTCCGGCGA CGGCTATGGC GGCCTGTCGC AGATGGCGTT GTGGTACATC GGCGGCATTT TCAAGCATGC

LMG 476 TCTCCGGCGA CGGCTACGGC GGCCTGTCGC AGCTGGCGCT GTGGTACATC GGCGGCATCT TCAAGCACGC

NCPPB4393 TCTCCGGCGA CGGCTACGGC GGCCTGTCGC AGCTGGCGCT GTGGTACATC GGCGGCATCT TCAAGCACGC

R1 TCTCCGGCGA CGGCTACGGC GGCCTGTCGC AGATGGCGCT GTGGTACATC GGCGGCATCT TCAAGCACGC

XCV85-10 TCACCGGCGA CGGCTACGGC GGCCTGTCGC AGCTGGCGCT GTGGTACATC GGCGGCATCT TCAAGCATGC

8961

GPE PC73 CAAGGCGATC AACGCCTTCA CCAACTCGGG CACCAACAGC TACAAGCGTC TGGTCCCGGG CTTCGAGGCG

Xa23R1 CAAGGCGATC AACGCCTTCA CCAACTCGGG CACCAACAGC TACAAGCGTC TGGTCCCGGG CTTCGAGGCG

GPE 39 CAAGGCGATC AATGCCTTCA CCAACTCCGG TACCAACAGC TACAAGCGTC TGGTCCCCGG CTTCGAAGCA

MUS 060 CAAGGCGATC AATGCCTTCA CCAACTCCGG TACCAACAGC TACAAGCGTC TGGTCCCCGG CTTCGAAGCA

LMG 476 CAAGGCGATC AACGCCTTCA CCAACTCCGG CACCAACAGC TACAAGCGCC TGGTCCCGGG CTTCGAGGCG

NCPPB4393 CAAGGCGATC AACGCCTTCA CCAACTCCGG CACCAACAGC TACAAGCGCC TGGTCCCGGG CTTCGAGGCG

R1 CAAGGCGATC AACGCCTTCA CCAATTCCGG CACCAACAGC TACAAGCGCC TGGTCCCGGG CTTCGAGGCG

XCV85-10 CCGCGCGATC AACGCCTTCG CCAACTCCGG CACCAACAGC TACAAGCGCC TGGTTCCGGG CTTCGAGGCG

9031

GPE PC73 CCGGTGATGC TGGCCTACTC GGCGCGCAAC CGCTCGGCCT CGTGCCGCAT CCCGTGGGTG TCCAACCCGA

Xa23R1 CCGGTGATGC TGGCCTACTC GGCGCGCAAC CGCTCGGCCT CGTGCCGCAT CCCGTGGGTG TCCAACCCGA

GPE 39 CCGGTGATGC TGGCCTACTC GGCGCGCAAC CGCTCGGCCT CGTGCCGCAT CCCGTGGGTG TCCAACCCGA

MUS 060 CCGGTGATGC TGGCCTACTC GGCGCGCAAC CGCTCGGCCT CGTGCCGCAT CCCGTGGGTG TCCAACCCGA

LMG 476 CCGGTGATGC TGGCCTACTC GGCGCGCAAC CGTTCGGCCT CGTGCCGCAT TCCGTGGGTG TCCAACCCGA

NCPPB4393 CCGGTGATGC TGGCCTACTC GGCGCGCAAC CGTTCGGCCT CGTGCCGCAT TCCGTGGGTG TCCAACCCGA

R1 CCGGTGATGC TGGCCTACTC GGCGCGCAAC CGTTCGGCCT CGTGCCGCAT TCCGTGGGTG TCCAACCCGA

XCV85-10 CCGGTGATGC TGGCCTATTC GGCGCGTAAC CGTTCGGCCT CGTGCCGCAT TCCGTGGGTC ACCAACCCGA

9101

GPE PC73 AGGCGCGCCG CATCGAGATC CGCTTCCCGG ATCCGCTGCA ATCCGGCTAC CTGACCTTTG CCGCGTTGAT

Xa23R1 AGGCGCGCCG CATCGAGATC CGCTTCCCGG ATCCGCTGCA ATCCGGCTAC CTGACCTTTG CCGCGTTGAT

GPE 39 AGGCCCGCCG CATCGAAATC CGCTTCCCGG ATCCGCTGCA GTCCGGCTAC CTGACCTTCG CCGCGCTGAT

MUS 060 AGGCCCGCCG CATCGAAATC CGCTTCCCGG ATCCGCTGCA GTCCGGCTAC CTGACCTTCG CCGCGCTGAT

LMG 476 AGGCGCGCCG CATCGAGATC CGCTTCCCGG ATCCGCTGCA GTCCGGCTAC CTGACCTTCG CCGCGCTGAT

NCPPB4393 AGGCGCGCCG CATCGAGATC CGCTTCCCGG ATCCGCTGCA GTCCGGCTAC CTGACCTTCG CCGCGCTGAT

R1 AGGCGCGCCG CATCGAGATC CGCTTCCCGG ATCCGCTGCA GTCCGGCTAC CTGACCTTCG CCGCGCTGAT

XCV85-10 AGGCGCGCCG CATCGAAATG CGCTTCCCCG ATCCGTTGCA GTCCGGCTAC CTGACCTTCA CCGCGCTGAT

9171

GPE PC73 GATGGCAGGC TTGGACGGTA TCAAGAACCA GATCGATCCA GGCGCGCCCA GCGACAAGGA TCTGTACGAC

Xa23R1 GATGGCAGGC TTGGACGGTA TCAAGAACCA GATCGATCCA GGCGCGCCCA GCGACAAGGA TCTGTACGAC

GPE 39 GATGGCCGGC CTGGACGGTA TCAAGAACCA GATCGACCCG GGCGCGCCCA GCGACAAGGA TCTGTACGAC

MUS 060 GATGGCCGGC CTGGACGGCA TCAAGAACCA GATCGACCCG GGCGCGCCCA GCGACAAGGA TCTGTACGAC

LMG 476 GATGGCCGGC CTGGACGGCA TCAAGAACCA GATCGACCCG GGTGCCCCCA GCGACAAGGA CCTGTACGAC

NCPPB4393 GATGGCCGGC CTGGACGGCA TCAAGAACCA GATCGACCCG GGTGCCCCCA GCGACAAGGA CCTGTACGAC

R1 GATGGCCGGC CTGGACGGCA TCAAGAACCA GATCGACCCG GGCGCGCCCA GCGACAAGGA CCTGTACGAC

XCV85-10 GATGGCCGGC CTGGACGGCA TCAAGAACCA GATCGACCCG GGCGCCCCGA GCGACAAGGA CCTGTACGAC

9241

GPE PC73 CTGCCGCCGG AAGAAGAGAA GAAAATCCCG CAGGTCTGCT CCAGCCTCGA CCAGGCGTTG GAAGCGCTTG

Xa23R1 CTGCCGCCGG AAGAAGAGAA GAAAATCCCG CAGGTCTGCT CCAGCCTCGA CCAGGCGTTG GAAGCGCTTG

GPE 39 CTGCCGCCTG AGGAAGAGAA GAAAATCCCG CAGGTCTGCT CGAGCCTGGA TCAGGCCTTG GAAGCGCTGG

MUS 060 CTGCCGCCTG AGGAAGAGAA GAAAATCCCG CAGGTCTGCT CGAGCCTGGA TCAGGCGCTG GAAGCGCTGG

LMG 476 CTGCCGCCGG AAGAAGAGAA GCAGATCCCT CAGGTGTGCT CCAGCCTCGA CCAGGCGCTG GAAGCGCTGG

NCPPB4393 CTGCCGCCGG AAGAAGAGAA GCAGATCCCT CAGGTGTGCT CCAGCCTCGA CCAGGCGCTG GAAGCGCTGG

R1 CTGCCGCCGG AAGAAGAGAA GCAGATCCCG CAGGTGTGCT CCAGCCTCGA CCAGGCGCTG GAAGCGCTGG

XCV85-10 CTGCCGCCGG AAGAAGAGAA GCTGATCCCG CAGGTGTGCT CCAGCCTGGA CCAGGCCCTG GAAGCGCTGG

9311

GPE PC73 ATGCCGACCG CGAGTTCCTC AAGGCCGGCG GCGTGTTCAC CGACGACTTC ATCGATGGCT ACATCGCGCT

Xa23R1 ATGCCGACCG CGAGTTCCTC AAGGCCGGCG GCGTGTTCAC CGACGACTTC ATCGATGGCT ACATCGCGCT

GPE 39 ATGCCGACCG TGAGTTCCTC AAGGCTGGCG GCGTGTTCAC CGACGACTTC ATCGATGGCT ACATCGCGCT

MUS 060 ATGCCGACCG TGAGTTCCTC AAGGCTGGCG GCGTGTTCAC CGACGACTTC ATCGATGGCT ACATCGCGCT

LMG 476 ACGCCGACCG CGAGTTCCTC AAGGCTGGCG GCGTGTTCAC CGACGACTTC ATCGACGGCT ACATCGCGCT

NCPPB4393 ACGCCGACCG CGAGTTCCTC AAGGCTGGCG GCGTGTTCAC CGACGACTTC ATCGACGGCT ACATCGCGCT

R1 ACGCCGACCG CGAGTTCCTC AAGGCCGGCG GCGTGTTCAC CGACGACTTC ATCGACGGCT ACATCGCGCT

XCV85-10 ACAAGGACCG CGAGTTCCTC AAGGCCGGCG GCGTGATGAG CGACGACTTC ATCGACGGCT ATATCGCGCT

9381

GPE PC73 GAAGATGCAG GAAGTGACCA AGTTCCGTGC GGCGACGCAC CCGCTGGAAT ATCAGCTGTA TTACGCCAAC

Xa23R1 GAAGATGCAG GAAGTGACCA AGTTCCGTGC GGCGACGCAC CCGCTGGAAT ATCAGCTGTA TTACGCCAAC

GPE 39 GAAGATGCAG GAAGTGACCA AGTTCCGTGC CGCGACTCAC CCACTGGAAT ATCAGCTGTA TTACGCCAAC

MUS 060 GAAGATGCAG GAAGTGACCA AGTTCCGTGC CGCGACTCAC CCACTGGAAT ATCAGCTGTA TTACGCCAAC

LMG 476 GAAGATGCAG GAAGTGACCA AGTTCCGCGC GGCCACGCAC CCGCTGGAAT ACCAGCTGTA CTACGCCAAC

NCPPB4393 GAAGATGCAG GAAGTGACCA AGTTCCGCGC GGCCACGCAC CCGCTGGAAT ACCAGCTGTA CTACGCCAAC

R1 GAAGATGCAG GAAGTGACCA AGTTCCGCGC GGCCACGCAC CCGCTGGAAT ACCAGCTGTA CTACGCCAAC

XCV85-10 GAAGATGCAG GAAGTCACCA AGTTCCGTGC TGCGACCCAC CCGTTGGAGT ACCAGCTGTA CTACGGCAAC

9451

GPE PC73 TGAATGGACG AGAACAAGAA GCGCGCGCTC GCGGCCGCCC TGAGTCAGAT CGAAAAGCAG TTCGGCAAGG

Xa23R1 TGAATGGACG AGAACAAGAA GCGCGCGCTC GCGGCCGCCC TGAGTCAGAT CGAAAAGCAG TTCGGCAAGG

GPE 39 TGAATGGACG AGAACAAGAA GCGCGCGCTC GCGGCCGCCC TGAGTCAGAT CGAAAAGCAG TTCGGCAAGG

MUS 060 TGAATGGACG AGAACAAGAA GCGCGCGCTC GCGGCCGCCC TGAGTCAGAT CGAAAAGCAG TTCGGCAAGG

LMG 476 TGAATGGACG AGAACAAGAA GCGCGCGCTT GCTGCCGCCC TGAGCCAGAT CGAGAAGCAG TTCGGCAAGG

NCPPB4393 TGAATGGACG AGAACAAGAA GCGCGCGCTT GCTGCCGCCC TGAGCCAGAT CGAGAAGCAG TTCGGCAAGG

R1 TGAATGGACG AGAACAAGAA GCGCGCGCTC GCCGCCGCCC TGAGCCAGAT CGAGAAGCAG TTCGGCAAGG

XCV85-10 TGAATGGACG AGAACAAGAA GCGCGCCCTT TCCGCCGCAC TGAGCCAGAT CGAAAAGCAA TTCGGCAAGG

9521

GPE PC73 GCTCGGTGAT GCGGATGGGC GATCGTGTCA TCGAAGCCGT GGAAGTGATT CCGACCGGTT CGCTGATGCT

Xa23R1 GCTCGGTGAT GCGGATGGGC GATCGTGTCA TCGAAGCCGT GGAAGTGATT CCGACCGGCT CGCTGATGCT

GPE 39 GCTCGGTGAT GCGGATGGGC GATCGTGTCA TCGAAGCCGT GGAAGTGATT CCGACCGGCT CGCTGATGCT

MUS 060 GCTCGGTGAT GCGGATGGGC GATCGTGTCA TCGAAGCCGT GGAAGTGATT CCGACCGGCT CGCTGATGCT

LMG 476 GCTCGGTGAT GCGGATGGGC GATCGCGTCA TCGAAGCGGT GGAAGTGATC CCGACCGGCT CGCTGATGCT

NCPPB4393 GCTCGGTGAT GCGGATGGGC GATCGCGTCA TCGAAGCGGT GGAAGTGATC CCGACCGGCT CGCTGATGCT

R1 GCTCGGTGAT GCGGATGGGC GATCGCGTCA TCGAAGCGGT GGAAGTGATT CCGACCGGCT CGCTGATGCT

XCV85-10 GCTCGGTCAT GCGCATGGGC GACCGCGTCA TCGAGGCAGT CGAAGTCATC CCGACCGGCT CGTTGATGCT

9591

GPE PC73 GGATATCGCG CTCGGCATCG GTGGCCTGCC GAAGGGCCGC GTGGTCGAGA TTTACGGTCC GGAATCCTCG

Xa23R1 GGATATCGCG CTCGGCATCG GTGGCCTGCC GAAGGGCCGT GTGGTCGAGA TCTACGGTCC GGAATCCTCG

GPE 39 GGATATCGCA CTCGGCATCG GCGGCCTGCC GAAGGGGCGG GTGGTCGAGA TCTACGGTCC GGAATCTTCG

MUS 060 GGATATCGCG CTCGGCATCG GCGGCCTGCC GAAGGGGCGG GTGGTCGAGA TCTACGGTCC GGAATCTTCG

LMG 476 GGACATCGCG CTGGGCATCG GCGGCCTGCC GAAGGGCCGC GTGGTCGAGA TCTACGGTCC GGAATCCTCG

NCPPB4393 GGACATCGCG CTGGGCATCG GCGGCCTGCC GAAGGGCCGC GTGGTCGAGA TCTACGGTCC GGAATCCTCG

R1 GGACATCGCG CTGGGCATCG GCGGCCTGCC GAAGGGTCGC GTGGTCGAGA TCTACGGTCC GGAATCCTCG

XCV85-10 GGATATCGCC CTGGGGATCG GCGGCCTGCC GAAGGGCCGC GTGGTCGAAA TCTACGGCCC GGAATCCTCC

9661

GPE PC73 GGCAAGACCA CCCTGACCCT GCAGGCCATC GCTCAGTGTC AGAAGAACGG CGGCACCGCT GCGTTCATTG

Xa23R1 GGCAAGACCA CCCTGACTCT GCAGGCCATC GCTCAGTGTC AGAAGAACGG CGGCACCGCT GCGTTCATCG

GPE 39 GGTAAGACCA CCCTGACCCT GCAGGCCATC GCGCAGTGTC AGAAGAACGG CGGCACCGCT GCGTTCATCG

MUS 060 GGTAAGACCA CCCTGACCCT GCAGGCCATC GCGCAGTGTC AGAAGAACGG CGGCACCGCT GCGTTCATCG

LMG 476 GGCAAGACCA CCCTGACCCT GCAGGCCATT GCGCAGTGCC AGAAGAACGG CGGCACCGCC GCGTTCATCG

NCPPB4393 GGCAAGACCA CCCTGACCCT GCAGGCCATT GCGCAGTGCC AGAAGAACGG CGGCACCGCC GCGTTCATCG

R1 GGCAAGACCA CCCTGACCCT GCAGGCCATC GCGCAGTGCC AGAAGAACGG CGGCACCGCC GCGTTCATCG

XCV85-10 GGCAAGACCA CCTTGACCCT GCAAGCCATC GCCCAGTGCC AGAAGAACGG CGGCACCGCT GCCTTCATCG

9731

GPE PC73 ATGCCGAACA TGCACTGGAC CCGGTCTACG CTGCCAAGCT GGGCGTCAAT GTCGACGAAT TGCTGCTGTC

Xa23R1 ATGCCGAACA TGCGCTGGAC CCGGTCTACG CCGCCAAGCT GGGCGTCAAT GTCGACGAAT TGCTGCTGTC

GPE 39 ACGCCGAGCA TGCGCTGGAC CCGGTCTACG CCGCCAAACT TGGCGTCAAT GTCGACGAAT TATTGCTGTC

MUS 060 ACGCCGAGCA TGCGCTGGAC CCGGTCTACG CCGCCAAACT TGGCGTCAAT GTCGACGAAT TATTGCTGTC

LMG 476 ACGCTGAGCA CGCGCTGGAC CCGATCTACG CCGCCAAGCT GGGCGTCAAC GTCGACGAAC TGCTGCTGTC

NCPPB4393 ACGCTGAGCA CGCGCTGGAC CCGATCTACG CCGCCAAGCT GGGCGTCAAC GTCGACGAAC TGCTGCTGTC

R1 ACGCCGAGCA CGCGCTGGAC CCGATCTACG CCGCCAAGCT GGGCGTCAAC GTCGACGAAC TGCTGCTGTC

XCV85-10 ACGCCGAGCA CGCGCTGGAC CCGATTTATG CGGCCAAGCT GGGCGTCAAC GTCGACGACC TGCTGCTGTC

9801

GPE PC73 GCAGCCGGAT ACCGGCGAAC AAGCGCTGGA AATCGCCGAT ATGCTGGTGC GCTCGGGGTC GGTGGACATC

Xa23R1 GCAGCCGGAT ACCGGCGAAC AAGCGCTGGA AATCGCCGAT ATGCTGGTGC GCTCGGGGTC GGTGGACATC

GPE 39 GCAGCCGGAT ACCGGCGAAC AAGCGCTGGA AATCGCCGAT ATGCTGGTGC GCTCGGGGTC GGTGGACATC

MUS 060 GCAGCCGGAT ACCGGCGAAC AAGCGCTGGA AATCGCCGAT ATGCTGGTGC GCTCGGGGTC GGTGGACATC

LMG 476 GCAGCCGGAC ACCGGCGAGC AGGCGCTGGA AATCGCCGAC ATGCTGGTGC GCTCGGGTTC GGTGGACATC

NCPPB4393 GCAGCCGGAC ACCGGCGAGC AGGCGCTGGA AATCGCCGAC ATGCTGGTGC GCTCGGGTTC GGTGGACATC

R1 GCAGCCGGAC ACCGGTGAGC AGGCGCTGGA AATCGCCGAC ATGCTGGTGC GTTCGGGGTC GGTGGACATC

XCV85-10 GCAGCCCGAT ACCGGTGAGC AGGCACTGGA AATTGCCGAC ATGCTGGTGC GCTCGGGTTC GGTTGATATC

9871

GPE PC73 GTGGTGATTG ACTCGGTCGC CGCGTTGACC CCAAAGGCCG AAATCGAGGG TGAAATGGGC GATCAACTGC

Xa23R1 GTGGTGATTG ACTCGGTCGC CGCGTTGACA CCAAAGGCCG AAATCGAGGG CGAAATGGGC GATCAACTGC

GPE 39 GTGGTGATTG ACTCGGTCGC CGCGTTGACT CCGAAAGCCG AAATTGAAGG GGAAATGGGC GATCAACTGC

MUS 060 GTGGTGATTG ACTCGGTCGC CGCGTTGACC CCGAAAGCCG AAATTGAAGG GGAAATGGGC GATCAACTGC

LMG 476 GTGGTGGTCG ACTCGGTCGC CGCGCTGACC CCCAAGGCCG AAATCGAAGG CGAGATGGGC GACCAGTTGC

NCPPB4393 GTGGTGGTCG ACTCGGTCGC CGCGCTGACC CCCAAGGCCG AAATCGAAGG CGAGATGGGC GACCAGTTGC

R1 GTGGTGGTCG ACTCGGTCGC CGCGCTGACC CCGAAGGCCG AAATCGAAGG CGAGATGGGC GATCAGCTGC

XCV85-10 GTGGTGGTCG ACTCGGTTGC CGCACTGACG CCAAAGGCGG AAATCGAAGG CGAGATGGGC GACCAGTTGC

9941

GPE PC73 CCGGCCTGCA GGCGCGTCTG ATGAGCCAGG CGCTGCGTAA GCTCACCGGC AACATCAAGC GCTCCAACAC

Xa23R1 CCGGCCTGCA GGCGCGTCTG ATGAGCCAGG CGCTGCGTAA GCTCACCGGC AACATCAAGC GCTCCAACAC

GPE 39 CCGGCCTGCA GGCGCGTCTG ATGAGCCAAG CGCTGCGTAA GCTCACCGGC AACATCAAGC GCTCCAATAC

MUS 060 CCGGCCTGCA GGCGCGTCTG ATGAGCCAAG CGCTGCGTAA GCTCACCGGC AACATCAAGC GCTCCAATAC

LMG 476 CGGGCCTGCA GGCCCGTCTG ATGAGCCAGG CGCTGCGCAA GCTCACCGGC AACATCAAGC GCTCCAACAC

NCPPB4393 CGGGCCTGCA GGCCCGTCTG ATGAGCCAGG CGCTGCGCAA GCTCACCGGC AACATCAAGC GCTCCAACAC

R1 CGGGTCTGCA GGCTCGCCTG ATGAGCCAGG CGCTGCGCAA GCTCACCGGC AACATCAAGC GCTCCAACAC

XCV85-10 CTGGTTTGCA GGCTCGCCTG ATGAGCCAGG CGCTGCGCAA GCTCACCGGC AATATCAAGC GCTCCAACAC

10011

GPE PC73 CCTGGTCGTC TTCATCAACC AGTTGCGCAT GAAGATCGGT GTGATGATGC CGGGGCAGAG CCCGGAAACC

Xa23R1 CCTGGTCGTC TTCATCAACC AGTTGCGCAT GAAGATCGGT GTGATGATGC CGGGGCAGAG CCCGGAAACC

GPE 39 CCTGGTCGTC TTCATCAATC AGTTGCGCAT GAAGATCGGC GTGATGATGC CGGGGCAGAG TCCTGAAACC

MUS 060 CCTGGTCGTC TTCATCAATC AGTTGCGCAT GAAGATCGGC GTGATGATGC CTGGCCAGAG TCCGGAAACC

LMG 476 CCTGGTGGTG TTCATCAACC AGCTGCGCAT GAAGATCGGC GTGATGATGC CGGGCCAGAG CCCGGAAACC

NCPPB4393 CCTGGTGGTG TTCATCAACC AGCTGCGCAT GAAGATCGGC GTGATGATGC CGGGCCAGAG CCCGGAAACC

R1 CCTGGTGGTG TTCATCAACC AGTTGCGCAT GAAGATCGGC GTGATGATGC CGGGCCAGAG CCCGGAAACC

XCV85-10 GCTGGTGGTC TTCATCAATC AGCTGCGCAT GAAGATCGGC GTCATGATGC CGGGCCAGAG CCCGGAAGTG

10081

GPE PC73 ACCACCGGCG GTAACGCACT GAAGTTTTAC GCATCGGTGC GGTTGGATAT CCGCCGCATC GGCGCGATCA

Xa23R1 ACCACCGGCG GTAACGCACT GAAGTTTTAC GCATCGGTGC GGTTGGATAT CCGCCGCATC GGCGCGATCA

GPE 39 ACCACCGGCG GTAACGCACT GAAGTTTTAC GCCTCGGTGC GGTTGGATAT CCGCCGCATC GGCGCGATCA

MUS 060 ACCACCGGCG GTAACGCACT GAAGTTCTAC GCCTCGGTGA GGTTGGATAT CCGCCGCATC GGCGCGATCA

LMG 476 ACCACCGGTG GCAACGCGCT GAAGTTCTAT GCCTCGGTGC GCCTGGACAT CCGCCGCATC GGCGCGATCA

NCPPB4393 ACCACCGGTG GCAACGCGCT GAAGTTCTAT GCCTCGGTGC GCCTGGACAT CCGCCGCATC GGCGCGATCA

R1 ACCACCGGCG GCAATGCGCT GAAGTTCTAC GCCTCGGTGC GCCTGGACAT CCGCCGCATC GGCGCGATCA

XCV85-10 ACCACCGGCG GCAACGCGCT GAAGTTCTAC GCCTCGGTGC GTCTGGATAT CCGCCGTATC GGTGCGATCA

10151

GPE PC73 AGAAGGGCGA TGAAATCATC GGCAACCAGA CCAAAATCAA GGTGGTCAAG AACAAGTTGG CTCCCCCGTT

Xa23R1 AGAAGGGCGA TGAAATCATC GGCAACCAGA CCAAAATCAA GGTGGTCAAG AACAAGTTGG CTCCCCCGTT

GPE 39 AGAAGGGCGA TGAAATCATC GGCAACCAGA CCAAAATCAA GGTGGTCAAG AACAAGCTGG CTCCCCCGTT

MUS 060 AGAAGGGCGA TGAAATCATC GGCAACCAGA CCAAAATCAA GGTGGTCAAG AACAAGCTGG CCCCCCCGTT

LMG 476 AGAAGGGCGA CGAGATCATC GGCAATCAGA CCAAGATCAA GGTCGTCAAG AACAAGCTGG CGCCGCCGTT

NCPPB4393 AGAAGGGCGA CGAGATCATC GGCAATCAGA CCAAGATCAA GGTCGTCAAG AACAAGCTGG CGCCGCCGTT

R1 AGAAGGGCGA CGAGATCATT GGCAACCAGA CCAAGATCAA GGTCGTCAAG AACAAGCTGG CGCCTCCGTT

XCV85-10 AGAAGGGCGA CGAGATCATC GGCAACCAGA CCAAGATCAA GGTGGTCAAG AACAAGCTGG CGCCTCCGTT

10221

GPE PC73 CAAACAGGTC GTCACCGAGA TCCTCTACGG CGAAGGCATC AGTCGCGAGG GCGAACTGAT CGACATGGGC

Xa23R1 CAAACAGGTC GTCACCGAGA TCCTCTACGG CGAAGGCATC AGCCGCGAGG GCGAACTGAT CGACATGGGC

GPE 39 CAAGCAGGTC GTCACCGAAA TCCTCTACGG TGAAGGCATC AGCCGCGAGG GCGAGCTGAT CGACATGGGT

MUS 060 CAAGCAGGTC GTCACCGAAA TCCTCTACGG TGAAGGCATC AGCCGCGAGG GCGAGCTGAT CGACATGGGT

LMG 476 CAAGCAGGTC GTCACCGAAA TCCTCTACGG CGAAGGCATC AGCCGCGAGG GTGAACTGAT CGACATGGGC

NCPPB4393 CAAGCAGGTC GTCACCGAAA TCCTCTACGG CGAAGGCATC AGCCGCGAGG GTGAACTGAT CGACATGGGC

R1 CAAGCAGGTC ATCACCGAAA TCCTCTACGG CGAAGGCATC AGCCGCGAGG GCGAACTGAT CGACATGGGT

XCV85-10 CAAGCAGGTC GTGACCGAAA TCCTGTATGG CGAAGGCATC AGCCGCGAGG GCGAATTGAT CGACATGGGC

10291

GPE PC73 GTGGAGGCCA AGCTGGTTGA AAAGGCCGGT GCCTGGTATA GCTACGGCAG CGAGCGCATC GGCCAGGGCA

Xa23R1 GTGGAGGCCA AGCTGGTTGA AAAGGCCGGT GCCTGGTATA GCTACGGCAG CGAGCGCATC GGCCAGGGCA

GPE 39 GTGGAAGCCA AACTGGTTGA AAAGGCGGGT GCTTGGTACA GCTACGGCAG CGAGCGCATC GGCCAGGGCA

MUS 060 GTGGAAGCCA AACTGGTTGA AAAGGCGGGT GCTTGGTACA GCTACGGCAG CGAGCGCATC GGCCAGGGCA

LMG 476 GTGGAAGCCA AGCTGGTCGA GAAGGCCGGT GCCTGGTACA GCTACGGCAG CGAGCGCATC GGCCAGGGCA

NCPPB4393 GTGGAAGCCA AGCTGGTCGA GAAGGCCGGT GCCTGGTACA GCTACGGCAG CGAGCGCATC GGCCAGGGCA

R1 GTGGAAGCCA AGCTGGTCGA GAAGGCCGGC GCCTGGTACA GCTACGGCAG CGAGCGCATC GGCCAGGGCA

XCV85-10 GTGGAAGCCA AGCTGGTCGA CAAGGCCGGC GCCTGGTACA GCTACGGCGA TGAGCGCATC GGGCAGGGCA

10361

GPE PC73 AGGACAACGC GCGCGGCTAC CTGCGCGACA ACCCTCAGGT TGCCGCCAAG TTGGAAAGCG AACTGCGCGA

Xa23R1 AGGACAACGC GCGCGGCTAC CTGCGCGACA ACCCTCAGGT TGCCGCCAAG TTGGAAAGCG AACTGCGCGA

GPE 39 AGGACAACGC TCGCGGCTAC CTGCGCGACA ACCCTCAGGT CGCGGCCAAG TTGGAAAGCG AACTACGCGA

MUS 060 AGGACAACGC TCGCGGCTAC CTGCGCGACA ACCCTCAGGT CGCGGCCAAG TTGGAAAGCG AACTACGCGA

LMG 476 AGGACAACGC CCGCGGTTAC CTGCGCGACA ACCCGCAGGT TGCGGCCAAG CTGGAAGGCG AGCTGCGCGA

NCPPB4393 AGGACAACGC CCGCGGTTAC CTGCGCGACA ACCCGCAGGT TGCGGCCAAG CTGGAAGGCG AGCTGCGCGA

R1 AGGACAACGC CCGCGGCTAC CTGCGCGACA ACCCGCAGGT CGCGGCCAAG CTGGAAAGCG AGCTGCGCGA

XCV85-10 AGGACAACGC GCGGACCTAC CTGCGCGACA ACCCGCAGGT TGCGACCCGG CTGGAAGCCG AGTTGCGTGA

10431

GPE PC73 GAAGTTCCAG CCCACGGAAA TCGCGCCCAG CCCAGCCGAT GCCGAAGACC TCGAGGCTTA

Xa23R1 GAAGTTCCAG CCCACGGAAA TCGCGCCCAG CCCAGCCGAT GCCGAAGACC TCGACGCTTA

GPE 39 GAAGTTCCAA CCCACGGAAA TCGCACCCAG CCAAGCCGAC GCCGAAGACC TGGAAGCGTA

MUS 060 GAAGTTCCAA CCCACGGAAA TCGCACCCAG CCAAGCCGAC GCCGAAGACC TCGAAGCGGA

LMG 476 GAAGTTCCAG CCCACGGAAA TCGCCCCGAG CGAGGCCGAG GCGGAAGACG ACGACGCGTA

NCPPB4393 GAAGTTCCAG CCCACGGAAA TCGCCCCGAG CGAGGCCGAG GCGGAAGACG ACGACGCGTA

R1 GAAGTTCCAG CCCACGGAGA TCGCTGCGAG CGAGGCCGAG GCGGAAGACG ACGACGCGTA

XCV85-10 GAAGTTCCAG CCTGCCGAA- --GCACCGCG CGAGGCCGGC GACGACGAAG ACAAGGAATA
